# Imaging exoplanets below the telescope stability limit

**DOI:** 10.1038/s41586-026-11213-z

**Published:** 2026-11-04

**Authors:** Rodrigo Ferrer-Chavez, Jason J. Wang, Katherine L. Bouman, William T. Freeman, Laurent Pueyo, Brandon Y. Feng, Marshall D. Perrin

**Affiliations:** 1 Department of Physics and Astronomy, Northwestern University, Evanston, IL USA; 2 Center for Interdisciplinary Exploration and Research in Astrophysics (CIERA), Northwestern University, Evanston, IL USA; 3 Departments of Computing and Mathematical Sciences, Electrical Engineering, and Astronomy, California Institute of Technology, Pasadena, CA USA; 4 Computer Science and Artificial Intelligence Laboratory, Massachusetts Institute of Technology, Cambridge, MA USA; 5 Space Telescope Science Institute, Baltimore, MD USA

**Keywords:** Exoplanets, Exoplanets, Astronomical instrumentation

## Abstract

Exoplanet imaging is the main method for detailed characterization of planets orbiting beyond 1 au (ref. ^1^) but it requires suppressing the bright glare of the star to see the faint planet light. As well as sophisticated instrumentation^2,3^, effective image processing is necessary for starlight suppression, which continues to be a substantial limitation^4^. Standard algorithms use reference images for starlight modelling and thus they are limited by inevitable telescope instabilities that change from reference to science observation^5,6^. Addressing this challenge will increase the number of planets amenable to direct imaging and will be a notable step towards imaging exo-Earths with future observatories^7^. Here we present a new physics-based method for starlight subtraction. Our method combines a multistep optimization with a flexible physical model to produce a starlight model, allowing modelling and compensation of instrument instabilities. We use automatic differentiation (autodiff)^8^ to optimize millions of model parameters. We demonstrate our method on observations from the James Webb Space Telescope (JWST) Near-InfraRed Camera (NIRCam)^9–11^ coronagraph instrument. We achieve sensitivity 2–15 times superior to standard techniques, including the first detection of 51 Eridani b (ref. ^12^) at 4.3 μm. This is the first time, to our knowledge, that such an approach has demonstrated substantially improved sensitivity on real, on-sky coronagraphic imaging. Our framework can be extended to other current and future ground-based and space-based observatories, including the Nancy Grace Roman Space Telescope and the Habitable Worlds Observatory (HWO), potentially relaxing the unprecedented stability requirements needed to make these a success.

## Abstract (web summary)

A new physics-based method combining the use of automatic differentiation to optimize millions of model parameters with a flexible physical model is described for starlight subtraction from real, on-sky coronagraphic images to greatly improve sensitivity.

## Main

To detect any planetary signals by direct imaging, the glare of the star (or stellar point spread function (PSF)) must be modelled and removed from our observations. Because even the brightest young giant planets are about 10^5^ times fainter than their host stars in the near-infrared, modelling the stellar PSF to the required precision is difficult. This is because of its strong dependence on factors such as stellar properties and instrumental aberrations. Until now, the most successful approaches build the PSF model empirically using a library of reference observations taken at other times or wavelengths or of other stars (for example, refs. ^13,14^).

**Fig. 1:**
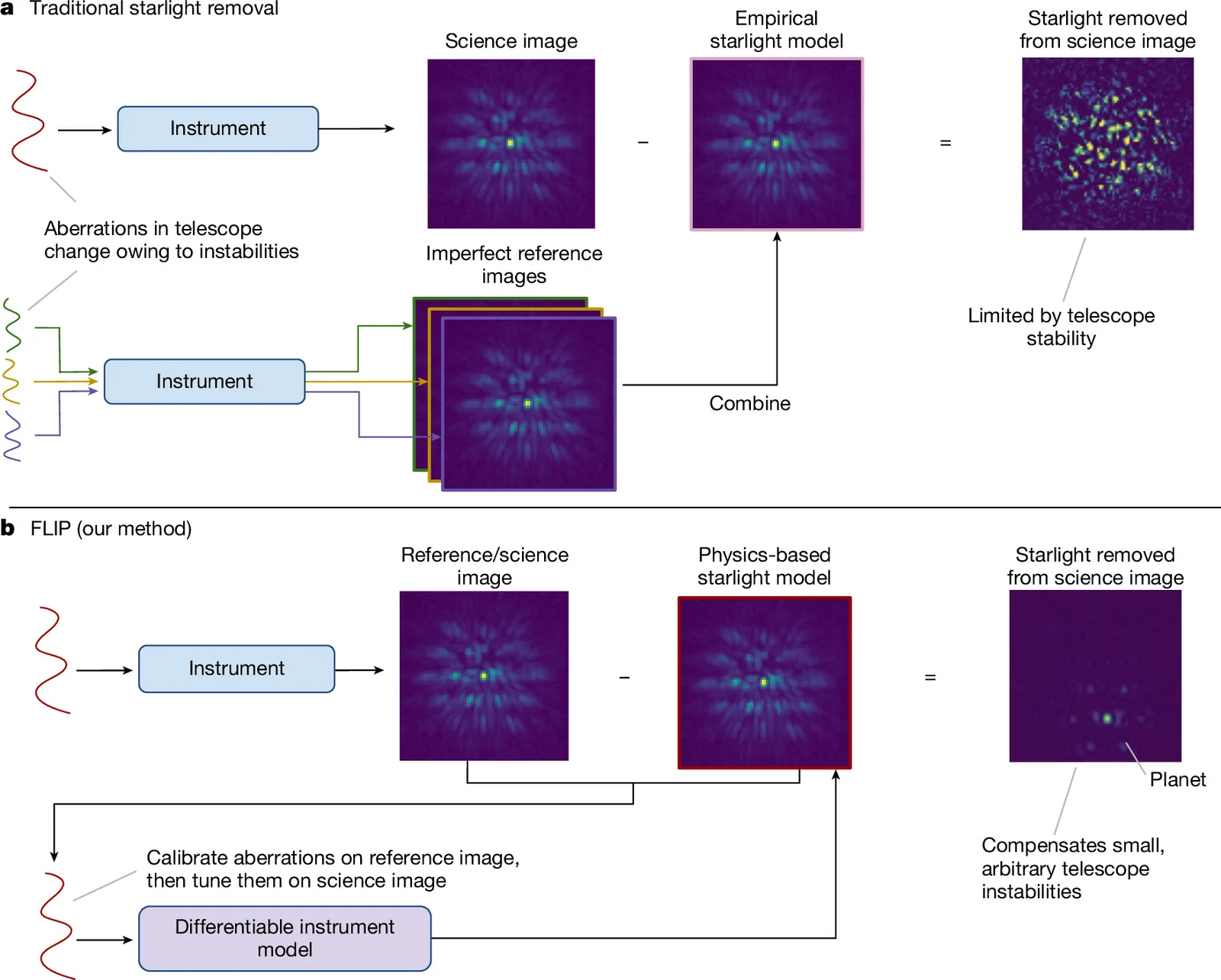
Schematic comparison of standard starlight subtraction and our method based on physical modelling, FLIP. **a**, Standard starlight subtraction. **b**, Our method based on FLIP. The stellar PSF is shaped by inevitable and imperfectly known aberrations in the telescope (including star–coronagraph misalignment and optical phase aberrations, represented by coloured curves to the left of the diagrams). Starlight subtraction methods based on empirical reference images are limited by telescope instabilities that cause changes in these aberrations, as these result in dissimilar reference PSFs (symbolized with curves and image outlines of different colours) and thus imperfect PSF subtraction when combining them into an empirical PSF model. Our method, FLIP, pre-calibrates an instrument model on reference images to reproduce the reference PSF and then solves for small changes in telescope aberrations that better explain the science image. This is represented by the optimization loop that tunes aberration estimates with arbitrary flexibility (and thus closer to the effective aberrations that produce the science PSF, represented by the red curves), beyond the limited aberration diversity provided by imperfect reference PSFs in standard approaches. This circumvents the stability limit of the instrument and enables imaging of fainter planets.

However, these empirical approaches are fundamentally limited by various sources of instabilities (Fig. 1). In space-based observatories, these mostly include telescope pointing errors and thermal drifts^6^, causing differences between reference and science PSFs and reducing our sensitivity^6,15^. Advances in PSF modelling that do not rely as heavily on observatory stability could have transformative implications for exoplanet imaging, for both current and future observatories. For example, one of the main challenges for future missions such as the HWO, with the ambitious goal of imaging Earth analogues around Sun-like stars (10^10^ brightness contrast), is achieving an extreme and unprecedented stability at the picometre level (100 to 1,000 times more stable than the JWST^5,16^).

An attractive alternative to starlight subtraction by means of empirical reference images is model-based PSF subtraction: directly modelling the physics of the instrument to reconstruct the stellar PSF. This approach has been noted for its potential to relax the stability requirements of the telescope and to reach the fundamental Poisson noise sensitivity limit^7^. Although promising, previous work investigating model-based PSF subtraction have noted formidable challenges given the complexity of optical systems that prevents accurate modelling^17–20^. Even in a stable space-based instrument such as JWST NIRCam, PSF subtraction with synthetic PSFs has only been used in extraordinary circumstances, in which empirical reference libraries are exceptionally low quality, and it has never produced sensitivity superior to standard techniques (for example, refs. ^21,22^).

Fortunately, nowadays, the computational challenges of model-based PSF subtraction can be mitigated with GPU-accelerated differentiable models. Differentiable models make use of autodiff^8^ to compute analytic derivatives even with millions of free parameters and thus greatly accelerate optimization problems. Differentiable models have been explored recently, for example, in non-coronagraphic modes of JWST^23^ and ground-based PSF reconstruction^24^. Preliminary experiments on simplified simulated coronagraphic data^25^ have suggested that differentiable coronagraphic models could have a large impact in exoplanet imaging.

Here we present a framework for coronagraphic exoplanet imaging that uses differentiable models for model-based PSF subtraction to deliver notable gains in exoplanet detection sensitivity. Our approach, Physics-based Learning for Imaging Planets (FLIP), incorporates a multistep optimization strategy with a flexible physical model informed by wavefront sensing data to achieve the PSF fidelity necessary for real data. We demonstrate this framework with real JWST NIRCam observations, providing the first, to our knowledge, successful application of differentiable model-based PSF subtraction on real, on-sky coronagraphic data. Our approach improves exoplanet detection limits by up to 15 times relative to published analyses that used standard pipelines. These improvements enable the recovery of planetary signals that were previously inaccessible to existing approaches. We demonstrate this by obtaining the first image of giant planet 51 Eridani b at 4.3 μm, which previously resulted in a non-detection with standard techniques^26^. These results show the potential of physics-based, autodiff-enabled algorithms to surpass limits set by telescope stability in current and future observatories.

## FLIP method overview

FLIP (Fig. 1) applies three main methodological innovations^23–25^ to on-sky space-based coronagraphic imaging for the first time: physics-based modelling, autodiff and a multistage optimization framework. At a high level, FLIP can be summarized as follows. We use a physics-based instrument model and wavefront sensing data to generate a simulated PSF of the star. Using gradient descent, we tune the parameters in our instrument model to match the real PSF of the star as observed on real data, minimizing the difference between the simulated star PSF and the real JWST NIRCam science image. Autodiff makes optimization by gradient descent computationally efficient. In our multistage optimization, we pre-calibrate our instrument model on a reference JWST NIRCam observation before tuning it on our science observation. Pre-calibration places our model close to an accurate solution, whereas tuning on the science image overcomes the differences between reference and science images owing to instrument instabilities. To mitigate overfitting of real planet signals, we inject fake planets on the science image and track their signal-to-noise ratio (SNR) during our optimization, stopping it when the SNR reaches a maximum or plateaus.

Our physical model consists of an astrophysical scene (a point source corresponding to the host star), an optical model and a detector model. Our JWST optical models are based on the nominal models provided in the open-source software STPSF^27^, the de facto JWST PSF modelling tool maintained by the Space Telescope Science Institute. These models are known to be imperfect, particularly for NIRCam coronagraphy, but they represent the current best characterization available of the optics and known aberrations. We implement this physical model in PyTorch^28^, following ref. ^25^, to make it differentiable and use autodiff in our gradient descent optimization. With this model as our initial condition, we solve for wavefront aberrations, the position and flux of the star and the pixel-level response of the detector.

Our multistage optimization framework is used to overcome model mismatch between our model and the real instrument, essentially improving the initial condition for our science image optimization. For pre-calibration, we choose a reference star similar to our science observation, typically one of the empirical reference images that would be used in traditional PSF subtraction methods. During our optimization on the science image, our fake planets are injected close to the sensitivity limit or at similar separation and brightness as the known planet in the image if there is one. They also serve as injection-recovery tests to quantify signal loss and sensitivity corrections in the final PSF-subtracted image. In this stage, we also slow down our optimization (scaling down the loss, and thus the gradient, during our gradient descent) and tune only a subset of the model parameters to further reduce the risk of planet overfitting. This end-to-end optimization, tuning around 10^6^ free model parameters with an Adam optimizer^29^, takes around 35 min in a normal NVIDIA A30 GPU. See Methods for details.

**Fig. 2:**
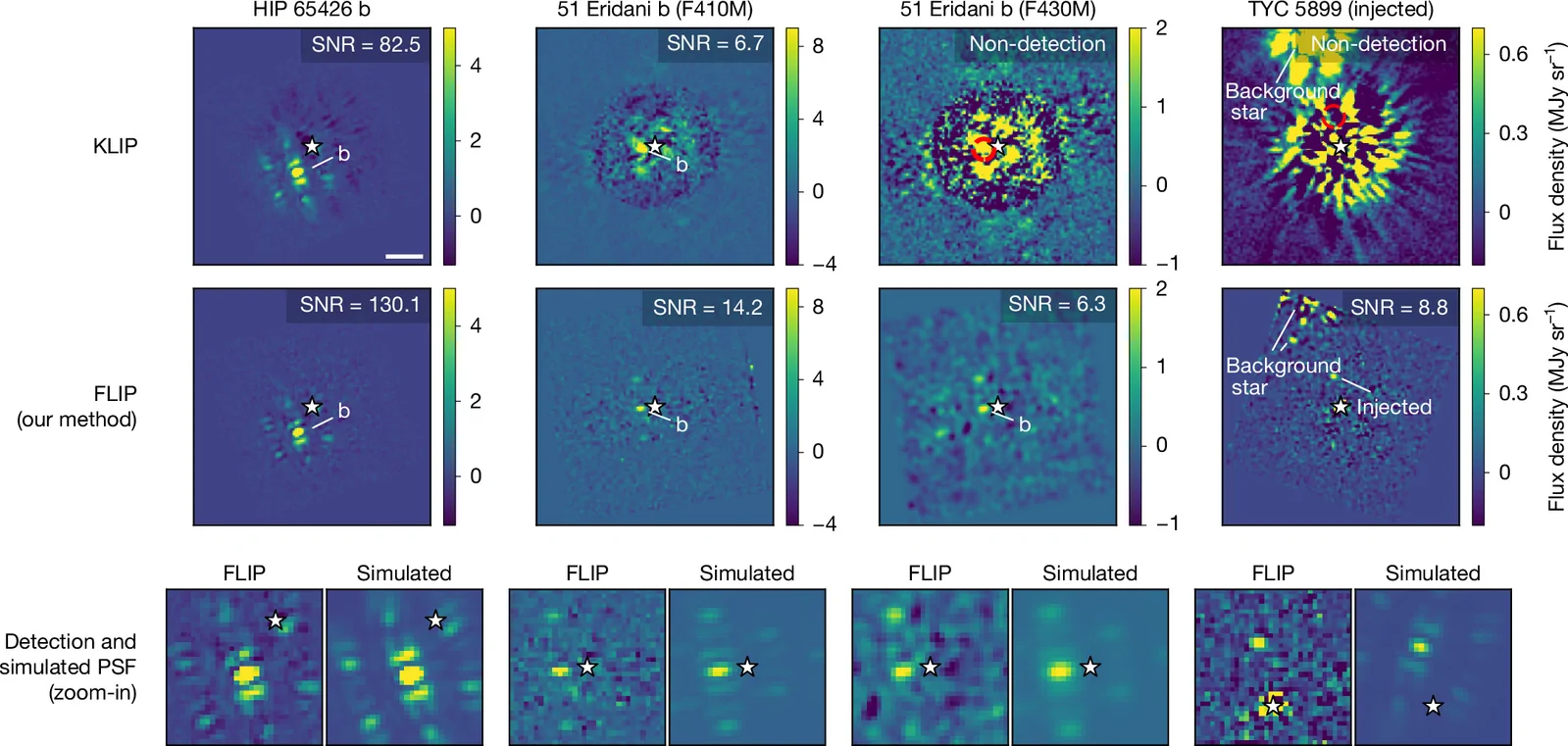
Stellar PSF subtraction comparison between our method FLIP and standard method KLIP. Top, observations reprocessed in this work with KLIP, using the same algorithm hyperparameters as in their respective publications. In all cases, our reprocessed KLIP results are comparable with the published results^26,31,32^. Middle, data processed with FLIP, showing the full field of view optimized over 80 × 80 pixels. Bottom, our detected companions with FLIP next to ideal simulated PSFs, to highlight the recovered morphology and to distinguish real point sources from PSF side lobes. The SNR of the detection is shown in the upper-right corner of each panel. Accounting for small sample statistics following ref. ^50^, the confidence of the detections from top left to bottom right correspond to 23.7*σ*, 5.7*σ*, non-detection, non-detection, 26.3*σ*, 8.8*σ*, 5.3*σ* and 7.7*σ*. The position of the star is marked with a star-shaped icon. In all panels, north is up and east is left. 51 Eridani F430M observations are convolved with a Gaussian kernel (*σ* = 1 pixel) to smooth out pixel-to-pixel noise (only in this panel is pixel-to-pixel noise comparable with the brightness of the companion). Scale bar, 1 arcsec.

We show the performance of FLIP on published JWST NIRCam data and compare it with the optimal PSF subtraction obtained in those publications with standard method Karhunen–Loève image processing (KLIP)^14^. We analyse observations of two star systems with known planets: HIP 65426 (ref. ^30^), first published in ref. ^31^, and 51 Eridani (ref. ^12^), originally published in (ref. ^26^). We also process an observation of TYC 5899, a star from a planet search survey with no known companions and with notable aberration drifts between reference and science observations^32^. We choose this target to showcase the exoplanet discovery potential of FLIP even when faced with long-term observatory instabilities. For HIP 65426 and TYC 5899, we use the observations in the F444W filter with the round 335R coronagraph mask and for 51 Eridani, we process two filters, F410M and F430M, both with the bar coronagraph (in the NARROW position)^33,34^. This choice of experiments shows the performance of FLIP across a wide range of stellar spectral types, coronagraphs, observational filters, reference star observation strategies and levels of observatory stability. Figure 2 shows comparisons of the PSF-subtracted observations using KLIP and our method FLIP.

FLIP achieves superior performance in all observations relative to KLIP, with large improvements in most cases. We obtain around 1.6 times better SNR on HIP 65426 b, 2.1 times better SNR on 51 Eridani b in F410M and a SNR = 6.3 detection of 51 Eridani b in F430M, which yielded a non-detection in the original publication (sensitivity was improved by a factor of about four at the location of the planet). For TYC 5899, we inject fake planets before PSF subtraction with both KLIP and FLIP to illustrate the sensitivity improvement. One of the injections is shown in Fig. 2, which we recover at high importance, whereas it is far from being detected with KLIP. Furthermore, there is a bright, nearby star in the TYC 5899 scene that is still visible after our PSF subtraction, but it has been distorted. See Methods for details.

**Fig. 3:**
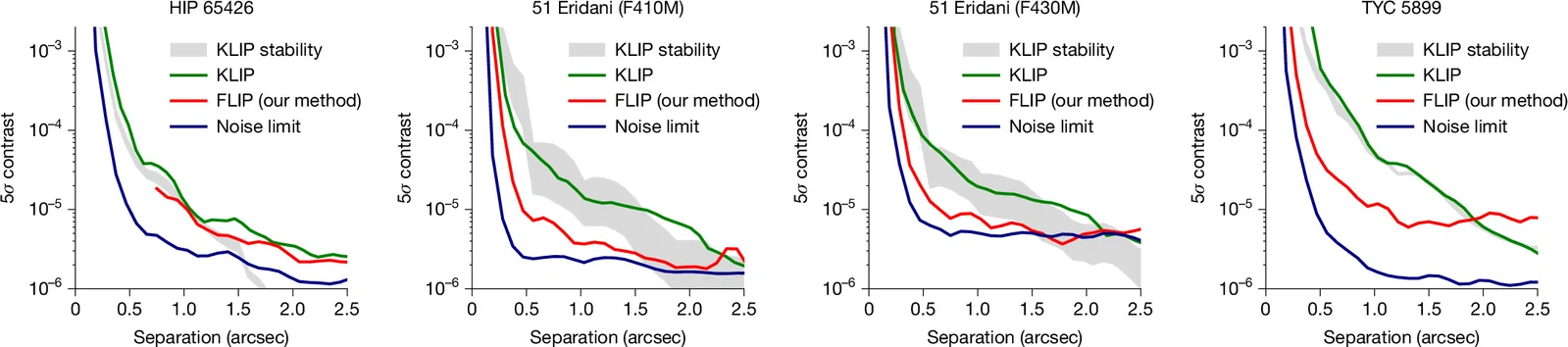
Detection sensitivity curves for KLIP and FLIP on each on-sky dataset. These curves are calibrated with injection-recovery tests. Also shown is the fundamental noise-limited sensitivity for these on-sky datasets and a range of estimated stability-limited KLIP sensitivity computed using simulated data. Our method enables substantial improvements (in some cases, 4–15 times) in exoplanet detection sensitivity. For the stability limit, we simulate 50 random realizations of reference and science observations with realistic OPD drifts and star–coronagraph misalignment and we run the same KLIP on these data (shaded regions correspond to the central 68% confidence interval from these 50 random draws). For HIP 65426, we do not sample the FLIP sensitivity within 0.6 arcsec owing to contamination from the bright planet PSF lobes, whereas we do sample the KLIP reductions because they are computed after subtracting the planet with KLIP-FM (ref. ^51^).

Figure 3 shows marked improvement in sensitivity to exoplanet detection compared with KLIP. For example, in TYC 5899, FLIP produces roughly 15 times better sensitivity than KLIP at 0.5 arcsec and approximately five times at 1 arcsec, and in the F430M image of 51 Eridani, FLIP provides about five times better sensitivity at 0.5 arcsec and about 2.5 times better at 1 arcsec. We also estimate a fiducial stability-limited sensitivity for KLIP, for which we quantify the predicted KLIP sensitivity limit assuming realistic differences between reference and science observations owing to the changing state of the observatory and assuming no other noise sources. We simulate observations with STPSF on similar observing conditions as the data, including realistic star–coronagraph misalignments^33^ and optical path difference (OPD) drifts, and accounting for algorithm over-subtraction intrinsic to KLIP. Although simplified, we show that the KLIP sensitivity on real data is consistent with our estimated stability-limited KLIP performance within a factor of two. In most cases, FLIP achieves performance well below this limit.

These results are illustrative of the potential of FLIP to aid exoplanet discovery and characterization with the JWST. Our new SNR = 6.3 detection of 51 Eridani b in F430M is the first image of the planet at 4.3 μm, in which the absorption band of carbon dioxide is located. This is a wavelength range inaccessible to ground-based measurements owing to telluric contamination from the Earth’s atmosphere. With CO_2_ being a good tracer of atmospheric metallicity, future work will use this detection to measure the 4.3-μm photometry of the planet and carry out a new atmospheric characterization. This will help assess previous evidence for an elevated metallicity of about five times the stellar value^26,35^ and better understand the formation history of 51 Eridani b. Similarly, our images of TYC 5899 show how FLIP can enhance exoplanet discovery, overcoming even sensitivity limitations from using reference stars observed months apart from the science observation. Several current exoplanet discovery surveys are similar to the TYC 5899 dataset, with self-referenced observing strategies. These use other science targets as reference stars to increase observing efficiency, which can include data taken across many weeks and months, as opposed to dedicated, ideal contemporaneous reference stars. This observation of TYC 5899 in particular was taken four months after the reference stars observations. Even in this case, FLIP yields sensitivity gains of up to an order of magnitude. With more than 100 observations taken in similar observing programmes (for example, JWST programmes GO 5835, SURVEY 6005), the exoplanet discovery potential of using FLIP in these surveys is large.

## Future improvements

By being insensitive to instrument instabilities in principle, model-based PSF subtraction could be capable of reaching sensitivities down to the fundamental noise limit^7^. FLIP, in general, achieves contrast levels much closer to the noise limit than standard method KLIP (Fig. 3), but the photon noise limit is not reached in any observation yet. Furthermore, although FLIP can effectively compensate for phase drifts and random star–coronagraph misalignments that limit KLIP, our models have not yet been systematically calibrated to retrieve accurate estimates of these instabilities with associated uncertainties. Clear avenues for improvement include a more sophisticated detector model to process deep observations of very bright stars^23,36^, disentangling degeneracies between different model parameters, a detailed exploration of algorithm hyperparameters^37^ reducing the computational cost of the method and a framework to handle large data volumes for ground-based applications. See Methods for details.

## Discussion

After more than 35 years of development in coronagraphic exoplanet imaging, this work is the first time to our knowledge that model-based PSF subtraction surpasses standard PSF subtraction techniques, with up to an order of magnitude improvement in sensitivity. In the short term, the demonstrated 2–15 times sensitivity improvement will be notable for exoplanet discovery and characterization with the JWST, enabling access to fainter, and thus colder, lower mass or older, giant planets in JWST NIRCam observations^38–40^. This will expand the imaged exoplanet population precisely in the region of parameter space in which the JWST is most valuable^41^. Furthermore, with the predicted discovery of thousands of wide-separation giant planets from the Gaia mission^42,43^, FLIP could be instrumental to characterizing some of these planets with the JWST. Furthermore, our framework can be extended to the JWST Mid-Infrared Instrument (MIRI), contributing to exoplanet detection and characterization in valuable wavelengths beyond 5 μm.

In the long term, these first results show the potential of physics-based image processing on other current and future telescopes, as well as the crucial role that machine-learning-based techniques can play. For ground-based telescopes, recent work^24^ has shown great promise in non-coronagraphic PSF calibration. FLIP could be extended to coronagraphic ground-based imaging aided by detailed wavefront sensing and adaptive optics telemetry. Although this involves distinct data volume challenges (see Supplementary Discussion in the Supplementary Information), the potential to reach the photon noise limit could be extremely valuable (especially at shorter wavelengths at which we are typically limited by contrast rather than thermal background), particularly to directly image temperate terrestrial planets around low-mass stars with the extremely large telescopes^44,45^. Similarly for future space-based missions, the extreme levels of stability needed to image exo-Earths with the future HWO is a substantial challenge^46,47^, aggressively restricting the design space for the telescope. In this work, we have shown sensitivity beyond the stability limit in JWST NIRCam. If this can be extended to HWO contrast levels, the ultrastability requirement for the system may be partially lifted, with the error budget necessary for image post-processing being reduced, and thus the design of the observatory may become more tractable. Investigating this through testing in the Nancy Grace Roman Space Telescope^48^ and the Lazuli Space Telescope^49^ will be critical. If successful, the goal of imaging true exo-Earths and achieving photon-noise-limited high-contrast imaging for the first time will be closer to reality.

## Methods

### The FLIP framework

#### Model design

**Extended Data Fig. 1:**
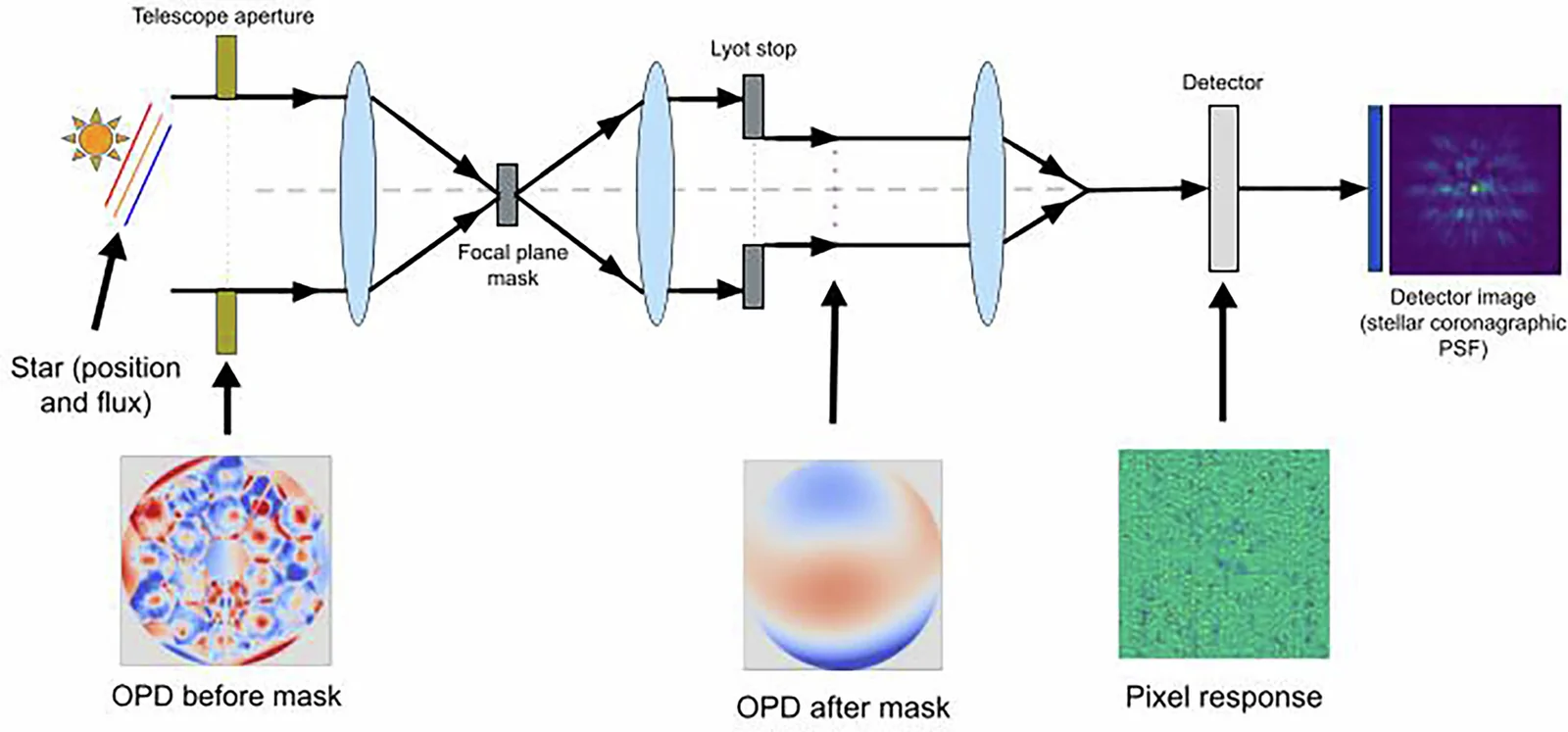
Schematic of the JWST coronagraph optical model used in this work. We highlight the parameters we tune in our model.

FLIP models the stellar PSF by optimizing many of the free parameters in a physical model that influence the PSF, including the star being observed and the instrument used for the observation. We base our models on the nominal STPSF optical models, which are used to simulate PSFs in the JWST for a variety of instruments and filters. Extended Data Fig. 1 shows a schematic diagram equivalent to the physical models used to simulate observations with the JWST NIRCam coronagraphs.

Given these models, we find that four groups of free parameters are sufficient for PSF fidelity in our experiments:

- The flux of the star and its precise position behind the focal plane mask of the coronagraph.
- The OPD (a phase aberration) before the focal plane mask of the coronagraph.
- The OPD after the focal plane mask of the coronagraph.
- The response per pixel in the detector.

Physically, these parameters capture the imperfect alignment of the star with the coronagraph (telescope pointing errors), the imperfect alignment of the primary mirrors in the JWST (deformations or tilts relative to the closest-in-time OPD measurement), the imperfectly known OPD internal to specific instruments in the JWST (in our case, the coronagraphs in the NIRCam instrument) and imperfectly known detector calibrations. Although other physical mechanisms are capable of affecting the PSF (such as pupil shear, manufacturing errors in the optics or more complicated detector physics), we find that our choice of free parameters is generally capable of effectively reproducing the effects of these other systematics and produce high-quality PSF subtractions. A summary of our model parameters is shown in Extended Data Table 1.

*Implementation details*. Following STPSF, our model implements light propagation in the Fraunhofer approximation through Fourier optics. When modelling any specific observation, we use STPSF to calculate the nominal instrument state and produce a simulated PSF, taking into account the instrument and filter, the closest-in-time OPD measurement to the reference star observation and the spectrum of the host star. We take this instrument state and replicate it in PyTorch^28^. See ref. ^27^ for details on the STPSF model construction and Supplementary Methods in the Supplementary Information for details on our implementation.

#### Optimization design

Previous work on simulated monochromatic data^25^ found that, given a near perfectly accurate physical model, it is possible to optimize it directly on science observations and produce excellent PSF subtraction without overfitting planet signals. However, in our experiments with real data, we find that accidentally subtracting planet signals is indeed possible. This is probably because of STPSF models having substantial inaccuracies relative to real JWST instruments and to our model being much more flexible with more free parameters, thus making the optimization problem less straightforward.

To address this, we implement a two-stage optimization. In stage 1, we use the full flexibility of our model and calibrate it on a reference star (because, by design, it is unlikely to host any contaminants such as planets or binaries), optimizing all of the free parameters described in the ‘Model design’ section. This helps overcome inaccuracies in the optical model and sets the parameters closer to a good solution. In stage 2, we use our calibrated model and optimize only a reduced number of free parameters directly on the science observation. This accounts for different pointing errors and different OPDs between reference and science observations owing to telescope instabilities.

**Extended Data Fig. 2:**
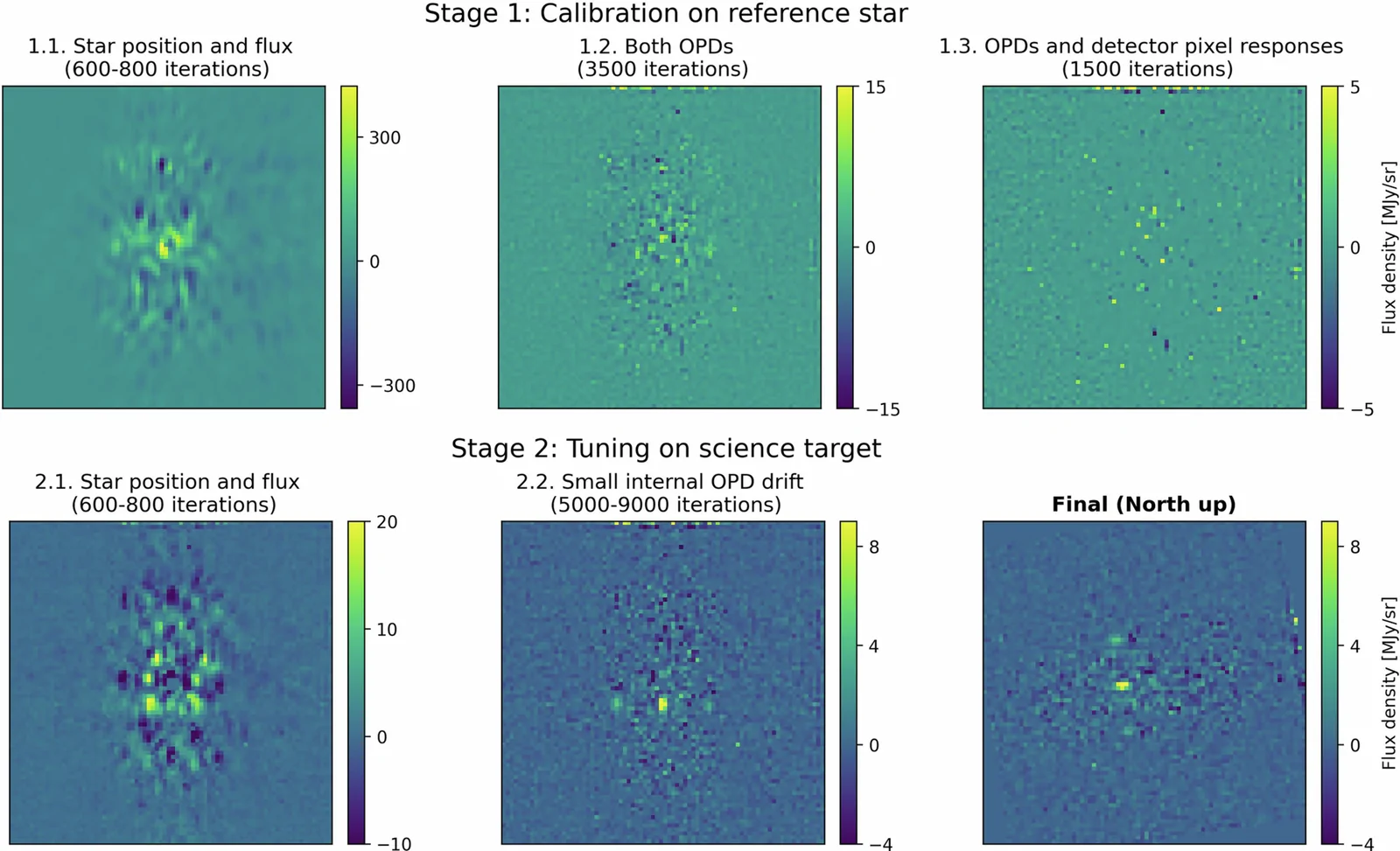
Summary of optimization stages in FLIP. This includes calibration (stage 1, top panels) on a reference star and parameter tuning (stage 2, bottom panels) on the science target of interest. The colour scale is adjusted in every step to show the dominant features of the residuals. The example shown is 51 Eridani in the F410M filter, revealing 51 Eridani b in the last panel. For visualization purposes, we keep all panels in the detector orientation, except for the last one, in which we rotate it north up as in Fig. 2. On a single NVIDIA A30 GPU, stage 1 takes 10–15 min and stage 2 takes 10–30 min.

In both stages, we optimize different parameters in separate sub-stages. This is done to reduce degeneracies between different parameters. In stage 1, we have three sub-stages: (1) solving for star position and flux; (2) solving for both OPDs; and (3) solving for both OPDs and pixel responses. In stage 2, we have two sub-stages: (1) solving for star position and flux and (2) solving for the OPD after the focal plane mask. Although the OPD before the coronagraph mask is also expected to change owing to the JWST mirrors, we at present find the optimization to be more stable if we solve for the OPD after the mask instead (as this OPD does not need to be propagated through the coronagraph mask). These stages are summarized and illustrated in Extended Data Fig. 2, with the number of iterations dedicated for each one. On a single NVIDIA A30 GPU, stage 1 takes 10–15 min and stage 2 takes 10–30 min.

**Extended Data Fig. 3:**
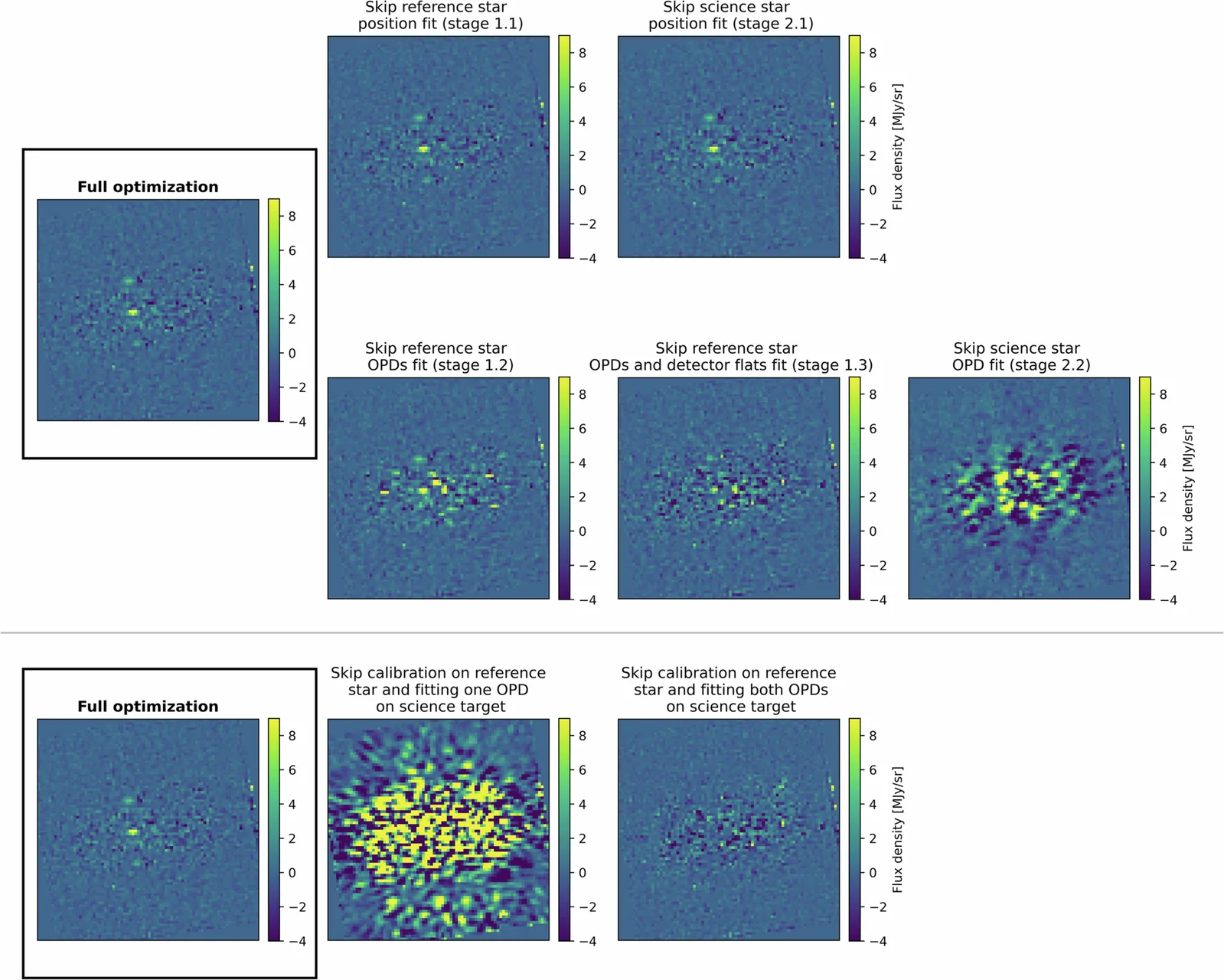
‘Leave one out’ tests on different stages of our optimization framework. Our full optimization is shown in the far left on 51 Eridani (F410M) for comparison. Top two rows, excluding individual stages. We skip the fit for star-mask misalignment (top) and the fits for individual OPDs and for detector effects (bottom). In all cases, we fit the flux of the star. Bottom row, excluding the calibration stage on the reference star as a whole. We show the result fitting only for the post-coronagraph OPD (middle) and for both OPDs (right) on the science target.

We carry out ‘leave one out’ tests to illustrate intuitively the reasons behind our contrast improvement (Extended Data Fig. 3). In the top two rows of Extended Data Fig. 3, we see that the star position has little effect on the contrast: although it is physically important (hence our inclusion), the effect is probably absorbed by other OPD terms in the model as tip-tilt phase aberrations. It is clear that OPDs strongly affect the PSF, as they are related to it directly by means of a Fourier transform. Detector responses are required to remove pixel-level errors that are not the result of and cannot be fit by OPDs. This illustrates the source of the improvements relative to approaches such as KLIP, as methods based on empirical reference images cannot account for arbitrary differences between reference and science data (stages 2.1 and 2.2). The bottom row of Extended Data Fig. 3 shows that model calibration is crucial, because removing it substantially increases the risk of planet over-subtraction. In the bottom row of Extended Data Fig. 3, we also see that a single OPD plane is not enough model flexibility: in our formulation, the interaction between both OPD planes (separated by a focal plane mask) is important. This illustrates the improvements of FLIP relative to previous autodiff-related efforts, notably ref. ^25^ (which used a far less flexible model and no model calibration).

*Implementation details*. We use the Adam optimizer^29^ with a cosine annealing learning rate scheduler. We use an L1 loss function for our optimization.

**Extended Data Fig. 4:**
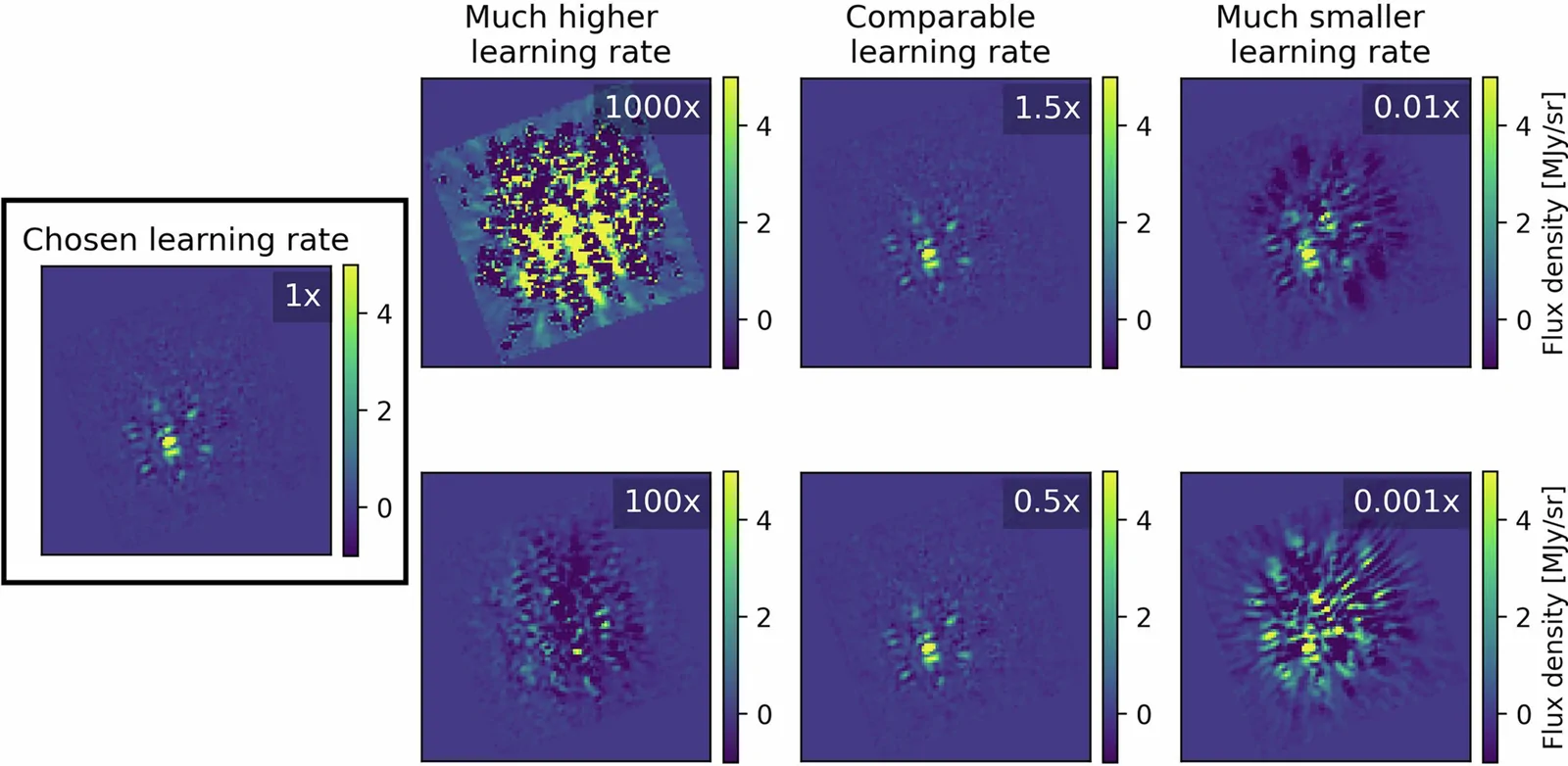
Sensitivity to chosen learning rates. Example FLIP runs using different learning rates while keeping the number of iterations identical. Increasing our learning rate by orders of magnitude leads to unstable optimization unable to converge (top left) or to substantial overfitting (bottom left). Lowering our learning rate by orders of magnitude increases the amount of unsubtracted starlight (bottom right) or, for this specific target, reduces the ability of our model to compensate for imperfect stellar flux estimate (stage 2.1) owing to the presence of the bright companion (top right). In each panel, all learning rates in the model are scaled by the factor indicated inside the panel.

We select the learning rates in our model by hand, increasing it across orders of magnitude until we find one that produces a stable optimization with convergence below around 15,000 iterations. Owing to the different magnitudes of different parameters in our model, we assign a different learning rate to each family of parameters. A simple coarse search clearly shows the appropriate scale for each parameter. Extended Data Fig. 4 shows that order-unity variations to learning rates do not substantially affect our results and that increasing or decreasing them by orders of magnitude clearly shows the correct scale for stability and relatively fast convergence. A summary of the order of magnitude of these learning rates per parameter is shown in Extended Data Table 1.

Across different observations, we find that globally scaling all learning rates by some order-unity constant can help with stability and convergence speed. This is probably because of the magnitude of the loss function depending on the brightness of the stars. We find that a global scaling of four works well in all of the experiments presented here, but preliminary analysis on fainter stars showed better results by using 1 or 0.5 instead in those cases.

Similarly, during the last sub-stage of our optimization, we reduce the global scaling factor to around 1% of its original value to further reduce risk of overfitting. Our results are not strongly sensitive to the exact choice of this value but it can be tuned differently for different observations depending on the similarity between reference star and science target.

**Extended Data Fig. 5:**
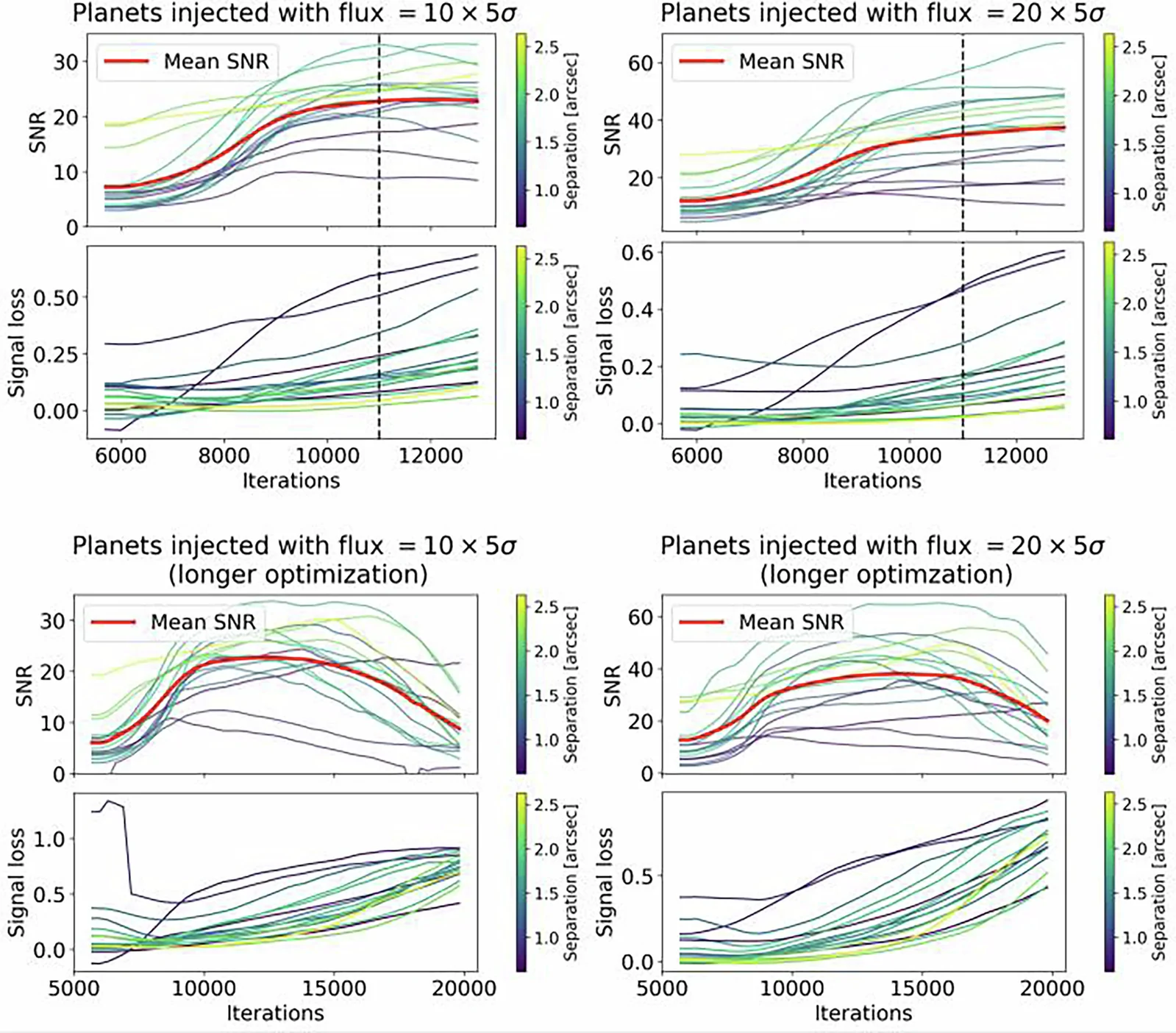
SNR ratio and signal loss for 16 example fake planets injected in the HIP 65426 dataset. In the left and right panels, all planets were injected at 10 and 20 times the 5*σ* fundamental noise contrast curve (blue curve in the HIP 65426 panel of Fig. 3) at the separation of the injected planet, respectively. The separation of each planet corresponds to the colour of each line. The top panels correspond to the number of iterations used in our experiments, with the stopping condition marked with a dashed line (11,000 iterations) and including the SNR and signal loss for 2,000 iterations beyond our stopping condition. Planets injected at most separations reach SNR plateaus at similar numbers of iterations, with no strong dependence on the brightness of the injection (left and right panels). The bottom panels show the optimization resulting from substantially increasing the number of iterations, for which we set our learning rate scheduler to have 20,000 total iterations.

To determine a stopping point for our optimization, we inject fake planets in the science observation and track their SNR as a function of iteration number. When measuring sensitivity, we inject planets at 5–7 separations and 1–3 planets per separation. For each injection, we inject the fake planet by simulating an off-axis PSF with the nominal STPSF model at the desired location in the field of view and adding it to the science image. We inject them at 4–20 times the 5*σ* fundamental noise contrast limit. This number may be adjusted depending on the amount of signal loss observed. We inject each planet individually per FLIP run. We measure the SNR every approximately 300 iterations. As expected, the SNR of each injected planet increases for several iterations and then decreases owing to algorithm overfitting. Therefore we stop our optimization at the approximate iteration when the injected planets reach an SNR plateau or peak on average, which was found to be consistent for planets injected at similar separations. Extended Data Fig. 5 shows this for fake planets injected at a variety of separations. In light of this, in our experiments, for simplicity, we choose our stopping point tracking a single fake planet injection. Just as with KLIP, the optimal fake planet injection strategy depends on context. For instance, we can choose as the stopping point the iteration number with maximum average SNR across all planet injections (for example, if the goal is to maximize sensitivity across the field of view) or based on one or several injections at a specific brightness and position (for example, if the goal is to optimize the SNR of a specific planet signal). Extended Data Fig. 5 also shows that our PSF subtraction is not strongly sensitive to our specific choices of fake planet injections.

### Experiments

We carried out experiments on four datasets: three with known companions (HIP 65426 in the F444W filter and 51 Eridani in the F410M and F430M filters) and one with no companions, part of a planet search survey (TYC 5899) in the F444W filter. HIP 65426 and TYC 5899 are observed with the MASK335R coronagraph mask, whereas 51 Eridani is observed with the LWBAR coronagraph mask in the NARROW position offset. We selected this diverse set of data to be representative of a wide range of observations with JWST NIRCam coronagraphy. In particular, our four datasets include:

1. Three different physical systems (HIP 65426, 51 Eridani and TYC 5899).
2. Three different observational filters (F444W, F410M and F430M).
3. Two different coronagraphs (NIRCam 335R round coronagraph and NIRCam LWBAR bar coronagraph).
4. Both real planets (HIP 65416 b and 51 Eridani b) and simulated planets (TYC 5899).
5. Observations with a dedicated reference star with a 9-POINT-CIRCLE dither pattern (HIP 65426), with a dedicated reference star with 5-POINT-BAR dither pattern (51 Eridani) and without dedicated reference stars but with a PSF library composed from a self-referenced survey (TYC 5899). This is relevant for the KLIP performance under a variety of reference star configurations.
6. Observations with reference stars close in time to the science target (HIP 65426 and 51 Eridani) and far in time from the science target (TYC 5899).
7. Observations of host stars of various spectral types: A2V (HIP 65426), F0IV (51 Eridani) and M3 (TYC 5899).

We run standard PSF subtraction method KLIP and our technique FLIP on all four datasets and compare the performance of each, both with the SNR of the known recovered companions and with the planet detection sensitivity at several separations from the star. We perform our KLIP subtractions in accordance with the optimal set of KLIP hyperparameters described in the respective publications to replicate what the authors considered the best performance. In particular, all KLIP subtractions are done using RDI + ADI (see, for example, ref. ^31^) on both roll angle observations of the science targets, which we then de-rotate and mean-combine. In all cases, we obtain PSF subtractions comparable with the published works. For FLIP, for simplicity, we perform it on only one roll per dataset. Future work could explore the differences in sensitivity between using FLIP only on one roll, FLIP on both rolls and mean-combining the de-rotated images and FLIP on both rolls, using ADI on the resulting images and mean-combining the FLIP + ADI results.

For TYC 5899, the original publication^32^ presents two epochs: one with a single roll angle (the second failed) and one with two roll angles observed four months after the reference star sequences. We use the latter in this analysis for both KLIP and FLIP, to illustrate the gains in sensitivity when there is substantial drift between reference and science observations. Although ref. ^32^ used all three observations for their contrast curves and we replicate it only with the last two, we obtain comparable contrast performance: about 3 × 10^−6^ at 2.5 arcsec and about 4 × 10^−5^ at 1.0 arcsec for KLIP and FLIP and about 3 × 10^−4^ for KLIP and about 5 × 10^−4^ for FLIP at 0.5 arcsec. Because the target has no known companions, we inject planets at various separations and brightnesses to compare the performance of KLIP and FLIP in a controlled setting.

Extended Data Table 2 shows the optimization hyperparameters used during our reductions with FLIP.

#### Data preparation

The observations of HIP 65426, 51 Eridani and TYC 5899 and their reference stars were processed with the JWST pipeline (version 1.18.1) and the community-developed tool spaceKLIP (version 2.2.1). Data processing steps (which include ramp-fitting, converting from detector counts to physical flux units and bad pixel cleaning) and observation set-up are detailed in the respective papers for those observations^26,31,32^.

The JWST and spaceKLIP pipelines include bad pixel flagging and cleaning. However, imperfect bad pixel cleaning can cause more difficulties in our model-based PSF subtraction than in methods such as KLIP. This is because hot or dead pixels, or variations in pixel responses, cannot be ‘cancelled out’ by subtracting observations with the same pixel variations. Therefore, bad pixels that persist even after the automated cleaning routines are identified by eye and masked during our optimization process.

#### Choice of reference stars

Both HIP 65426 and 51 Eridani were observations carried out with dedicated reference stars: a bright star with probably no planets around it, of similar spectral type to the science target, observed just before or after the science observation. For these targets, we use as our calibration star (optimization stage 1; Extended Data Fig. 2) the same reference stars used for those observations: HIP 68245 for HIP 65426 and HD 30562 for 51 Eridani. We use a single dither for model calibration with FLIP for simplicity but all dithers are used to build the principal components for KLIP. See refs. ^26,31^ for details.

For TYC 5899, the observation was taken as part of a self-referenced programme, in which only science observations are taken, and a large number of these are put together to be used as a reference library for every other science observation. For our KLIP subtractions, we use the full PSF library used in ref. ^32^ to reproduce the result in their published work. For an apples-to-apples comparison of FLIP and KLIP, we chose one of the observations in the self-referenced library as our calibration star for stage 1 of FLIP. We select the HIP 17695 observation as it has the most similar spectral type to TYC 5899 from the targets in the library. See ref. ^32^ for details.

### PSF subtraction performance metrics

The contrast curves for both KLIP and FLIP were calculated by dividing the image into small concentric annuli and computing the standard deviation within each annulus accounting for small sample statistics^50^ and for planet signal loss owing to both algorithm overfitting and coronagraph throughput. For reference, the signal loss from algorithm overfitting near the location of the measured planets (in the same order as in Fig. 2) for KLIP is about 5–10%, about 20–70%, about 50–80% and about 10–40% and for FLIP it is about 5–20%, about 20–40%, about 30–50% and about 40–60%. We also smooth all datasets with a Gaussian kernel (*σ* = 1 pixel) before computing the standard deviations, as FLIP residuals are mostly dominated by pixel-to-pixel noise. For HIP 65426, the FLIP contrast curve excludes the central 0.6 arcsec of the image given that a bright lobe of the planet PSF prevents sampling this region, whereas KLIP uses the KLIP-FM algorithm to subtract the planet before computing sensitivity. Future work incorporating planet modelling and subtraction with FLIP will overcome this limitation. See refs. ^26,31,32^ for details on computing and calibrating these contrast curves.

For the fundamental sensitivity curve, we use the ERR extensions of the JWST data products, which track the combined total uncertainty in each pixel value from read noise, Poisson noise and flat noise. We generate a random Gaussian sample with mean 0 and *σ* equal to the total noise level in each pixel. That is, when generating our noise realization, we are simulating what the image would look like after perfect PSF subtraction. We finally compute the contrast curve on this image in the same way as described above for KLIP and FLIP subtractions, assuming no planet signal loss. This assumes that the only source of fundamental noise comes from the science observation. This is essentially the case 0 presented in ref. ^4^. In KLIP, further noise also comes from the reference observations (see ref. ^4^). FLIP is model-based PSF subtraction, so our FLIP subtraction adds no extra fundamental noise. The fundamental noise curves can also be interpreted as a lower limit, or the most optimistic case, of the possible sensitivity. Because we perform FLIP on only one roll per dataset, the noise limit corresponds to the noise limit of that roll.

**Extended Data Fig. 6:**
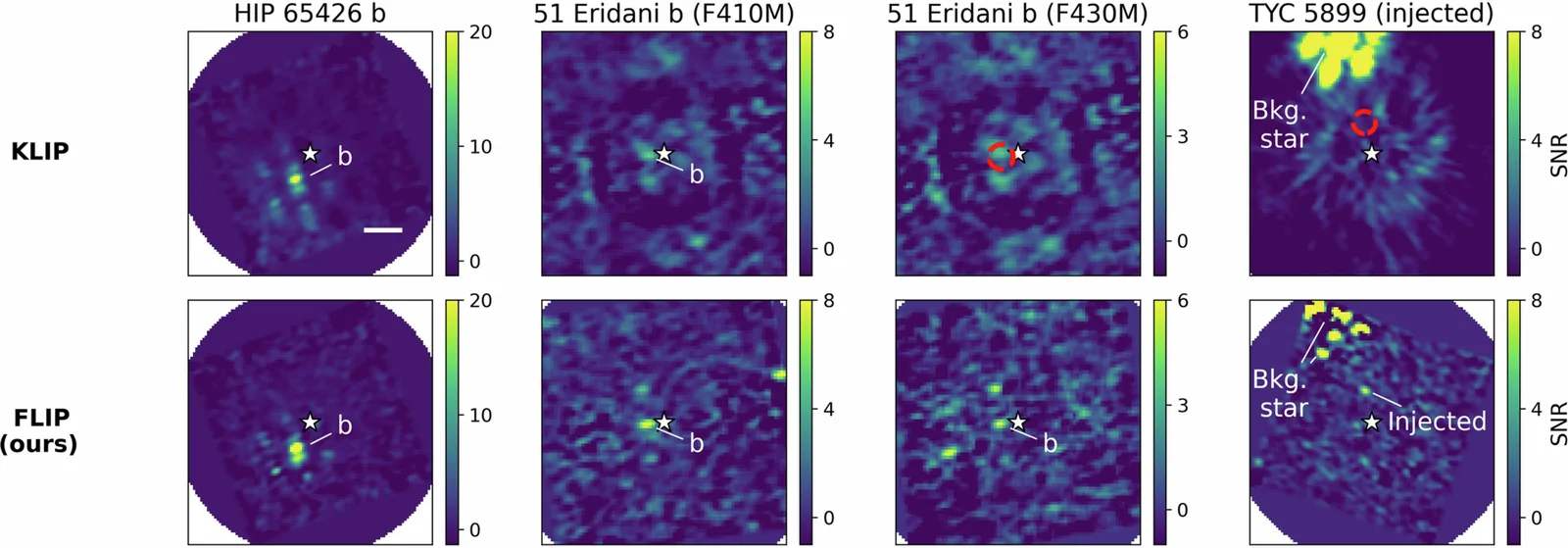
SNR maps for Fig. 2. In FLIP, in all cases, the planet signal corresponds to the highest SNR in the scene (other than the background star for the TYC 5899 dataset). The F410M image of 51 Eridani shows an artefact on the right edge of the field of view (visible also in Fig. 2). The F430M image of 51 Eridani also exhibits a signal to the left of the planet (visible also as a source in Fig. 2 flux maps, much fainter than the real companion); the nature of this signal is inconclusive.

To supplement Fig. 2, we compute SNR maps for each PSF-subtracted image (Extended Data Fig. 6). We generate a non-coronagraphic PSF model cropped to the central 5 × 5 pixels and cross-correlate our image with the PSF model. Then we compute the SNR of each individual pixel within an annulus of width approximately 0.2 arcsec in a two-step process: we compute the noise per annulus to identify pixels with SNR > 5 as a first pass and then compute a refined noise estimate excluding those pixels. The final SNR map is simply the pixel values of the cross-correlation map divided by the refined noise estimate.

### Estimation of the stability-limited sensitivity

In Fig. 3, we show a fiducial stability-limited sensitivity. This limit is computed by performing KLIP on simulated data, in which we can isolate the physical factors that contribute to the KLIP performance limitations. We perform 50 realizations of each observation sequence, stochastically varying the star–coronagraph misalignment and the OPD drift consistent with empirical JWST characterization in flight^5,33^, as detailed below. To isolate the effects of these instabilities, we include no other source of noise (for example, photon noise, flat noise) in our simulations.

For the science target, we generate STPSF models using the spectral type of the star and scale the flux within the central 20-pixel radius to the flux of the real data. We simulate two rolls offset by 10° in position angle. For the reference stars, for HIP 65426, we simulate nine PSFs following the 9-POINT-CIRCLE dither strategy, for 51 Eridani five PSFs following the 5-POINT-BAR dither strategy (https://jwst-docs.stsci.edu/jwst-near-infrared-camera/nircam-operations/nircam-dithers-and-mosaics/nircam-subpixel-dithers/nircam-small-grid-dithers#gsc.tab=0) and for TYC 5899, we simulate the same number of reference stars that were used in the PSF library of ref. ^32^.

We assume that all reference stars are of identical spectral type and brightness to the science target and assume no photon, detector or flat noise. In some targets, this results in the KLIP stability limit being deeper than the fundamental noise limit at large separations from the star (Fig. 3). This is acceptable for our purposes, because at these close separations, we are generally limited by PSF subtraction inaccuracies rather than fundamental noise and we are mostly interested in improving sensitivity in the regions of the image in which we are stability-limited rather than fundamental-noise-limited.

For each observation, we simulate a target acquisition error with a random star–coronagraph misalignment drawn from ${\mathcal{N}}(\mu =0,$ $\sigma =10)\,{\rm{mas}}$, consistent with target acquisition accuracy in flight^33^. We simulate the PSFs with the closest-in-time OPD measurement. We assume that there is no notable OPD drift between reference star dithers. To mimic small OPD drifts owing to telescope slews, for each observation in the real data that required a telescope slew (for example, going from reference star to the science target), we add an OPD drift term to the OPD measurement. The shape of the OPD drift is taken from subtracting the two closest-in-time OPD measurements to the observation being simulated. The root mean square (r.m.s., that is, the amplitude) of the drift term is randomly drawn from ${\mathcal{N}}(\mu =4,\sigma =1)\,{\rm{nm}}$, smaller than drifts measured over day timescales (about 7–9 nm r.m.s.) and bigger than drifts on minutes timescales (about 1–2 nm r.m.s.)^5^.

For HIP 65426, the bright companion has a non-negligible effect on PSF subtraction by skewing the KLIP algorithm (for example, during mean subtraction and in the calculation of Karhunen–Loève (KL) modes). We recreate this effect by injecting a fake planet in our HIP 65426 observation at the same contrast, separation and position angle as the real companion in our simulated observation.

We perform KLIP with ADI + RDI on all targets, using all KL modes for HIP 65426 and 51 Eridani and six KL modes for TYC 5899, following the respective publications. We compute our calibrated stability-limited KLIP sensitivity with injection-recovery tests, injecting planets at 3–7 separations and 2–3 position angles across the 80 × 80-pixel field of view, with the injected flux being four times the uncalibrated KLIP sensitivity at the injection separation. The central 68% range of the resulting contrast curves are plotted as shaded regions in Fig. 3.

### TYC 5899 synthetic injections

**Extended Data Fig. 7:**
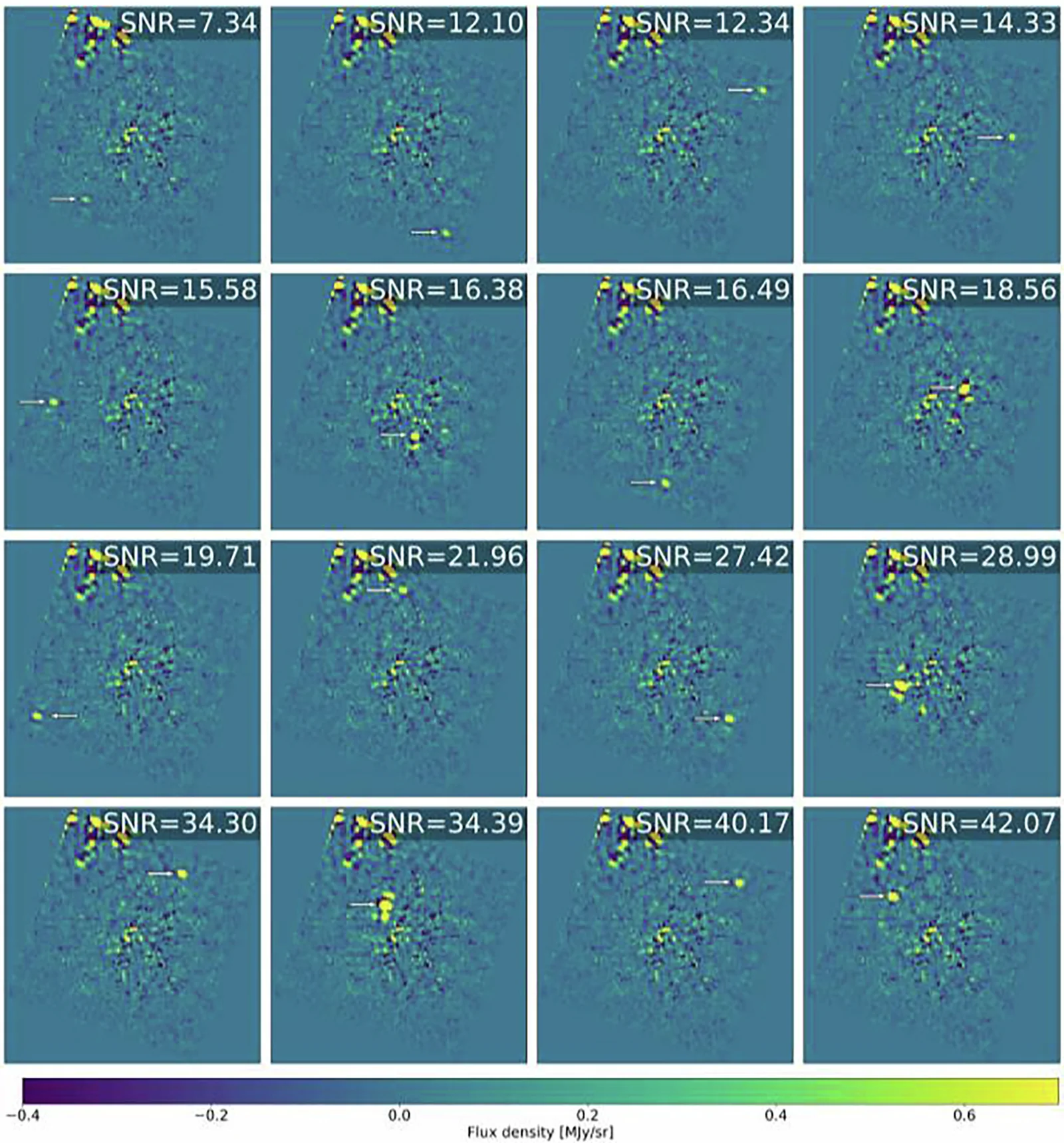
Suite of injection and recovery tests with FLIP. We perform these on the TYC 5899 dataset. The location of the synthetic planet is annotated with an arrow. The nearby off-axis bright star is also visible in the upper left.

**Extended Data Fig. 8:**
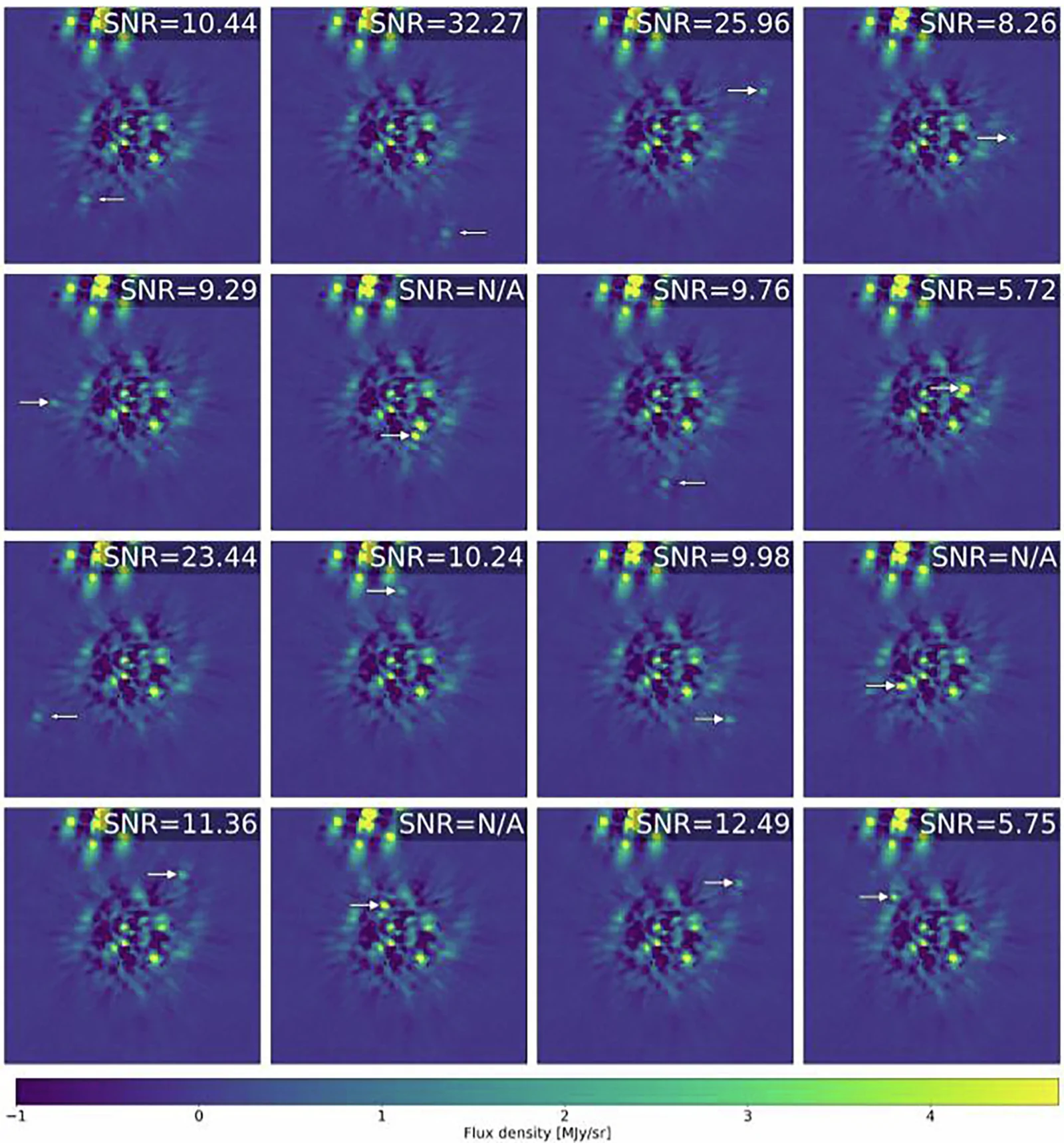
Suite of injection and recovery tests with KLIP. Same as Fig. 7 but using KLIP for PSF subtraction. All injections that resulted in SNR lower than 5 are labelled as SNR = N/A.

To compare the sensitivity of FLIP and KLIP on TYC 5899, we inject fake planets with known flux across the image and assess the recovery PSF subtraction. Extended Data Figs. 7 and 8 show a range of planet injections, complementing Fig. 2. At each separation, we inject a planet with flux equal to 20 times the 5*σ* fundamental noise limit. For PSF subtraction, we process all observations in Extended Data Figs. 7 and 8 identically, using the same learning rates and numbers of iterations for FLIP and the same numbers of annuli and KL modes for KLIP. This is a standard method to quantify sensitivity in KLIP (for example, ref. ^37^): once a set of annuli and KL modes have been chosen (for example, to recover the signal in Fig. 2), we inject fake planets across the field of view while keeping the annuli and KL modes fixed. This estimates the detection sensitivity across the field of view, given a set of hyperparameters. In FLIP, we use an analogous strategy, by injecting fake planets across the field of view, while keeping the learning rates and numbers of iterations fixed.

We find that FLIP outperforms the standard method KLIP in most cases, especially at closer separations, producing higher SNR on a diverse range of possible planets. We also see that, in this particular dataset, at larger separations, FLIP produces higher signal loss than KLIP in some cases, which explains the contrast curve for KLIP being deeper than FLIP at larger separations in Fig. 3. Future work could address these differences in signal loss. Nevertheless, at these larger separations, we are often background-limited rather than contrast-limited; that is, sensitivity is mainly limited by exposure time rather than by starlight subtraction. In the interesting regime in which we are limited by telescope stability and imperfect starlight subtraction, at smaller angular separations (which also correspond to more Solar-System-like physical separations), FLIP robustly outperforms KLIP.

## Online content

Any methods, additional references, Nature Portfolio reporting summaries, source data, extended data, supplementary information, acknowledgements, peer review information; details of author contributions and competing interests; and statements of data and code availability are available at https://doi.org/10.1038/s41586-026-11213-z.

## Supplementary information

Supplementary Information This file contains supplementary methods and supplementary references

Peer Review File

## Figures and Tables

**Extended Data Table 1:**
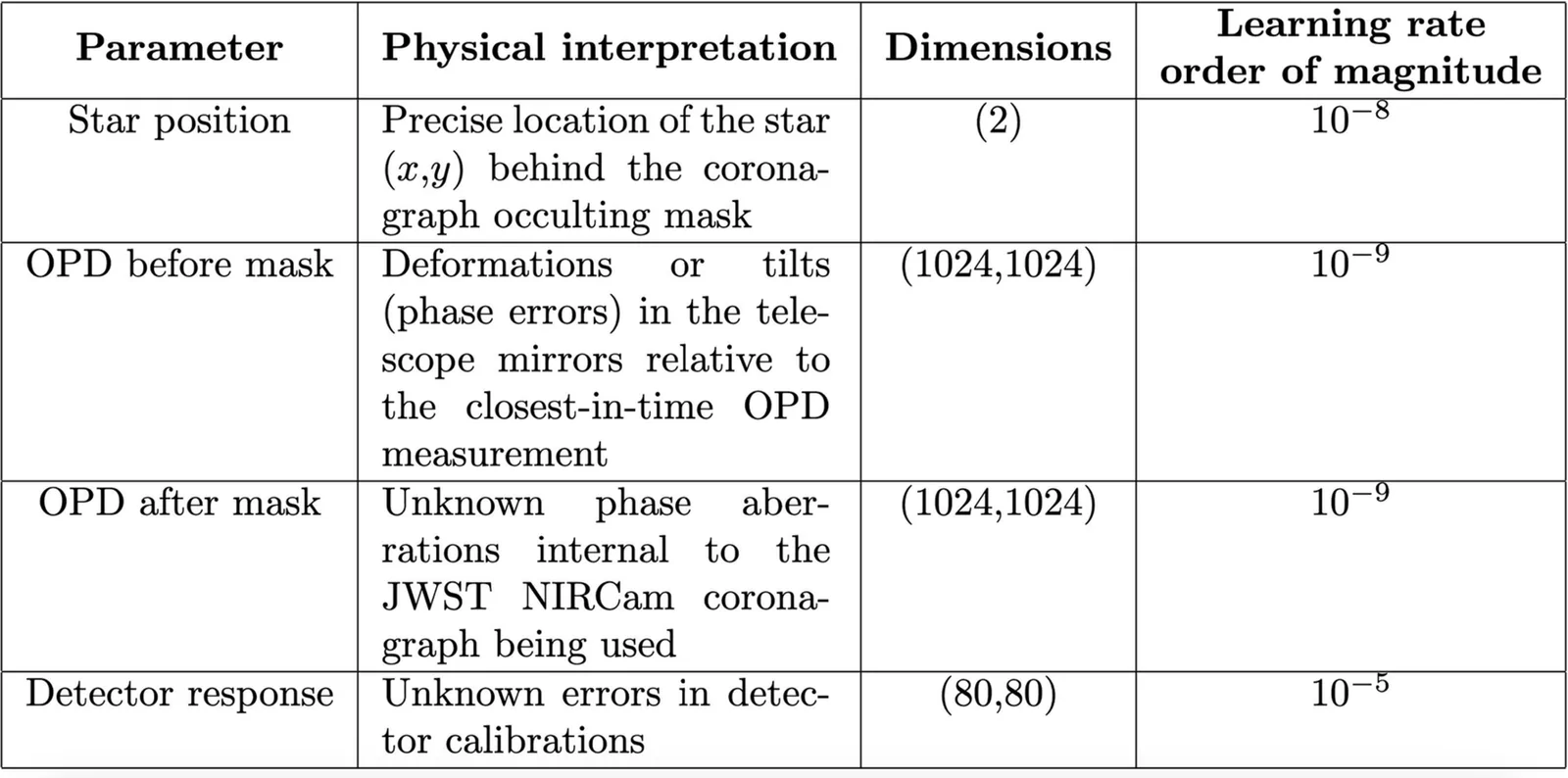
Summary of free parameters used in our model

We show their physical meaning and characteristic learning rates during our optimization. The dimensionality of the detector response parameters depends on the field of view being optimized over (in our case, 80 × 80 pixels in all cases). The flux of the star is scaled automatically to match the total flux across the image during the first sub-stage of stages 1 and 2. As mentioned in the text, although other physical mechanisms can influence the stellar PSF, we find that these four groups of parameters are sufficient to model the PSF to the contrast levels shown in Fig. 3. For this reason, in this work, the inferred parameters do not necessarily reflect the true state of the instrument, rather, they produce an effective model that is flexible enough to reproduce a variety of systematics. See Supplementary Discussion in the Supplementary Information.

**Extended Data Table 2:**
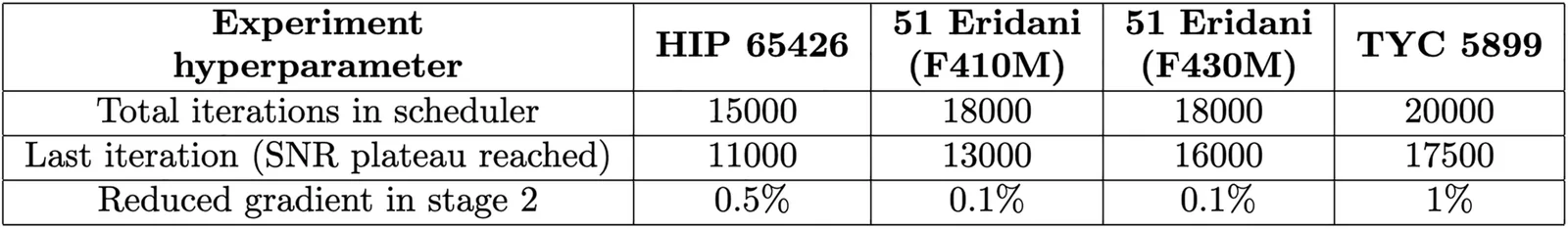
FLIP hyperparameters for each experiment

The reduced gradient is the result of scaling-down the loss by the factor shown.

## Data Availability

The JWST NIRCam data used in this paper are from JWST programmes ERS 1386 (PI: S. Hinkley), GTO 1412 (PI: M. Perrin) and GTO 1184 (PI: J. Schlieder) and are publicly available from the Mikulski Archive for Space Telescopes (MAST).

## Code availability

The FLIP code and an example demo with test data are available at https://github.com/rferrerc/pyflip/tree/submission (submission commit: a92e684).
