# Supplementary material for: Imaging exoplanets below the telescope stability limit
Source: Nature. 2026 Nov 4;659(8139):58–62. doi: 10.1038/s41586-026-11213-z (PMC13736631; doi:10.1038/s41586-026-11213-z)
Supplement: Supplementary file 1 [file 41586_2026_11213_MOESM1_ESM.pdf]

nature portfolio

https://doi.org/10.1038/s41586-026-11213-z

Supplementary information

Imaging exoplanets below the telescope stability limit

In the format provided by the authors and unedited

Imaging exoplanets below the telescope stability limit: Supplementary Information

Rodrigo Ferrer-Chavez[1,2,*] Jason J. Wang[1,2,†]
Katherine L. Bouman[3,†] William T. Freeman[4]
Laurent Pueyo[5] Brandon Y. Feng[4] Marshall D. Perrin[5]

[1]Department of Physics and Astronomy, Northwestern University, 2145 Sheridan Rd, Evanston, IL 60208, USA

[2]Center for Interdisciplinary Exploration and Research in Astrophysics (CIERA), Northwestern University, 1800 Sherman Ave, Evanston, IL 60201, USA

[3]Departments of Computing and Mathematical Sciences, Electrical Engineering, and Astronomy, California Institute of Technology, Pasadena, CA 91125 USA

[4] Computer Science and Artificial Intelligence Laboratory, Massachusetts Institute of Technology, Cambridge, MA 02139 USA

[5]Space Telescope Science Institute, 3700 San Martin Drive, Baltimore, MD 21218, USA

[*]Corresponding author.

[†]These authors contributed equally to this work.

1 Supplementary information

1.1 Supplementary Methods

Model design implementation details. Our physical model is initialized with the nominal optical system of STPSF, and we implement it in PyTorch to make it differentiable. We take the mask designs of the optical elements and the nominal wavelength and field-dependent phase aberrations.

On top of the nominal STPSF model, we perform a sub-pixel shift to align the center of the PSF (by default at the center of the output array) to the center of the coronagraph on the detector as measured for a given coronagraph mask and filter. We do this to perform the optimization directly on the detector coordinates, and avoid artifacts induced by interpolating and shifting the data itself. Also following STPSF, the PSF is convolved with a small Gaussian kernel ($\sigma =$ 0.018 arcseconds, value taken from STPSF[1]) to mimic a basic charge diffusion effect. Finally, each pixel is multiplied by a pixel response function.

We parametrize the OPDs as square arrays of 1024×1024 pixels, each pixel containing the OPD value in meters, in order to have a flexible OPD model.

Following recommendations from STPSF, we perform our simulations with pixel over-sampling of 2 in the image plane, and we use 21 monochromatic wavefronts for F444W observations and 9 for F410M and F430M for the best balance between numerical accuracy and evaluation speed.

Optimization design implementation details. The process of parameter tuning described (number of iterations, stopping condition) is analogous to tuning KLIP hyperparameters, such as choosing the number of annuli and KL modes to construct the PSF model. In KLIP, this involves finding the parameters that best reveal a particular astrophysical signal or injecting and recovering fake planets to find the best sensitivity, and verifying that a particular source

[1] https://stpsf.readthedocs.io/en/latest/jwst_detector_effects.html

is not strongly dependent on the number of KL modes used (e.g.,[1]). In FLIP, injecting fake planets and tracking their SNRs is analogous to this. In practice, we also verify that slightly tweaking these parameters does not significantly change an astrophysical source (e.g., Extended Data Figure 7 . In this work, we perform our optimization in the entire 80×80-pixel image. Additionally, while our optimization can be done on every integration separately, we found that optimizing on the time-averaged observations yielded better contrast results for much shorter computation time. This is probably because KLIP subtractions are currently limited by static or very slowly-evolving aberrations in JWST, as opposed to other shorter-timescale aberrations[2], so mean-combining provides a higher SNR observation, and higher SNR on the static components of the PSF in particular that need to be fit. On deep observations around bright stars, however, the time evolution of starlight residuals may be significant. Future work may explore optimizing on individual frames or series of frames, leveraging the temporal correlations in starlight residuals (e.g., adding regularization terms that impose similarity between frames or that formulate an explicit time evolution model).

PSF subtraction performance details. To compute the SNR of each planet detection in Figure 2, we smooth the images ($\sigma = 1$ px) and we fit a Gaussian to the core of the planet PSF at the known location of the signal. Next, we extract the peak flux of the Gaussian and divide it by the standard deviation of pixels within an annulus at the separation of the planet and with width of 0.2 arcseconds (except for HIP 65426, for which we use an annulus of width 0.5 arcseconds due to the large section of the image where the planet PSF is the dominant signal). SNR is converted to a confidence level using t-statistics[3], where we take the number of samples that determine the Student-t distribution to be the number of 3×3-pixel boxes (roughly the size of the

resolution element) within the annular region.

1.2 Supplementary Discussion

1.2.1 Future improvement details

While our experiments show significant improvements relative to the state-of-the-art technique KLIP on a variety of datasets, FLIP has not reached the fundamental noise limit yet. The findings in this work lead to the future avenues for improvement shown below:

Detector model: Our simplified detector model is sufficient to model the majority of observations taken by JWST NIRCam. However, observations of very bright stars or very deep observations require a more sophisticated treatment of the complex detector effects present in JWST[4]. This is because pixel-level defects or pipeline-induced artifacts are significantly above the noise limit and accurate modeling becomes essential. Sophisticated effective detector models or models of the data pre-processing pipeline (following[5]) are promising to push this technique to the very high contrast regime. Furthermore, careful treatment of detector systematics will likely be crucial to extend this framework to JWST MIRI[6].

Inferred instrument parameters: Coronagraphy is an ill-posed and degenerate problem[7,8], and as such there is a wide range of solutions that may produce effectively the same PSF estimate. Furthermore, the nominal `STPSF` models for JWST NIRCam, which we use to initialize our differentiable model, are known to be imperfect. To use FLIP to retrieve realistic instrument parameters with rigorously validated uncertainties future calibration work is required. This could include placing strong regularization priors on some of the model parameters (e.g.,[9]), jointly fitting a large number of observations or processing datasets with multiple stars in the field of view with multiple point source

models. Successful calibration will enable retrieving the state of the instrument during any science observations in a physically realistic way, contributing to more accurate understanding and modeling of JWST NIRCam.

Computational cost: For the datasets analyzed in this work, the runtime of FLIP on an NVIDIA A30 GPU varies from 15 to 35 minutes. This is very efficient for a model with 10^6 free parameters, but exoplanet imaging routinely requires multiple runs (e.g., to calibrate total sensitivity as in Figure 3). Addressing systematic model mismatch (improving our initial conditions) and implementing regularization on some parameters are two ways to potentially improve the speed of FLIP, perhaps close to the one minute runtime shown in monochromatic simulated data[10].

Scenes with bright astrophysical sources: For simplicity, the model presented here only models the stellar PSF of the host star. As seen in Figure 2, however, in some cases other astrophysical sources, such as bright off-axis stars, close binaries or debris disks are also present in the scenes. With no other constraints, our model ends up partially distorting these other objects. Future work on model calibration and parameter regularization could slightly reduce the flexibility of our model to avoid distorting other components in the scene. Alternatively, joint modeling of both the host star and these objects is promising for simultaneously minimizing signal distortion and characterizing the properties of these objects.

Applicability to ground-based observations: In a space-based instrument like JWST, the dominant source of instability for most datasets comes from drift between the reference star observation and the science observation. For this reason we obtain good results performing our optimization only once or only a handful of times per dataset (averaging all or a few subsets of the frames of the observation). However, atmospheric turbulence in ground-based

observations evolves on much faster timescales, and so does the stellar PSF.

While FLIP can be applied to ground-based observatories in principle, simply forward modeling a larger number of wavefront sensing measurements quickly becomes very computationally expensive: ground-based adaptive optics systems measure wavefront errors roughly every millisecond during every science observation, while JWST does it only once roughly every two days. Wavefront sensing data, however, should also significantly help constrain the problem, since they are taken simultaneously with the science observation, unlike JWST.

Future work addressing this data volume challenge (and associated sources of aberrations beyond atmospheric turbulence[11,12,13]) will be critical to extend this framework to ground-based telescopes. For example, empirical methods have been able to partially manage the high dimensionally of the problem using sparsity regularization (e.g.,[14]). Recent work by[15] has also explored non-coronagraphic PSF reconstruction by combining physical models with deep learning models and a small set of physical parameters related to observing conditions, without relying on full adaptive optics telemetry. While the high level of PSF fidelity required for high-contrast imaging will likely benefit from explicit telemetry, frameworks like the one presented in[15] could be a promising starting point, especially when full adaptive optics telemetry is not available. These and other approaches could be used in future iterations of FLIP to reduce the computational load on ground-based datasets.

Supplementary Information References

[1] Sanghi, A. et al. “Worlds Next Door: A Candidate Giant Planet Imaged in the Habitable Zone of α Centauri A. II. Binary Star Modeling, Planet and Exozodi Search, and Sensitivity Analysis”. In: The Astrophysical Journal Letters 989.2 (2025), p. L23.

[2] Telfer, R. C. et al. "Empirical characterization of JWST wavefront error variations". In: Space Telescopes and Instrumentation 2024: Optical, Infrared, and Millimeter Wave. Vol. 13092. SPIE. 2024, pp. 269–283.

[3] Mawet, D. et al. "Fundamental limitations of high contrast imaging set by small sample statistics". In: The Astrophysical Journal 792.2 (2014), p. 97.

[4] Goudfrooij, P., Grumm, D., Volk, K., and Bushouse, H. "An Algorithm to Mitigate Charge Migration Effects in Data from the Near Infrared Imager and Slitless Spectrograph on the James Webb Space Telescope". In: Publications of the Astronomical Society of the Pacific 136.1 (2024), p. 014503.

[5] Desdoigts, L. et al. "AMIGO: a data-driven calibration of the JWST interferometer". In: Publications of the Astronomical Society of Australia 43 (2026), e075.

[6] Argyriou, I. et al. "The brighter-fatter effect in the JWST MIRI Si: As IBC detectors-I. Observations, impact on science, and modeling". In: Astronomy & Astrophysics 680 (2023), A96.

[7] Xin, Y. et al. "Coronagraphic data post-processing using projections on instrumental modes". In: The Astrophysical Journal 963.2 (2024), p. 96.

[8] Pope, B. J., Pueyo, L., Xin, Y., and Tuthill, P. G. "Kernel phase and coronagraphy with automatic differentiation". In: The Astrophysical Journal 907.1 (2021), p. 40.

[9] Feng, B. T., Bouman, K. L., and Freeman, W. T. "Event-horizon-scale imaging of M87* under different assumptions via deep generative image priors". In: The Astrophysical Journal 975.2 (2024), p. 201.

[10] Feng, B. Y. et al. "Exoplanet Imaging via Differentiable Rendering". In: IEEE Transactions on Computational Imaging (2025).

[11] Cantalloube, F. et al. "Wind-driven halo in high-contrast images. I. Analysis of the focal-plane images of SPHERE". In: 638, A98 (June 2020), A98. DOI: 10.1051/0004-6361/201937397. arXiv: 2003.05794 [astro-ph.IM].

[12] Wang, L., Schoeck, M., Veran, J.-P., Vogiatzis, K., and Boyer, C. "Petalling mode sensing for the Thirty Meter Telescope". In: Adaptive Optics Systems IX. Ed. by K. J. Jackson, D. Schmidt, and E. Vernet. Vol. 13097. Society of Photo-Optical Instrumentation Engineers (SPIE) Conference Series. Aug. 2024, 130972F, 130972F. DOI: 10.1117/12.3019282.

[13] Levraud, N. et al. "Properties of petal mode and its measurement in an adaptive optic closed loop". In: Adaptive Optics Systems IX. Ed. by K. J. Jackson, D. Schmidt, and E. Vernet. Vol. 13097. Society of Photo-Optical Instrumentation Engineers (SPIE) Conference Series. Aug. 2024, 130972E, 130972E. DOI: 10.1117/12.3020124.

[14] Bonse, M. J. et al. "Use the 4S (Signal-Safe Speckle Subtraction): Explainable Machine Learning Reveals the Giant Exoplanet AF Lep b in High-contrast Imaging Data from 2011". In: The Astronomical Journal 169.4 (2025), p. 194.

[15] Kuznetsov, A., Neichel, B., Oberti, S., and Fusco, T. "Data-calibrated point spread function prediction: General description of the method and demonstration on MUSE-NFM". In: Astronomy & Astrophysics 708 (2026), A311.
