# Supplementary material for: Imaging exoplanets below the telescope stability limit
Source: Nature. 2026 Nov 4;659(8139):58–62. doi: 10.1038/s41586-026-11213-z (PMC13736631; doi:10.1038/s41586-026-11213-z)
Supplement: Supplementary file 2 [file 41586_2026_11213_MOESM2_ESM.pdf]

nature portfolio

Peer Review File

Imaging exoplanets below the telescope stability limit

Corresponding Author: Mr Rodrigo Ferrer-Chavez

Any redactions in this file are there to maintain patient confidentiality, the confidentiality of unpublished data, or to remove third-party material.

This file contains all reviewer reports in order by version, followed by all author rebuttals in order by version.

Version 1:

Reviewer comments:

Referee #1

(Remarks to the Author)
This paper describes the FLIP PSF subtraction algorithm, which uses a physics-based auto-differentiated model to optimally estimate and remove starlight. The results are compelling and significant, with on-sky demonstrations achieving substantially higher SNR than the current state-of-the-art. The topic is also timely, both given the application to JWST as well as the desire to achieve the photon noise limit at all separations for HWO. The methodology is sound. Most of the conclusions are robust, though several could be improved or better justified.

The paper is well-written and clear. Figures support the paper, and it generally provides adequate detail given the format desired for this journal. Additional references are needed in a few places.

Below are issues identified with the paper. Many of these (labeled “minor”) are simple points of clarification. A handful are seen as more pressing (labeled “significant”) and need to be addressed.

I recommend this paper for publication once the issues below are addressed.

Significant issues:

1. Issues with stability claims (lines 59, 98, 176): the authors allude to the potential for methods like this to relax stability requirements of HWO, claim that their results show the potential to surpass stability limits, and claim their results beat “the stability limits of JWST.” What precisely is meant by this? There is no reference for this claim, it does not seem to be discussed in detail, and I see no explicit demonstration of it. The authors must justify this and show it explicitly in their results to make such a claim. If the authors are assuming that the comparison between KLIP and FLIP with JWST data shows this, they must also show that other factors were not limiting the KLIP performance (e.g., the choice of reference star, target acq errors, etc. -- I would consider target acq errors a repeatability issue, not a stability issue, but am open to hearing otherwise).

2. Line 164: the authors incorrectly claim that “only model-based PSF subtraction is capable of reaching…” the noise limit. This makes many assumptions about the magnitude of the instabilities. The curves in Fig 3 show that this doesn’t happen at the majority of separations in their work. Further, there are alternative instrument designs and ConOps that, in theory, could help to greatly mitigate the speckles. This sentence should be rephrased.

3. Issues with SNR measurement:
- The authors show much higher SNR than other reduction methods. While I agree that this almost assuredly means better performance (which is obvious from some of the figures), SNR does not fully communicate the performance of the data reduction process. Ideally the authors would report a confidence level. As shown in Mawet et al. (2014), the confidence can still be quite low given the limited number of samples at small separations. The authors must also clearly describe how they measured that confidence level (e.g., using measured standard deviations and t-test distributions vs Monte Carlo methods)
- When the authors measure the SNR, they take the standard deviation of all pixels in a thin annulus as the noise and use the peak of a Gaussian fit to the planet. Presumably the peak of this Gaussian adopts the same pixel resolution? Their

results would not appreciably change if they were to use the brightest pixel, correct?
- Do you assume you know where the planet is when measuring SNR, or are you simply selecting the location of brightest flux/best fit to a Gaussian?
- Figures show images in terms of counts. It would also be helpful to see SNR maps. This would highlight any false positives in the images (if they exist).

4. A number of parameters of the reduction are “tuned” (e.g., see line 292). Line 378 states explicitly that different values were used for each data set without much of an explanation why. When tuning these parameters, presumably you are watching the planet and seeing what makes the planet “pop?” Without knowledge of what these parameters need to be ahead of time, this parameter tuning is technically part of the detection process and ideally should be captured in the confidence level. The authors should discuss this statistical issue.
- The authors inject planets and monitor their SNR during the model fitting process to avoid over-subtraction of the planets. Are the fake planets injected at all separations and angles? How can one be sure that maintaining the flux of an injected planet at an alternate location in the image maintains the flux of the real planet? Was this tested?
- When tracking the signal reduction of injected planets (in order to stop the optimization process), is the optimization done at all separations simultaneously, or is each separation separately optimized in thin annuli?

Minor issues:

1. Line 86: The authors reference “instantaneous pointing.” Does this account for high frequency jitter? Does JWST provide you with data to be able to include this?

2. Line 94: This sentence is probably not technically accurate—I think the authors mean that this is the first time a physical model has revealed a planet when empirical reference observations haven’t? Even with that change, I suggest that the authors rephrase this sentence to reference prior work and provide better context. Other efforts have considered model-based PSF subtraction and it would be good to acknowledge those (e.g., Lawson et al. 2024 is a recent one).

3. Fig 3: Why does KLIP outperform FLIP beyond 2 arcsec for TYC 5899? Why does FLIP outperform the noise floor at 1.8 arcsec for 51 Eri?

4. Line 168: How much do these various terms limit your results?

5. Line 255: There should be a reference for the charge diffusion Gaussian kernel treatment

6. Line 275: The two-stage optimization is nice. Probably useful to remind the reader that the reference star is known not to host bright planets

7. Line 288: Why is the optimization more stable if you only solve for the OPD after the mask instead? Is this a numerical limitation or does this physically mean something for JWST?

8. Line 290: Reference for the Adam optimizer?

9. Line 331: This is an interesting result. Presumably the results are not as good on the time-resolved data because you treat each individual frame as uncorrelated? If one were to treat it as a correlated data cube, presumably one could only do better (e.g., knowing that the planet has only a DC component while the speckles can vary temporally). Can these sorts of correlations be included in the fitting process?

(Remarks on code availability)

Referee #2

(Remarks to the Author)
- Summary:

This manuscript reports new improvements in starlight suppression for coronagraphic direct imaging of exoplanets, in particular, directly improving sensitivity and detectability in archival data. The article presents a new PSF modeling approach that combines physics-based modeling of the telescope and optics with GPU-accelerated computation, machine learning, and automatic differentiation, applied to coronagraphic instruments. The authors demonstrate this new methodology on JWST/NIRCam archival data, achieving better sensitivities compared to a state-of-the-art post-processing technique, KLIP. They also estimate the fundamental contrast limit of each JWST/NIRCam observations, and show that their method reaches sensitivities close to this limit, performing better than the other post-processing technique. In addition, they recover two known exoplanets with higher S/N and detect one known exoplanet that was not recovered in previous studies. Overall, this method shows strong potential to improve exoplanet detectability, as demonstrated by the improvement in sensitivity and speckle suppression in JWST/NIRCam data.
Two main points should be noted. First, the core idea of the method has been presented in a recent published article. While the implementation here is more advanced and applied to coronagraphic instruments, the level of novelty could be better clarified in relation to previous studies. Second, in one of the four datasets, the implementation of the “classical” post-

processing appears suboptimal, which may bias and affect the comparison with the proposed method in that specific case. However, these points do not significantly impact the main results and conclusions of the manuscript, which remain robust and of great interest for the community.

- General comments:

General comment 1: It is unclear how the author processed the data: observations such as HIP65426 were carried out using two roll angles. How did the authors process the data using KLIP? For example, it is possible to combine RDI+ADI, or just RDI and de-rotated the data and combine them. Is the stack performed using mean or median?

General comment 2: I am wondering if the flux is being biased using this new method. It would be great if the authors could provide the flux measurement and uncertainties of one planet (e.g., HIP65426b), and then compare this value to the published one. The authors are free to consider this comment, since it goes beyond the core of this study; however, the corroboration of flux calibration is also important, but could be investigated in future publications (e.g., the detection of 51 Eri b).

General comment 3: The authors occasionally generalized the results and improvements in sensitivity to JWST as a whole (e.g., line 22/23, line 191, line 206), while the method is demonstrated using JWST/NIRCam data only. It would therefore be more appropriate to refer specifically to JWST/NIRCam. Other JWST observing modes and instruments (e.g., MIRI+4QPM) have also shown strong/great coronagraphic performance but involve different coronagraphs/optics, observational strategies, and post-processing challenges and improvements (e.g., Boccaletti et al. 2022, https://ui.adsabs.harvard.edu/abs/2022A%26A...667A.165B/abstract ; Carter et al. 2023, https://ui.adsabs.harvard.edu/abs/2023ApJ...951L..20C/abstract ; Boccaletti et al. 2024, https://ui.adsabs.harvard.edu/abs/2024A%26A...686A..33B/abstract ; Godoy et al. 2024, https://ui.adsabs.harvard.edu/abs/2024A%26A...689A.185G/abstract ; Malin et al. 2024, https://ui.adsabs.harvard.edu/abs/2024A%26A...690A.316M/abstract ; Lagrange et al. 2025, https://ui.adsabs.harvard.edu/abs/2025Natur.642..905L/abstract). Clarify this point and distinction would better reflect the findings, impact, and scope of this study. I also suggest to mention the incorporation of JWST/MIRI in the method and public codes as a future work, if this is part of the short-term plan of the authors.

- Major comments:

Line 19/20/21: "The key innovation that makes this feasible is a differentiable optical model enabled by modern machine learning frameworks with automatic differentiation …" I am wondering about this statement, since the "innovation" is not longer applicable. A recent article accepted in Astronomy and Astrophysics (Kuznetsov et al. 2026, accepted 19 February 2026, https://ui.adsabs.harvard.edu/abs/2026arXiv260323339K/abstract) reported the successful modeling of PSF using physics-based, GPU-accelerated, with differentiable optical model coupled with neural network and automatic differentiation. Since the mentioned study is focus on ground-based telescopes, and only in "classical" PSF, I would re-phrase the sentence to focus more in space-based telescopes and, directly, coronographic stellar PSF, which is the main core of this article. I present more comments about this point in the "line-to-line comments". I want to emphasise that the result reported here has an impact in exoplanet direct imaging, and it is the first time that this method is implemented in coronographic-PSF instrumental modeling, therefore this must be highlighted accordingly throughout the entire manuscript.

Line 373-375: I am wondering about the approach the authors used here. The simplification for "apples-to-apples" comparison is well far from what the community is doing. I would like first give some context: reference stars are observed as close as possible in time to avoid as much as possible the wavefront and thermal drift variation and, in most of the cases for space-based telescopes, it is adopted the so-called dither-patter. This dithering is created to introduce more diversity in the PSF library and capture spacial variation due to an imperfect centering/pointing. As far as I know, the self-reference method adopted in different surveys used the fact that they have a large library composed by stars observed in different times with different pointing/centering. This generates a PSF library rich in diversity, which mimic the classical observation of a reference star. Some studies such as Xie et al. 2022 (https://ui.adsabs.harvard.edu/abs/2022A%26A...666A..32X/abstract) shows the efficiency of using these libraries to reach a better performance in sensitivity and speckle removal.
The approach adopted by the authors, by definition, will reach the poorest/worst contrast/sensitivity using KLIP or any other method. This is because the diversity in both dithering and speckle variation is not captured by a single observation (which is separated by a significant amount of time and, therefore, considerable difference obs conditions, and different pointing error). This generates a considerable mismatch between the reference star and the science target. Even for space-based telescopes with an unprecedented stability such as JWST, the differences due to thermal and wavefront drift and pointing errors can generate significant mismatches.
The authors can check this by using the same approach with the HIP65426, by using a different star wide separated in time, and using only one of the dither patterns. The authors will note a degradation in sensitivity and ability to suppress the speckles. This point need to be taking with caution.
In conclusion, the approach adopted here produces the worst performance in contrast for the KLIP. Therefore, the authors cannot compare a contrast don't use by the community, which generates the poorest performance, with their method. It is also important to highlight that in Bogat et al. 2025 (paper cited by the authors for TYC5899), the contrast reached by the survey using the self-reference library, is about ~3e-6 at ~2.5 arcsec, an order of magnitude deeper than the one reported here (~1e-5 at ~2.5 arcsec). Also ~3e-4 at ~0.6'' vs 1e-3 at ~0.5'' reported here.
I want to highlight that the idea of using a single star as reference in surveys is a good proof for the method described here

(FLIP), which demonstrates that it is not necessary to have a close-in-time reference star observation (and non dithering patter) to reach a good performance. This can be of great interest for the community for planning future observations, and a motivation to explore the archive data.

- Minor/medium highlighted comments:

Line 406-407: “We generate a random Gaussian sample with mean 0 and σ equal to the total noise level in each pixel” It is not entirely clear how the authors propagate the uncertainties here. The ERR contains all the noise contributions, and exists one per integration. In the case of KLIP, it is not trivial to know how this is propagated through the pipeline. In the most simplistic scenario, the ERR of the final stacked image is the quadratic sum of all the ERR from each integration. The contrast curve then can be calculated using the FPF with the mean value being the noise level. It is unclear why the authors used Gaussian centred at 0 and using sigma = ERR to generate a “random Gaussian sample”. This generates new images with negative values, which is conceptually wrong when talking about noise/uncertainties. Following the article cited by the authors (Godoy et al. 2024), the noise propagation looks similar to the case 0 presented in that study. As is mentioned in that study, there are some assumptions that have been done and need to be mentioned explicitly here. For instance, the non-correlated noise between nearby pixels. If the noise propagation was done following the ideas presented in that article, please cite it accordingly.
Since the fundamental sensitivity limit is one of the core ideas in this article, I encourage the authors to explain the procedure more clearly and in detail, or cite directly the mentioned article and provide a slightly more extended summary about the procedure.

Line 425/426: “While this may not be optimal for exoplanet recovery given the differences in …”. It is not clear if the authors did this just to show the figures, or to quantify the S/N as well. In the case of S/N, it is not clear if the authors optimize the extraction of the S/N for FLIP and KLIP, or just keep one single configuration for the extraction in the entire FoV. It is not a guarantee that using the same KL modes the authors can obtain the best S/N for KLIP. This is a bias when comparing both methods, and need to be clarified. Also, are the chosen number of iterations the optimal ones, or the most pessimistic ones?

Line 434: “… and it still causes some planet signal loss.”. I am wondering about this statement. Are the authors suggesting that the throughput correction will not be needed at some point using this method?. From what the authors mentioned throughout the article, this technique is limited by several factors that are not even possible to control or model. And as any other technique, the model of the PSF will never be absolutely perfect and, therefore, it requires a throughput correction to account for flux loss. The throughput can be minimized as much as possible, but still needed to account for any type of biases. I strongly suggest the authors to modify this sentence. Also, please provide the throughput correction for the FLIP and KLIP for the 4 datasets presented in this study. This will better support the statement here, and will provide how better is the modeling of the PSF.

- Minor comments - line-to-line comments:

Line 5: correct the citation: https://ui.adsabs.harvard.edu/abs/2023ASPC..534..799C/abstract

Line 8/9: “While advances in coronagraphy and telescope stability are increasing the sensitivity of our observations, …” I suggest to point directly to space-based telescopes, since this is the focus of the article.

Line 22/23: “We demonstrate our method on observations from the James Webb
Space Telescope (JWST)”. Please, be specific here since this study does not explore all the coronographic modes of the JWST (e.g. MIRI).
Suggestion: “We demonstrate our method on observations from the James Webb
Space Telescope (JWST), Near Infrared Camera (NIRCam) coronographic mode.”
Also, provide the proper citation: JWST - Gardner et al. 2006
https://ui.adsabs.harvard.edu/abs/2006SSRv..123..485G/abstract ; NIRCam Rieke et al. 2005
https://ui.adsabs.harvard.edu/abs/2005AAS...20711509R/abstract, Krist et al. 2009
https://ui.adsabs.harvard.edu/abs/2009SPIE.7440E..0WK/abstract

Line 23/24: “We recover previously undetected planets and improve detections of known planets with sensitivity ...” Here, I recommend to highlight that these are known exoplanets, otherwise could be interpreted as new discoveries rather than improvements in the data processing. Also, in this study it was recovered just one know (exo)planet that was previously undetected, so I recommend to place the statement in singular.
Suggestion: “We recover previously undetected known planet and improve the detections of those known recovered planets with sensitivity …”

Line 23: I strongly suggest mentioning the detection of 51 Eri b since this provides strong support to the technique, the real impact on detectability and how much the sensitivity was improved.

Line 39: please provide a reference here (e.g., Currie et al. 2023,
https://ui.adsabs.harvard.edu/abs/2023ASPC..534..799C/abstract)

Line 43: Since for ground-based telescopes there are more factors than the ones mentioned here, I suggest to refer directly

to space-based telescopes.
Suggestion: “... and instrumental aberrations when observing with space telescopes.”

Line 49: “… are fundamentally limited by instrument instabilities, such as telescope pointing errors and thermal drifts.” I suggest to be more explicit here, focusing on the space telescopes. Otherwise it should be mention, for example, turbulence.
Suggestion: “… and thermal drifts on space telescopes.”

Line 91: Following my suggestion in Line 22/23, here should be: “from the JWST/NIRCam”

Line 91/92/93: “FLIP produces better planet detections than standard methods in published datasets, and in some cases it reveals images of planets that had remained undetected by traditional techniques.” The sentence is too strong to respect the results presented in this work. Claim for “better planet detections” and “reveals images of planets that had remained undetected” is more linked to a successful detection of new planets (that are possible only with this technique), than to an improvement on sensitivity. What the authors should highlight here is the improvement in sensitivity/detectability and recovery of know planets.
This sentence must be re-phrased.
My suggestion: “FLIP improves sensitivity and signal-to-noise ratio relative to standard methods, enhancing planet detectability and enabling the recovery of a known planet not detected by traditional techniques.”

Line 94/95/96: “After over thirty five years of repeated community efforts, this work is the first time that exoplanets are revealed in coronagraphic imaging data using a physical model instead of empirical reference observations.”. The part of “of repeated” sounds too strong here. I would suggest a more formal phrase, highlighting both the results and the community effort.
Suggestion: “After over thirty-five years of community-wide efforts, this work is the first time that exoplanets are detected in coronagraphic imaging data using a physical model instead of empirical reference observations.”

Line 102/103/104/105: Since the recent published article (Kuznetsova et al. 2026, accepted 19 February 2026, https://ui.adsabs.harvard.edu/abs/2026arXiv260323339K/abstract), use the same principle based on physics-based modeling and automatic differentiation, I strongly suggest to the authors to re-phrase this paragraph, since the “innovation” here is not longer applicable in the way it is presented. Please re-phrase it being sure the proper references appears clearly here.
I have two suggestions:
1) “… consists of two primary methodological innovations, building on previous work (cite the paper here) and adapted here to space-based coronagraphic instruments: ...”
2) “… consists of two main methodological advances (cite the paper here), here applied to space-based coronagraphic instruments: …”
N.B.: the authors can claim for the novelty in the application to coronographic PSF reconstruction, but giving credits to previous and recent works done in the PSF modeling in general.

Line 107: “… real JWST data.” Since here the authors present the model for one observing mode, I strongly suggest to be more precise.
Suggestion: “… real JWST/NIRCam coronagraphy data.”

Line 119: Authors could add “… close in time and at a small angular separation …”

Line 122: “… by injecting synthetic planets close to the sensitivity limit and tracking the signal-to-noise ratio (SNR) of the fake planet detection.”. I have several questions about this sentence: 1) It is not entirely clear how the authors obtain the contrast curve (sensitivity) to be able to inject the point-sources. Is it obtained from state-of-the-art post-processing techniques? Or is it coming from a really rough first iteration of the method? 2) What do the authors mean by “close to the sensitivity limit”? Is it a 3-sigma, 5-sigma, or 10-sigma? Please be more precise when providing this type of information. 3) how many point-sources are injected per annulus, and how this number impact the performance in terms of sensitivity and computational time? I also recommend that the authors to change “synthetic planet” by “point source”, or use “injected fake planet” instead.

Lines 130/131/132: When referring to the observations of the 3 stars HIP65426, 51Eir, and TYC5899, please mention the filters explicitly, since this is not something obvious. For example, Carter et al. observed HIP65426 with different filters, modes, and instruments.

Line 138: Can the authors provide numbers more than just “robust detection”? Is the detection at 5- or 20-sigma level? What is the FPF or the metric used to define it as “robust detection”?

Line 141: “…, we inject fake planets on both data sets to illustrate the …”. Can the authors explain what are these two data sets they are referring to? Is it two roll angles or two different epochs? Or two different filters?

Line 146: I suggest that the authors also mention the S/N of the detected planet. This is a major proof of the method presented here, and demonstrates the direct impact on the direct imaging campaigns. The authors should highlight this more throughout the article.

Line 175/176: “our technique FLIP achieves exoplanet detection sensitivity beyond the stability limit of JWST”. This can be

interpreted as FLIP can go deeper than instrumental limits, or that the stability limit is not actually a limit. What the authors probably want to say here is that this technique can mitigate better the limitations set by the PSF variability and wavefront evolution. Please re-phrase this sentence.
Suggestion: "Our technique achieves detection sensitivities beyond those reached by standard methods, effectively mitigating limitations imposed by JWST stability."

Line 180: As I mentioned in previous comments, I encourage the authors to be more precise with the information provided in the text. As the method is demonstrated using JWST/NIRCam observations, it would be more precise to refer specifically to JWST/NIRCam rather than JWST in general.
Suggestion: "… planet search surveys with JWST/NIRCam. …"

Line 190: "FLIP could therefore be instrumental to unlocking the true sensitivity of JWST to characterize the atmospheres of the Gaia planets.". The term "true sensitivity" is too strong, as it implies a unique, physically defined limit (limit equal to the sensitivity reached by FLP), whereas the method improves the achievable sensitivity. This phrase must be re-written.
Suggestion: "… unlocking the best performance in sensitivity of the JWST/NIRCam to …"

Line 198: I suggest mentioning the recent article published in A&A (see my comments above), and highlighting the main different aspect from that study. For instance, the authors suggest the use of optics telemetry, while – as far as understood – the other article did not.

Line 199: "… telemetry is stored and made publicly available.". I am wondering if the authors mean here that the method is not applicable to archival data but to future observations. If this is not the case, I suggest to rephrase to highlight that other options are possible as well.

Lines 194-202: I suggest to mention the instruments, modes and wavelength that would be benefit the most from applying FLIP. For example, are the authors expect that FLIP generates major impacts at shorter wavelengths (H band) or longer wavelengths (L-band, N-band)? Also, it could be worth mentioning other sources that can greatly affect or challenge the implementation of FLIP in ground-based telescopes. For instance, the wind speed effect (e.g., in the VLT Cantalloube et al. 2020, https://ui.adsabs.harvard.edu/abs/2020A%26A...638A..98C/abstract), or petal-effect (e.g., Wang et al. 2024, https://ui.adsabs.harvard.edu/abs/2024SPIE13097E..2FW/abstract ; Levraud et al. 2024, https://ui.adsabs.harvard.edu/abs/2024SPIE13097E..2EL/abstract).

Line 206: " … shown for JWST, however, …". Following my previous comments, this should be re-written.
Suggestion: "… shown for JWST/NIRCam, however, …"

Line 206-208: "In this work we have shown for JWST, however, that model-based PSF subtraction can model these very instabilities and achieve contrasts better than the limit set by the thermal and mechanical drifts of the telescope.". This phrase could suggest that the FLIP method can reach sensitivities better than the physical telescope limits. The authors probably want to highlight that the FLIP method can mitigate the thermal variation and wavefront drift, which greatly affect current state-of-the-art post-processing techniques. I strongly recommend to re-write this sentence.
Suggestion: "In this work we have shown for JWST/NIRCam coronagraph, however, that model-based PSF subtraction can account for these instabilities and achieve contrasts beyond those reached by current methods, which are effectively limited by thermal and mechanical drifts of the telescope."

Line 345/246: "…, the OPD measurement closest to the time of observation …". I am more curious to know if the authors test with different OPDs near and far in time. It is known that the OPD changes on time, and that the closest one is not necessarily the one that best represents the observation. This is because it could happens specific events that generates larger differences between two consecutive OPDs (see for example, the WFE evolution: https://jwst-docs.stsci.edu/jwst-observatory-characteristics-and-performance/optics-performance-and-stability#gsc.tab=0). As I understood from this work, the OPD represent a starting point in the modeling, so an OPD that is less representative of the observations should 1) produce a wrong model and therefore poor performance of FLIP, or 2) the method just need more time to converge to the best solution and performance. Can the authors clarify this? This is my pure curiosity; however, it can have a really high impact on the PSF-modeling for archive, current, and future programs with JWST/NIRCam.

Line 261: "… , but this formulation showed the best results.". Can the authors be more precise? What is considered "best result"? What metric was used to conclude that? And how much better in the frame of that metric? (e.g., 2 times better in the rms, or 5-sgima deeper in sensitivity performance).

Line 316: The observations of the reference star are usually done using a dither-patter to increase the diversity on the PSF-library by capturing different PSFs. Are the authors using all the dither observations to generate the best calibration? In the case the authors are keeping just one, how was this one selected? And what is the impact of choosing one of the other dither observations? For instance, Carter et al. used a 9-dither-patter, which means 9 reference star observations. Are the OPD solutions and stellar flux the same between all the reference star observations? I suggest the authors to show these stellar fluxes as part of the validation of the technique.

Line 325: How may injected fake planets are used in the optimization? And are these injected in the entire FoV, or just in regions dominated by the stellar chronographic PSF? How much does affects the choice of the flux in the optimization? For instance, a S/N of 5, or 20, or 100 can produce the same final performance, or generate really different results? And in terms of computing time, is it faster to use higher S/N?

Line 332/333: The authors mean they used the mean-combined frames? If yes, what is the tolerance to do this in terms of performance? For instance, combine frames over 5min or 1h can produce significant differences?

Line 336/337: Related to my previous comment: did the authors check the integration time of the observations? The conclusion that time-average the observations could be correlated with the integration time rather than the average itself.

Line 340: Please mention which filter explicitly.

Line 341: "We run standard PSF subtraction method KLIP". Related to one of my previous comments: is the pipeline run using RDI or RDI+ADI? Are the authors using both roll angles? I encourage the authors to be more precise with this type of information.

Line 352: Please provide the pipeline version used here, as well as the version used of the different tools associated to the data reduction and post-processing, in order to be able to reproduce the results.

Line 382: Regarding the S/N (and related to previous comments): how is the S/N of the injected point source impacting this value (11000 iterations)? For example, a source injected with a S/N of 3 will require the same iteration as one with S/N of 30? Also, and as I mentioned before, it is not entirely clear how the authors compute the first contrast/sensitivity to be able to inject sources that can be detected at a certain S/N.

Line 392/393: Since the pipelines (JWST, spaceKLIP) have considerable improvements from, for example, Carter et al., it is important that authors explain why they used the same parameters and not an optimization to obtain the best sensitivity performance. Also, I encourage the authors to explicitly mention the pipelines and tools versions used in this study, and the ones used in the respective publications. Regarding one of my comments about TYC5899, the sentence must be re-phrased since the contrast obtained is largely in disagreement with the one reported in the literature.

Line 405: "… which track the combined total uncertainty in each pixel value from read noise and Poisson noise.". The authors forgot to include the Flat noise here. Please add it.

Line 418: "… we inject synthetic planets …". I suggest the authors to use the nomenclature from the articles and the community. In this case, "inject fake planets", or "inject point sources" should be more appropriate. The "synthetic planets" sounds more to an injection of planets following a evolutionary track or atmospheric models from which the flux at that bandpass was extracted (which is not the case of the procedure followed in this study).

Line 421: "… we inject a planet with flux equal to 80 times the …". Can the authors justify why the choose of 80 and not 100 or 20? Is this value related to a better performance of the method, or to reach a reasonable S/N in both KLIP and FLIP? Or is it just linked to the best compromise between performance and computational time (iterations)?

Line 432/433: "While our experiments show significant improvements relative to standard techniques on a variety of …". I strongly recommend the authors to keep the findings and conclusions within the results presented here. There are several "standard techniques" (that should be mentioned as state-of-the-art techniques) – for example ANDROMEDA (Cantalloube et al. 2015), PCA (Soummer et al. 2012), NMF, (Ren et al. 2018), LLSG (Gomez Gonzalez et al. 2016), PACO (Flasseur et al. 2018,2021), TRAP (Samland t al. 2021) – that are not studied non investigated here.
Suggestion: "… relative to the state-of-the-art technique KLIP ..."

Line 453: I suggest the authors to refer directly to JWST/NIRCam instead of just JWST.

Line 470-448: I encourage the authors to cite the already mentioned recent paper (Kuznetsov et al 2026, https://ui.adsabs.harvard.edu/abs/2026arXiv260323339K/abstract) and to compare the ideas, challenges, and solutions between this paper and the authors study here.

Line 431-488: If the authors are planning to incorporate other observing modes, strategies and/or instruments such as JWST/MIRI, I suggest to include them in this section.

- Figures and tables:

Fig. 2: Please change the label of the color-bar "Counts" by "MJy/sr" or "Counts [MJy/sr]"

Fig. 3: For TYC5899 the contrast is notably worse than the one reported in the respective article. I strongly suggest using the published contrast curve instead of the one computed here.

Fig. 5: Please provide in the color-axis the respective units. Improve the figure quality as well.

Fig. 6: Can the authors explain each panel for a more comprehensive view? Also, please all the finding and interpretations about the figure, must be move to the main text. In addition, please incorporate the color-bar units (e.g., W/m2/microns, Jy/sr, electron counts, arbitrary units).

Fig. 7: “The nearby off-axis bright star is also visible.”. Can the authors indicate explicitly where this bright star is (even if this is obvious)?

Fig. 8: Is the color-scale and limits the same as for Fig. 7? Since the idea is to show if the injected point-sources are visible or not, I suggest that this figure has its own color-scale and limits.

Table 1: caption “… PSF with very high accuracy …”. This following one of my previous comments: can the authors provide quantitatively what they mean with “high accuracy”?

(Remarks on code availability)

Referee #3

(Remarks to the Author)
A. Summary of key results

The manuscript presents a carefully executed and technically solid study applying a data analysis pipeline called FLIP to the detection and characterization of planetary signals using an automatic-differentiation-based optimization framework. The work is clearly written, and the methodology is well structured and clearly described, The approach shows a meaningful performance improvement over existing techniques as it can be seen on the detected planet SNR improvement and will likely be of interest to specialists in the field.

The authors have made a solid effort to achieve improved contrast levels through their post-processing pipeline, and the manuscript is clearly written and technically competent. However, I believe there are significant shortcomings in the current form of the work. I outline my most important concerns below.

In its current form, the central contribution is not sufficiently clear or compelling at that level, both in terms of methodological novelty and demonstrated scientific impact. A recurring concern throughout the manuscript is that several key claims appear stronger than what is directly supported by the analysis presented, making it difficult to assess the true level of advance.

B. Originality and significance

From a methodological perspective, the approach is best understood as a combination of established components rather than a fundamentally new framework. It relies on physics-based forward modeling of instrumental effects and gradient-based optimization techniques, including automatic differentiation. Both elements are well established in the literature for inverse problems in computational imaging and astronomical data analysis. In addition, differentiable forward modeling frameworks of this type have been introduced in prior work and form the basis of the present implementation (as referenced in the manuscript).

Thus, while the approach appears to improve performance relative to the current state of the art, it is not yet convincingly demonstrated how this improvement relates to prior implementations of the same underlying methodological framework. In particular, it remains unclear whether the reported gains reflect a genuine step-change in capability or an incremental improvement arising from implementation choices, tuning, or application-specific adaptations. A more explicit, quantitative, and controlled comparison to prior implementations of this approach would be necessary to establish what is genuinely new in this work and to justify the implied level of methodological advance.
In this context, the manuscript appears to extend and apply an existing differentiable modeling and optimization framework rather than introduce a new methodological paradigm. Automatic differentiation, in particular, is a standard computational tool (implemented in widely used machine learning libraries such as PyTorch) and does not constitute a novel contribution. The main novelty therefore appears to lie in the application and integration of these established components to a specific dataset, rather than in the underlying methodology itself.

C. Data & methodology

Beyond questions of novelty, a more fundamental limitation of the manuscript is the limited interpretability of the reported performance improvements. While the method demonstrates improved detection capability, the manuscript provides limited insight into the mechanisms responsible for these gains. It remains unclear which components of the pipeline are primarily driving the improvement, or under what conditions the method may succeed or fail. As a result, the approach appears effectively “black-box-like” in its current form, where performance gains are observed but not sufficiently explained in terms of underlying model behavior or physical intuition. This lack of mechanistic understanding is closely linked to the broader concern that the inverse problem is not rigorously validated.

D. Appropriate use of statistics and treatment of uncertainties

A further concern relates to the treatment of the underlying inverse problem. The manuscript indicates that the method estimates instrumental parameters, as well as source position and flux; however, no quantitative validation of these inferred parameters is presented. Given the strong degeneracies inherent in this class of problems, it is not demonstrated that the

method is actually recovering physically meaningful parameter estimates, rather than primarily producing image-domain reconstructions that match the adopted metrics. In particular, good detection or reconstruction performance alone is not sufficient to establish that the inverse problem has been properly solved. This concern is further reinforced by the manuscript's own acknowledgment that the problem is ill-posed and degenerate, with multiple solutions potentially producing similar observational outcomes, which is deferred to future work rather than addressed in the present analysis. In this context, the interpretation of inferred parameters appears insufficiently supported.

E. Conclusions: robustness, validity, reliability

E.1 Scientific overstatement (results interpretation)
The manuscript emphasizes potential scientific insights that could be derived from future analysis of these detections, rather than demonstrating such advances within the present work. As a result, the scientific impact remains largely prospective rather than realized. In particular, the claim of recovering "previously undetected planets" is not supported by the analysis presented, which is limited to re-detection of previously known objects in a different observational band.

E.2 Unsupported methodological claims
A similar issue arises in several methodological statements that are insufficiently supported by quantitative evidence. For example, the claim that the technique achieves exoplanet detection sensitivity "beyond the stability limit" is not convincingly demonstrated, as the stability limit is not clearly defined or quantitatively established, and no direct comparison is provided. Likewise, the statement that "four groups of free parameters are generally sufficient for PSF fidelity in most cases" is not supported by a systematic evaluation or sensitivity analysis and requires either quantitative justification or more careful qualification.

E.3 Robustness and completeness of experimental validation
The robustness of the reported results remains unclear. The manuscript presents a limited number of experiments without a systematic exploration of parameter space or observational conditions. As a result, it is difficult to assess whether the reported performance is representative and general, or whether it may depend on favorable or selectively presented cases. A more comprehensive and statistically grounded evaluation would be important to establish the reliability and generality of the method.

F. Suggested improvements / Specific major comments

-The authors should more clearly quantify the performance gains of the method relative to prior work, and clarify whether the advance represents a substantive step-change or an incremental improvement.

-"We recover previously undetected planets"
=> This claim should be revised for accuracy and consistency with the results presented.

-"By explicitly modeling the physics of the instrument and leveraging optimization tools from the machine learning community, our technique FLIP achieves exoplanet detection sensitivity beyond the stability limit of JWST."
=> The statement regarding performance "beyond the stability limit" should be either quantitatively substantiated with a clear definition and direct comparison, or appropriately tempered.

-"Given these models, we find that four groups of free parameters are generally sufficient for PSF fidelity in most cases"
=> The claim that four groups of free parameters are "generally sufficient" for PSF fidelity should be supported with quantitative evidence or more carefully qualified.

-"Inferred instrument parameters: Coronagraphy is an ill-posed and degenerate problem, and as such there is a wide range of solutions that may produce effectively the same PSF estimate. Furthermore, the nominal STPSF models for JWST NIRCam, which we use to initialize our differentiable model, are known to be imperfect. To use FLIP to retrieve realistic instrument parameters future calibration work is required."
=> I couldn't agree more and this is why I think this shouldn't be bypassed but rather be part of this study. The manuscript should include a rigorous validation of inferred parameters including uncertainty estimates and comparison to simulations or ground truth, to demonstrate that the inverse problem is meaningfully solved. In addition, a more systematic and statistically robust exploration of performance across observational conditions and parameter space would be valuable to establish the generality and reliability of the method.

- "future work will use this detection to measure the 4.3 µm photometry of the planet and carry out a new atmospheric characterization" "These results are illustrative of the potential of FLIP to aid exoplanet discovery with JWST"
=> The manuscript would benefit from a clearer distinction between demonstrated results and future potential, with appropriate adjustment of the overall framing.

- Reproducibility is currently limited by execution and documentation issues, including reliance on a CUDA-enabled GPU environment without a CPU fallback or sufficiently clear instructions. Providing a minimal runnable example and clearer computational guidance would improve accessibility and independent verification.

G. References

The manuscript appears to appropriately cite relevant prior work. However, the novelty relative to prior work is not always clearly articulated, particularly regarding differentiable modeling frameworks that appear to be the baseline for the present approach.

H. Clarity and context

The manuscript is clearly written and generally well structured. However, several claims in the abstract and conclusions overstate the demonstrated results. More broadly, there is a mismatch between the framing of the work and the results actually demonstrated, with emphasis placed on potential future scientific applications rather than on fully established findings.

(Remarks on code availability)
Supplemental materials review:

“”1. Instructions to run on data
- Run the following bash command inside a virtual environment with all the dependencies outlined above and with access to an NVIDIA A30 GPU (or equivalent):

```
./scripts/run_JWST_test_HIP65426.sh
```

=> While code is provided, the current instructions do not allow straightforward reproduction of the results. In particular, the main execution script does not run as described (there’s no “scripts” folder in the provided package). In addition, reproducing the results is not straightforward. The current implementation assumes a CUDA-enabled GPU environment and does not offer clear guidance on configuration for a CPU fallback. This limits the ability to independently validate the method and assess its robustness, particularly given the broader concerns regarding parameter inference and validation.

Version 2:

Reviewer comments:

Referee #1

(Remarks to the Author)

(Remarks on code availability)

Referee #2

(Remarks to the Author)
First, I would like to thank the authors for their excellent work in addressing all of my (our) comments. The manuscript has been substantially improved, both in terms of clarity and presentation. Overall, I am very happy with the revised version and believe that the authors have satisfactorily addressed my main comments. I also appreciate the authors' detailed responses to my questions and the additional clarifications provided both in their responses and in the revised manuscript.

I have a few minor corrections and comments that I believe require the authors' attention (mostly minor editorial and English-writing suggestions):

1) Lines 110–112:
“After over thirty-five years of community-wide efforts, this work is the first time that model-based PSF subtraction surpasses standard techniques in coronagraphic exoplanet imaging, with up to an order of magnitude improvement in sensitivity.”
I am wondering about the rephrased version. From my point of view, the sentence could be read as meaning that there have been more than 30 years of efforts and that this is the first occasion on which model-based PSF subtraction has surpassed standard techniques. However, I do not think this is exactly the intended meaning.
As I understand it, the intention is to highlight that, after more than 30 years of community-wide development of post-processing techniques for coronagraphic direct imaging of exoplanets, this work demonstrates for the first time that a model-based PSF subtraction approach can outperform the standard techniques in terms of sensitivity. I want to emphasize that the difference is that the phrase above sounds more like "there were multiple times in which the community tried to implement this, reaching worst sensitivities, and this is the first time the sensitivity is better than the ones from traditional techniques".
I have two suggestions, that keep the 30-year effort, the novelty, and the improvements, although the authors are of course free to rephrase the sentence as they prefer, as long as the core idea is conveyed more clearly:

Option 1: “After more than 30 years of development in coronagraphic exoplanet imaging, we demonstrate for the first time that a previously untested model-based PSF subtraction approach can outperform standard techniques, improving sensitivity by up to an order of magnitude.”
Option 2: “After more than 30 years of relying on standard techniques, we demonstrate that a previously untested model-

based PSF subtraction approach can outperform them in coronagraphic exoplanet imaging, improving sensitivity by up to an order of magnitude."

2) Line 176
The sentence "... is shown in the figure" would be clearer if the figure number were explicitly provided (Figure 2), for more comprehensive reading.

3) Line 365
Figure 10 appears to be mentioned before Figures 8 and 9.

4) References
Some references appear to need updating, in particular references [26], [30], and [31].

5) Figure colorbars
Figures 5, 6, 7, and 10 show "MJy/sr" or "[MJy/sr]" as the colorbar label. These are units and should be accompanied by the corresponding physical quantity. This is equivalent to putting "[arcsec]" alone in Figure 8 instead of "Separation [arcsec]". The label could be, for example, "Flux density [MJy/sr]" or "Surface brightness [MJy/sr]", or "Counts [MJy/sr]", as used in Figure 2. However, since the images are flux-calibrated, I would recommend the first option.
Figures 11 and 12 do not have colorbars. Although a colorbar is not strictly necessary here, including one would help the reader interpret the values shown in the images.

6) Figure captions
In general, the authors may wish to check the figure captions with the editor, as some of them appear to include information that would be more appropriate in the main text.
In my understanding, the caption should explain what is shown in the figure and provide the additional information needed to interpret the figure, plots, images, etc. The first sentence should briefly state what the figure shows, while the following text should describe the different elements of the figure and provide information that helps the reader understand it. Results, interpretations, and conclusions should generally be presented in the main text, in the paragraph where the figure is discussed, rather than in the caption. There may of course be exceptions, but I would recommend following this general structure and checking with the editor.

I will point out the ones I consider need further attention:

Figure 1: The first sentence is fine. However, the authors do not explicitly explain what panels (a) and (b) represent or what they show. The rest of the caption is understandable, but it should be more directly connected to the different elements of the figure. Alternatively, some of this information could be moved to the main text.

Figure 2: This is, in my opinion, a good example of what the caption should contain.

Figure 3: The caption should be modified. For example, "Our stability estimate matches on-sky KLIP performance within a factor of 2" is more of a result than information needed to interpret the figure. Similarly, "At large separations, the stability limit can be below the noise limited depending on the exposure time of the observations" appears to be an interpretation of the result rather than a description of the figure. This can be inferred by the reader from the figure, and if it is important to highlight this point, I think it would be more appropriate to discuss it in the main text rather than in the caption.

Figure 5: I suggest changing "(stage 1)" to "(stage 1, top images)" and "(stage 2)" to "(stage 2, bottom images)" so that the correspondence with the figure is immediately clear.

Figure 6: The sentence "We see that OPDs are crucial components in our model, both for general model flexibility and to account for differences between reference and science target. Star-coronagraph misalignment, while physically important, is likely absorbed by OPD terms in the form of tip-tilt aberrations, so they do not show important effects in the subtraction quality" is more of an interpretation/result than information needed to understand the figure.
The interpretation should be presented in the main text (by "main text", I mean the paragraph where the figure is cited). If the authors would like to retain some of this information in the caption, it could instead be rephrased to describe what is shown in the figure. For example:
"The images show a noticeable improvement when the OPD is included, as shown in panel X."

Figure 7: Similarly, the statement "We see that both OPDs are necessary for model flexibility, and that even with model flexibility, model calibration is essential to avoid overfitting" is an interpretation of the results rather than a description intended to help the reader interpret the figure. I suggest moving this statement to the main text or rephrasing it so that it describes the content of the figure.

Figure 8: The statements "Therefore our performance is not strongly sensitive to the specific planet(s) we choose to track SNR with" and "We see that running the optimization for too long can cause significant degradation in SNR and increase in signal loss. Tracking injected planets successfully identifies this turning point, and ensures that our stopping condition is located within the SNR maximum or plateau for best sensitivity" are also interpretations of the results. I suggest moving these statements to the main text and keeping the caption focused on describing the figure and the quantities shown.

Figure 10: Similarly, the statement "We find that as long as our learning rates are within the correct order of magnitude, the

PSF subtraction result is not strongly dependent on the specific choice of the learning rate (middle). The appropriate order of magnitude for the learning rates is obvious after a coarse search" is a result/interpretation rather than a description of the figure. I suggest moving this discussion to the main text and keeping the caption focused on explaining what is shown in the different panels.

(Remarks on code availability)

Open Access This Peer Review File is licensed under a Creative Commons Attribution 4.0 International License, which permits use, sharing, adaptation, distribution and reproduction in any medium or format, as long as you give appropriate credit to the original author(s) and the source, provide a link to the Creative Commons license, and indicate if changes were made.
In cases where reviewers are anonymous, credit should be given to 'Anonymous Referee' and the source.
The images or other third party material in this Peer Review File are included in the article's Creative Commons license, unless indicated otherwise in a credit line to the material. If material is not included in the article's Creative Commons license and your intended use is not permitted by statutory regulation or exceeds the permitted use, you will need to obtain permission directly from the copyright holder.
To view a copy of this license, visit https://creativecommons.org/licenses/by/4.0/

We thank the Editor and the Referees for the careful reading of our manuscript, as well as the detailed comments that have greatly improved the clarity and quality of the manuscript. We summarize the major changes to our manuscript below:

- **Method contributions and innovations: We have expanded on and clarified the main contributions of our work in relation to previous work. In particular we state more clearly and explicitly the modifications that make this work the first successful application of autodiff-enabled, model-based PSF subtraction on on-sky coronagraphic imaging, yielding sensitivity gains of up to an order of magnitude relative to standard starlight subtraction approaches. In the Main text, we refer to previous uses of model-based PSF subtraction with JWST and how they have never surpassed empirical reference star methods like KLIP (e.g., https://ui.adsabs.harvard.edu/abs/2024ApJ...967L...8L/abstract , https://ui.adsabs.harvard.edu/abs/2023ApJ...945..126G/abstract), and previous uses of autodiff in PSF modeling (e.g., https://ui.adsabs.harvard.edu/abs/2026A%26A...708A.311K/abstract and especially https://ui.adsabs.harvard.edu/abs/2025ITCI...11...36F/abstract) and how our work differs from them (in brief, substantial model and optimization framework changes that enable PSF subtraction is coronagraphic imaging for the first time on real, on-sky data, surpassing traditional approaches by up to an order of magnitude).**
- **Comparison with benchmark method KLIP: We have clarified the comparisons between our method FLIP and the benchmark method KLIP. In particular, in Methods (Section 2.2 "Experiments") we describe in more detail our reproduction of KLIP results in all datasets, in all cases with results closely matching the published KLIP results. We particularly clarify that the TYC 5899 dataset KLIP reduction was done with the full reference library used by the original authors, and that only for FLIP did we single-out the most similar image from this library for model pre-calibration. We have also changed our flux-to-contrast calibration of dataset TYC 5899 for all the curves shown in the TYC 5899 panel of Figure 3 (as we used the same calibration for all of them), now closely matching the results shown in Figure 16 of Bogat et al 2025 (https://ui.adsabs.harvard.edu/abs/2025AJ....170..225B/abstract).**
- **FLIP model interpretability: We have added a suite of "leave one out"/Jack-knife/ablation tests for our method FLIP, showing the role of each optimization sub-stage by performing FLIP while leaving out one sub-stage at the time. This is shown in FIgures 6 and 7, and we describe them at the end of Section 2.1.2 (Optimization design) in Methods. Importantly, these tests also show:**
    - **The source of improved performance of FLIP over KLIP: solving for star-coronagraph misalignment and OPD differences between reference star and science star allows FLIP to perform better than KLIP, while KLIP performance is limited by these differences)**
    - **The source of improvement of FLIP relative to previous autodiff PSF subtraction efforts like those from Feng et al 2025 https://ui.adsabs.harvard.edu/abs/2025ITCI...11...36F/abstract : Our model**

has more flexibility than the one presented in Feng et al 2025, which allows us to model complexities of real on-sky data that the simplified Feng+2025 model cannot (Figure 6), and our multi-stage optimization with model pre-calibration allows us to mitigate exoplanet over-subtraction (Figure 7), which Feng+2025 did not incorporate.

- **Instrument stability-limited sensitivity: We claim that standard method KLIP is limited by instrument stability (in particular, wavefront error or OPD drift between reference and science observations, and star-coronagraph misalignment variations), and that FLIP achieves sensitivity below this limit. To better support this claim, we have added a suite of experiments on simulated data to reproduce the sensitivity achieved by KLIP in the presence of these instabilities. We simulate JWST/NIRCam observations with no source of noise other than instrument instabilities. We show that the sensitivity achieved with KLIP in these simulated experiments, calibrated with injection-recovery tests, is consistent with the real KLIP sensitivity on our real observations. We also show that FLIP indeed achieves sensitivity superior to this limit. This is shown in Figure 3 in the manuscript and we describe it in the Results section of the Main manuscript. We describe our experiments in detail in Section 2.4 in Methods.**
- **Addition of Supplementary Information section: We have added a “Supplementary Information” section separate from Main and Methods in order to maintain brevity in Main and Methods. This includes “Supplementary Methods” with details on the model design, optimization framework and PSF subtraction metrics, and “Supplementary Discussion” where we discuss future work.**

Below we present a point-by-point response to all the comments provided by the reviewers.

Referees' comments:

Referee #1 (Remarks to the Author):

This paper describes the FLIP PSF subtraction algorithm, which uses a physics-based auto-differentiated model to optimally estimate and remove starlight. The results are compelling and significant, with on-sky demonstrations achieving substantially higher SNR than the current state-of-the-art. The topic is also timely, both given the application to JWST as well as the desire to achieve the photon noise limit at all separations for HWO. The methodology is sound. Most of the conclusions are robust, though several could be improved or better justified.

The paper is well-written and clear. Figures support the paper, and it generally provides adequate detail given the format desired for this journal. Additional references are needed in a few places.

Below are issues identified with the paper. Many of these (labeled “minor”) are simple points of clarification. A handful are seen as more pressing (labeled “significant”) and need to be addressed.

I recommend this paper for publication once the issues below are addressed.

Significant issues:

1. Issues with stability claims (lines 59, 98, 176): the authors allude to the potential for methods like this to relax stability requirements of HWO, claim that their results show the potential to surpass stability limits, and claim their results beat “the stability limits of JWST.” What precisely is meant by this? There is no reference for this claim, it does not seem to be discussed in detail, and I see no explicit demonstration of it. The authors must justify this and show it explicitly in their results to make such a claim. If the authors are assuming that the comparison between KLIP and FLIP with JWST data shows this, they must also show that other factors were not limiting the KLIP performance (e.g., the choice of reference star, target acq errors, etc. -- I would consider target acq errors a repeatability issue, not a stability issue, but am open to hearing otherwise).

We have added explicit calculations of the stability-limited KLIP performance, with a more detailed discussion and with added clarity throughout the text as to what is meant by this. We have explained this in the Abstract, in the caption to Figure 1, and in the details on the calculations for the stability curves presented in Figure 3.

We compute the stability-limited KLIP performance as follows. For each of the four datasets, we simulate synthetic observations that include no noise other than other than 1) the instrumental factors we refer to as “instability”, i.e., OPD drift and star-coronagraph misalignment differences between reference and science observations, and 2) the effects of algorithm over-subtraction intrinsic to KLIP. In all cases, our stability-limited KLIP performance on our simulated data is consistent with the KLIP performance on real data to better than a factor of 2, demonstrating that KLIP sensitivity is limited by telescope stability.

Star-coronagraph misalignment is partly a consequence of target acquisition errors. We include it under the umbrella of “instability” for the following reasons:

1. **Mathematically, star-coronagraph misalignment is equivalent to tip-tilt OPD aberrations. In terms of PSF subtraction limitations, these tip-tilt OPD aberrations are indistinguishable from other sources of OPD variations as far as PSF subtraction is concerned. For the most part, what limits KLIP-like PSF subtraction methods (and in general, differential imaging-based PSF subtraction) are the different states of the observatory when observing reference stars vs when observing the science target. From the point of view of an observational astronomer, the impact on planet detection sensitivity is the same.**
2. **Target acquisition precision and raw observatory stability may present different engineering challenges, but once some level of target acquisition precision and some level of observatory stability is in place, the impact on planet detection sensitivity is the same. This is related to the point above about the source of these aberrations being indistinguishable from the point of view of an observational astronomer. In other words, if our method FLIP or any variation of it can significantly reduce the component of the noise budget associated with image post-processing (as we show in the case of JWST), that noise budget can relax requirements in other parts of the observatory engineering. This is equally true for target acquisition precision requirements as it is for raw observatory stability requirements.**
3. **Finally, observatory stability and target acquisition precision are not completely monolithic and orthogonal matters. Just like many different effects can influence observatory stability (e.g., https://ui.adsabs.harvard.edu/abs/2024SPIE13092E..10T/abstract), many different effects can also influence target acquisition accuracy, some of those being precisely related instrumental instabilities. While target acquisition can colloquially be characterized as a repeatability issue, the underlying mechanisms limiting repeatability are multivaried and complex, and interrelated with other aspects of the observatory stability and state.**

2. Line 164: the authors incorrectly claim that “only model-based PSF subtraction is capable of reaching…” the noise limit. This makes many assumptions about the magnitude of the instabilities. The curves in Fig 3 show that this doesn’t happen at the majority of separations in their work. Further, there are alternative instrument designs and ConOps that, in theory, could help to greatly mitigate the speckles. This sentence should be rephrased.

Sentence rephrased (start of “Future improvements” section)

3. Issues with SNR measurement:
- The authors show much higher SNR than other reduction methods. While I agree that this almost assuredly means better performance (which is obvious

from some of the figures), SNR does not fully communicate the performance of the data reduction process. Ideally the authors would report a confidence level. As shown in Mawet et al. (2014), the confidence can still be quite low given the limited number of samples at small separations. The authors must also clearly describe how they measured that confidence level (e.g., using measured standard deviations and t-test distributions vs Monte Carlo methods)

We have added the confidence equivalent in the caption of Figure 2 using t-statistics (that is, the Mawet et al. 2014 correction). We have also explained this in Section 4.1 on the Supplementary Methods section under "PSF subtraction performance details."

- When the authors measure the SNR, they take the standard deviation of all pixels in a thin annulus as the noise and use the peak of a Gaussian fit to the planet. Presumably the peak of this Gaussian adopts the same pixel resolution? Their results would not appreciably change if they were to use the brightest pixel, correct?

Correct. We take the Gaussian to try to mitigate the effects of PSF deformation from the PSF subtraction process (the same reason why we smooth the frames before computing SNR). The value of the peak is taken in the same units as the flux density per pixel, therefore we maintain the same pixel resolution. We state this explicitly now in Section 4.1 on the Supplementary Methods section under "PSF subtraction performance details."

The results do not appreciably change if we use the brightest pixel instead of the peak of the Gaussian (all numbers in MJy/sr):

FLIP:

- **for HIP 65426 b, the peak of the Gaussian is 10.96 and the peak pixel is 10.95,**
- **for 51 Eridani b in F430M, the peak of the Gaussian is 2.41 and the peak pixel is 2.36,**
- **for 51 Eridani in F410M the peak of the Gaussian is 5.95 and the peak pixel is 5.75,**
- **for the injected planet in TYC 5899, the peak of the Gaussian is 0.51 and the peak pixel is 0.49 .**

KLIP:

- **for HIP 65426 b, the peak of the Gaussian is 13.58 and the peak pixel is 12.93,**
- **For 51 Eridani b in F410M, the peak of the Gaussian is 11.29 and the peak pixel is 11.79.**

- Do you assume you know where the planet is when measuring SNR, or are you simply selecting the location of brightest flux/best fit to a Gaussian?

We assume we know the location of the signal (though this is also the location of the highest flux). We state this explicitly in Section 4.1 on the Supplementary Methods section under "PSF subtraction performance details."

- Figures show images in terms of counts. It would also be helpful to see SNR maps. This would highlight any false positives in the images (if they exist).

SNR maps and details on their calculation have been added in the Methods (under PSF subtraction performance metrics) and in the Extended Data Figures (Figure 9).

4. A number of parameters of the reduction are "tuned" (e.g., see line 292). Line 378 states explicitly that different values were used for each data set without much of an explanation why. When tuning these parameters, presumably you are watching the planet and seeing what makes the planet "pop?" Without knowledge of what these parameters need to be ahead of time, this parameter tuning is technically part of the detection process and ideally should be captured in the confidence level. The authors should discuss this statistical issue.

We have added a discussion on this in the Supplementary Information (section 4.1, under "Optimization design implementation details."). In short, we emphasize that we choose our parameters based on injected fake planets, not directly on the real planet. Furthermore, this is a standard approach in machine learning: training a model on a set of training data before applying it to the data of interest.

We further highlight the parallels between the community's practices in parameter tuning with KLIP, and how those are accounted for in KLIP. Parameter tuning in FLIP is precisely analogous to this practice. While a more sophisticated treatment of uncertainties in choosing hyperparameters for KLIP or FLIP would be beneficial, it is outside the scope of this work to define a formal framework to do this, so we opt for practices analogous to what the community already does with KLIP.

- The authors inject planets and monitor their SNR during the model fitting process to avoid over-subtraction of the planets. Are the fake planets injected at all separations and angles? How can one be sure that maintaining the flux of an

injected planet at an alternate location in the image maintains the flux of the real planet? Was this tested?

We added a figure (Figure 8) showing the SNR for observation HIP 65426 as an illustrative example across injected planets with several separations (~0.5 arcsec to ~2.5 arcsec) and angles (spanning ~70 deg; other position angles coincide with the real companion). We show that, while there are individual differences for planets largely separated from one another, most injected planets reach a plateau in SNR around similar separations. We also describe our injections more explicitly in the “Optimization design” section of the Methods.

Importantly, this is equivalent to the standard in our community when using KLIP: in KLIP, we inject fake planets at similar separations as the planet candidate, and from the planet signal loss seen in the injected planets we obtain an estimate for the real planet candidate flux loss. This process is also used to choose an ideal number of KL modes.

Therefore, in our work, we follow the standard community practices for KLIP and adopt them for FLIP. We inject fake planets at similar separations as the real planet (when there is one), and we use that (e.g., the average signal loss in synthetic planets) to estimate signal loss for the real planet. We also use this process to choose an ideal stopping iteration. This is also a standard practice in the machine learning community, when optimizing a model on a training set and then applying it to a test set or real data.

- When tracking the signal reduction of injected planets (in order to stop the optimization process), is the optimization done at all separations simultaneously, or is each separation separately optimized in thin annuli?

Clarified this in “Optimization design implementation details” in section 4.1. We optimize the entire image simultaneously, motivated by the physics-based modeling nature of FLIP.

Minor issues:

1. Line 86: The authors reference “instantaneous pointing.” Does this account for high frequency jitter? Does JWST provide you with data to be able to include this?

We have modified the sentence to “star-coronagraph misalignment”: we do not fit high frequency jitter (which is negligible for most JWST data given pointing jitter ~1 mas; Rigby et al 2023 https://ui.adsabs.harvard.edu/abs/2023PASP..135d8001R/abstract), only

the misalignment between the star location in the field of view and the center of the coronagraph mask.

2. Line 94: This sentence is probably not technically accurate—I think the authors mean that this is the first time a physical model has revealed a planet when empirical reference observations haven't? Even with that change, I suggest that the authors rephrase this sentence to reference prior work and provide better context. Other efforts have considered model-based PSF subtraction and it would be good to acknowledge those (e.g., Lawson et al. 2024 is a recent one).

Addressed and added relevant references in the Introduction.

3. Fig 3: Why does KLIP outperform FLIP beyond 2 arcsec for TYC 5899? Why does FLIP outperform the noise floor at 1.8 arcsec for 51 Eri?

FLIP exhibits more signal loss at larger separations than KLIP in this particular target (TYC 5899). This is clarified in the last paragraph of Section 2.5.

The contrast curve being below the noise floor is purely due to fluctuations in the contrast curve and residual starlight, not due to actually having sensitivity below the noise limit at that separation.

4. Line 168: How much do these various terms limit your results?

We have expanded our discussion of these terms in the "Future improvement details" section in Supplementary Discussion.

5. Line 255: There should be a reference for the charge diffusion Gaussian kernel treatment

Added a link to the relevant STPSF page discussing this; this is the only reference available for the specific number measured for charge diffusion.

6. Line 275: The two-stage optimization is nice. Probably useful to remind the reader that the reference star is known not to host bright planets

Added this clarification.

7. Line 288: Why is the optimization more stable if you only solve for the OPD after the mask instead? Is this a numerical limitation or does this physically mean something for JWST?

Added a clarification on the text under "Optimization design" in the Methods section. It is a numerical matter: if we solve for the OPD after the mask, the OPD does not need to be propagated through the coronagraph focal plane mask.

8. Line 290: Reference for the Adam optimizer?

Added the reference.

9. Line 331: This is an interesting result. Presumably the results are not as good on the time-resolved data because you treat each individual frame as uncorrelated? If one were to treat it as a correlated data cube, presumably one could only do better (e.g., knowing that the planet has only a DC component while the speckles can vary temporally). Can these sorts of correlations be included in the fitting process?

Added a note mentioning this potential avenue under "Optimization design implementation details" in the Supplementary Methods section. For our current datasets, given the brightness of the stars and the depths of observations, the simplified treatment of assuming static speckles and applying FLIP on mean-combined data products works well.

Referee #2 (Remarks to the Author):

- Summary:

This manuscript reports new improvements in starlight suppression for coronagraphic direct imaging of exoplanets, in particular, directly improving sensitivity and detectability in archival data. The article presents a new PSF modeling approach that combines physics-based modeling of the telescope and optics with GPU-accelerated computation, machine learning, and automatic differentiation, applied to coronagraphic instruments. The authors demonstrate this new methodology on JWST/NIRCam archival data, achieving better sensitivities compared to a state-of-the-art post-processing technique, KLIP. They

also estimate the fundamental contrast limit of each JWST/NIRCam observations, and show that their method reaches sensitivities close to this limit, performing better than the other post-processing technique. In addition, they recover two known exoplanets with higher S/N and detect one known exoplanet that was not recovered in previous studies. Overall, this method shows strong potential to improve exoplanet detectability, as demonstrated by the improvement in sensitivity and speckle suppression in JWST/NIRCam data.
Two main points should be noted. First, the core idea of the method has been presented in a recent published article. While the implementation here is more advanced and applied to coronagraphic instruments, the level of novelty could be better clarified in relation to previous studies. Second, in one of the four datasets, the implementation of the "classical" post-processing appears suboptimal, which may bias and affect the comparison with the proposed method in that specific case. However, these points do not significantly impact the main results and conclusions of the manuscript, which remain robust and of great interest for the community.

- General comments:

General comment 1: It is unclear how the author processed the data: observations such as HIP65426 were carried out using two roll angles. How did the authors process the data using KLIP? For example, it is possible to combine RDI+ADI, or just RDI and de-rotated the data and combine them. Is the stack performed using mean or median?

Added clarification in the "Experiments" section of the Methods: all targets were reduced using RDI+ADI, following the choices in the published works. We mean-combine frames.

General comment 2: I am wondering if the flux is being biased using this new method. It would be great if the authors could provide the flux measurement and uncertainties of one planet (e.g., HIP65426b), and then compare this value to the published one. The authors are free to consider this comment, since it goes beyond the core of this study; however, the corroboration of flux calibration is also important, but could be investigated in future publications (e.g., the detection of 51 Eri b).

We choose to investigate this question in future work. This is partly because there are several ways to measure flux, and in particular, our physics-based model opens the door to fitting the planet as a part of the astrophysical scene in the model directly. Understanding whether this process is more appropriate than measuring the flux from the detector images after PSF subtraction requires further investigation.

General comment 3: The authors occasionally generalized the results and improvements in sensitivity to JWST as a whole (e.g., line 22/23, line 191, line 206), while the method is demonstrated using JWST/NIRCam data only. It would therefore be more appropriate to refer specifically to JWST/NIRCam. Other JWST observing modes and instruments (e.g., MIRI+4QPM) have also shown strong/great coronagraphic performance but involve different coronagraphs/optics, observational strategies, and post-processing challenges and improvements (e.g., Boccaletti et al. 2022, https://ui.adsabs.harvard.edu/abs/2022A%26A...667A.165B/abstract ; Carter et al. 2023, https://ui.adsabs.harvard.edu/abs/2023ApJ...951L..20C/abstract ; Boccaletti et al. 2024, https://ui.adsabs.harvard.edu/abs/2024A%26A...686A..33B/abstract ; Godoy et al. 2024, https://ui.adsabs.harvard.edu/abs/2024A%26A...689A.185G/abstract ; Malin et al. 2024, https://ui.adsabs.harvard.edu/abs/2024A%26A...690A.316M/abstract ; Lagrange et al. 2025, https://ui.adsabs.harvard.edu/abs/2025Natur.642..905L/abstract). Clarify this point and distinction would better reflect the findings, impact, and scope of this study. I also suggest to mention the incorporation of JWST/MIRI in the method and public codes as a future work, if this is part of the short-term plan of the authors.

Clarified this point and added mentions to the potential application for MIRI (both in Discussion and Supplementary Discussion). We have reformulated JWST to JWST/NIRCam in most of the text.

- Major comments:

Line 19/20/21: "The key innovation that makes this feasible is a differentiable optical model enabled by modern machine learning frameworks with automatic differentiation …" I am wondering about this statement, since the "innovation" is not longer applicable. A recent article accepted in Astronomy and Astrophysics (Kuznetsov et al. 2026, accepted 19 February 2026,

https://ui.adsabs.harvard.edu/abs/2026arXiv260323339K/abstract) reported the successful modeling of PSF using physics-based, GPU-accelerated, with differentiable optical model coupled with neural network and automatic differentiation. Since the mentioned study is focus on ground-based telescopes, and only in “classical” PSF, I would re-phrase the sentence to focus more in space-based telescopes and, directly, coronographic stellar PSF, which is the main core of this article. I present more comments about this point in the “line-to-line comments”. I want to emphasise that the result reported here has an impact in exoplanet direct imaging, and it is the first time that this method is implemented in coronographic-PSF instrumental modeling, therefore this must be highlighted accordingly throughout the entire manuscript.

We agree with this, and we have clarified this point in the introduction, discussion, and methods sections. We include explicit reference to this work in particular and we discuss the differences in the Discussion and Supplementary Discussion section.

Line 373-375: I am wondering about the approach the authors used here. The simplification for “apples-to-apples” comparison is well far from what the community is doing. I would like first give some context: reference stars are observed as close as possible in time to avoid as much as possible the wavefront and thermal drift variation and, in most of the cases for space-based telescopes, it is adopted the so-called dither-patter. This dithering is created to introduce more diversity in the PSF library and capture spacial variation due to an imperfect centering/pointing. As far as I know, the self-reference method adopted in different surveys used the fact that they have a large library composed by stars observed in different times with different pointing/centering. This generates a PSF library rich in diversity, which mimic the classical observation of a reference star. Some studies such as Xie et al. 2022 (https://ui.adsabs.harvard.edu/abs/2022A%26A...666A..32X/abstract) shows the efficiency of using these libraries to reach a better performance in sensitivity and speckle removal.
The approach adopted by the authors, by definition, will reach the poorest/worst contrast/sensitivity using KLIP or any other method. This is because the diversity in both dithering and speckle variation is not captured by a single observation (which is separated by a significant amount of time and, therefore, considerable difference obs conditions, and different pointing error). This generates a considerable mismatch between the reference star and the science target. Even for space-based telescopes with an unprecedented stability such as JWST, the

differences due to thermal and wavefront drift and pointing errors can generate significant mismatches.
The authors can check this by using the same approach with the HIP65426, by using a different star wide separated in time, and using only one of the dither patterns. The authors will note a degradation in sensitivity and ability to suppress the speckles. This point need to be taking with caution.
In conclusion, the approach adopted here produces the worst performance in contrast for the KLIP. Therefore, the authors cannot compare a contrast don't use by the community, which generates the poorest performance, with their method.
It is also important to highlight that in Bogat et al. 2025 (paper cited by the authors for TYC5899), the contrast reached by the survey using the self-reference library, is about ~3e-6 at ~2.5 arcsec, an order of magnitude deeper than the one reported here (~1e-5 at ~2.5 arcsec). Also ~3e-4 at ~0.6'' vs 1e-3 at ~0.5'' reported here.
I want to highlight that the idea of using a single star as reference in surveys is a good proof for the method described here (FLIP), which demonstrates that it is not necessary to have a close-in-time reference star observation (and non dithering patter) to reach a good performance. This can be of great interest for the community for planning future observations, and a motivation to explore the archive data.

We have added clarification for our methods. In our original submission and in this submission, we used the "full PSF library" used also in Bogat et al (2025), reproducing the published result just as was done with the other datasets. The choice of a single reference star out of the full PSF library was meant to refer only to the star being used for calibration of our FLIP model (stage 1). As can be seen in Figure 14 of Bogat et al (2025), the residuals we present in our Figure 2 closely match the residuals the authors present for TYC 5899.

The difference in the contrast curves is due to two factors.

1. **First, after discussing with the lead authors of Bogat et al. (2025), it came to our attention that we used a different flux calibration to the one presented in their study. That is, a different conversion factor from flux density to contrast units, which is done by simulating a non-coronagraphic PSF model using the target star spectrum. We utilized a stellar spectrum slightly different from the one used in Bogat et at. (2025). We have now used a contrast conversion in line with the published result, so our contrast units are consistent. As a result, our three contrast curves (including the fundamental noise limit curve) have been properly scaled, without changing the relative results, since the same flux-to-contrast**

conversion factor is used for all three. The relative differences between KLIP, FLIP and the noise limit remain therefore unchanged.

2. **Secondly, the contrast curves presented in Figure 15 of Bogat et al. (2025) were computed including the first epoch observation of TYC 5899. In Bogat et al. (2025), they explain that TYC 5899 was originally observed close-in-time to the full self-referenced survey, but the second roll observation failed. As a result, TYC 5899 was re-observed months later with two rolls. This second epoch is the one we reduce in our work. Our choice of this second epoch is intentional, as, contrary to the other three examples in our Figure 2, it is a valuable test case for data where high quality, near contemporaneous reference observations (including with dithers) may not be available. Importantly, this separation in time harms KLIP performance because of the OPD variations in long timescales. As such, this example on the second epoch of TYC 5899 also is a good example of our method FLIP performing better than KLIP given the OPD variation/long-term observatory stability. Consequently, we do not include the first epoch data of TYC 5899 in our analysis. Nonetheless, our final contrast curve is close to the one presented by Bogat et al (2025): we have added an explicit comparison in Section 2.2 of Methods ("Experiments").**

We have added clarification on these points in the Methods section when discussing the data reduction of this dataset.

- Minor/medium highlighted comments:

Line 406-407: "We generate a random Gaussian sample with mean 0 and σ equal to the total noise level in each pixel" It is not entirely clear how the authors propagate the uncertainties here. The ERR contains all the noise contributions, and exists one per integration. In the case of KLIP, it is not trivial to know how this is propagated through the pipeline. In the most simplistic scenario, the ERR of the final stacked image is the quadratic sum of all the ERR from each integration. The contrast curve then can be calculated using the FPF with the mean value being the noise level. It is unclear why the authors used Gaussian centred at 0 and using sigma = ERR to generate a "random Gaussian sample". This generates new images with negative values, which is conceptually wrong when talking about noise/uncertainties. Following the article cited by the authors (Godoy et al. 2024), the noise propagation looks similar to the case 0 presented in that study. As is mentioned in that study, there are some assumptions that have been done and need to be mentioned explicitly here. For instance, the non-correlated noise between nearby pixels. If the noise propagation was done following the ideas presented in that article, please cite it accordingly.

Since the fundamental sensitivity limit is one of the core ideas in this article, I encourage the authors to explain the procedure more clearly and in detail, or cite directly the mentioned article and provide a slightly more extended summary about the procedure.

We have expanded our discussion of the fundamental noise limit in the Methods section under "PSF Subtraction metrics". In short, we do use the "case 0" noise propagation used in Godoy et al. 2024 and we have added the clarification and an explicit citation. The other cases in Godoy et al 2024 are appropriate when we account for noise not only in the science observation, but also noise in the reference observations being used for KLIP PSF subtraction. In the case of model-based PSF subtraction, the PSF model does not contribute fundamental noise since it has no photon noise, read noise or flat noise associated with it. In other words, our fundamental curve is the *actually fundamental* curve assuming only the science image contributes noise to the PSF subtraction, as is noted in Godoy et al 2024. We make this choice for consistency with model-based PSF subtraction and to show an upper limit of achievable contrast, independent of the reference stars used for KLIP (i.e., incorporating noise from reference observations can only make the fundamental noise limit shallower, not deeper).

On the point about our noise sample having negative values, it is expected for our residuals to have negative numbers. This is because we are simulated "post-PSF subtraction" noise, assuming that a perfect PSF model has been subtracted off and so random fluctuations are expected to be both positive and negative. This is mathematically equivalent to generating a Gaussian using the value of the ground truth PSF as the mean, and then subtracting off the ground truth PSF. This is in the same way that KLIP subtractions have negative numbers, since slight noise from the science image or model (from forcing a zero mean, as is done with KLIP) can have this effect. We have clarified this in Section 2.3 as well ("PSF subtraction metrics" in Methods) with the statement "That is, when generating our noise realization we are simulating what the image would look like after perfect PSF subtraction. "

Line 425/426: "While this may not be optimal for exoplanet recovery given the differences in …". It is not clear if the authors did this just to show the figures, or to quantify the S/N as well. In the case of S/N, it is not clear if the authors optimize the extraction of the S/N for FLIP and KLIP, or just keep one single configuration for the extraction in the entire FoV. It is not a guarantee that using the same KL modes the authors can obtain the best S/N for KLIP. This is a bias

when comparing both methods, and need to be clarified. Also, are the chosen number of iterations the optimal ones, or the most pessimistic ones?

We clarified this point at the end of “Methods: TYC 5899 synthetic injections”. We emphasize that in those injections, we keep the method hyperparameters fixed (annuli and KL modes for KLIP, learning rates and iterations for FLIP) to visually show the sensitivity of the resulting observation in the presence of a variety of planets. This is a visual complement to the contrast curves shown in Figure 3, and highlights that our method hyperparameters are not over-tuned for performance in only a single, small sub-region of the image.

Line 434: “… and it still causes some planet signal loss.”. I am wondering about this statement. Are the authors suggesting that the throughput correction will not be needed at some point using this method?. From what the authors mentioned throughout the article, this technique is limited by several factors that are not even possible to control or model. And as any other technique, the model of the PSF will never be absolutely perfect and, therefore, it requires a throughput correction to account for flux loss. The throughput can be minimized as much as possible, but still needed to account for any type of biases. I strongly suggest the authors to modify this sentence. Also, please provide the throughput correction for the FLIP and KLIP for the 4 datasets presented in this study. This will better support the statement here, and will provide how better is the modeling of the PSF.

We removed the statement about causing planet signal loss, since the point about reaching the fundamental noise limit is more representative of the future potential of FLIP without implying bias corrections will not be needed.

We have included a plot showing several injected planets with their respective throughput correction (signal loss) and SNR as a function of iteration for the HIP 65426 dataset (Figure 8, in Extended Data). This plot was made to address concerns about sensitivity to choices of injected planets but also addresses the point here about throughput correction.

The throughput correction/amount of signal loss has been added in Section 2.3 (“PSF subtraction metrics” in Methods). We show the range of values for injected planets in the vicinity of the planet whose SNR we report in Figure 2, for brevity. The overall throughput correction for all datasets is accounted for in the contrast curve calculation for both KLIP

and FLIP (Figure 3); the quality of the PSF modeling is determined by both the suppression of speckles and the planet throughput, both factors are accounted for in the contrast curves.

- Minor comments - line-to-line comments:

Line 5: correct the citation:
https://ui.adsabs.harvard.edu/abs/2023ASPC..534..799C/abstract

Changed the citation.

Line 8/9: “While advances in coronagraphy and telescope stability are increasing the sensitivity of our observations, …” I suggest to point directly to space-based telescopes, since this is the focus of the article.

Changed to “telescope stability in space-based observatories”

Line 22/23: “We demonstrate our method on observations from the James Webb Space Telescope (JWST)”. Please, be specific here since this study does not explore all the coronographic modes of the JWST (e.g. MIRI).
Suggestion: “We demonstrate our method on observations from the James Webb Space Telescope (JWST), Near Infrared Camera (NIRCam) coronographic mode.”
Also, provide the proper citation: JWST - Gardner et al. 2006
https://ui.adsabs.harvard.edu/abs/2006SSRv..123..485G/abstract ; NIRCam Rieke et al. 2005
https://ui.adsabs.harvard.edu/abs/2005AAS...20711509R/abstract, Krist et al. 2009 https://ui.adsabs.harvard.edu/abs/2009SPIE.7440E..0WK/abstract

Changed to the suggested phrasing and added citations.

Line 23/24: “We recover previously undetected planets and improve detections of known planets with sensitivity ...” Here, I recommend to highlight that these are known exoplanets, otherwise could be interpreted as new discoveries rather than improvements in the data processing. Also, in this study it was recovered just

one know (exo)planet that was previously undetected, so I recommend to place the statement in singular.
Suggestion: “We recover previously undetected known planet and improve the detections of those known recovered planets with sensitivity …”

Sentence rephrased.

Line 23: I strongly suggest mentioning the detection of 51 Eri b since this provides strong support to the technique, the real impact on detectability and how much the sensitivity was improved.

We added this point.

Line 39: please provide a reference here (e.g., Currie et al. 2023, https://ui.adsabs.harvard.edu/abs/2023ASPC..534..799C/abstract)

We added this reference.

Line 43: Since for ground-based telescopes there are more factors than the ones mentioned here, I suggest to refer directly to space-based telescopes.
Suggestion: “... and instrumental aberrations when observing with space telescopes.”

We reworded it as “strong dependence on factors such as stellar properties and instrumental aberrations”. We make clear the focus on space telescopes on the next paragraph.

Line 49: “… are fundamentally limited by instrument instabilities, such as telescope pointing errors and thermal drifts.” I suggest to be more explicit here, focusing on the space telescopes. Otherwise it should be mention, for example, turbulence.
Suggestion: “… and thermal drifts on space telescopes.”

We reworded the first two sentences of the paragraph, emphasizing that those factors are the main contributors on space-based telescopes.

Line 91: Following my suggestion in Line 22/23, here should be: “from the JWST/NIRCam”

Changed.

Line 91/92/93: “FLIP produces better planet detections than standard methods in published datasets, and in some cases it reveals images of planets that had remained undetected by traditional techniques.” The sentence is too strong to respect the results presented in this work. Claim for “better planet detections” and “reveals images of planets that had remained undetected” is more linked to a successful detection of new planets (that are possible only with this technique), than to an improvement on sensitivity. What the authors should highlight here is the improvement in sensitivity/detectability and recovery of know planets.
This sentence must be re-phrased.
My suggestion: “FLIP improves sensitivity and signal-to-noise ratio relative to standard methods, enhancing planet detectability and enabling the recovery of a known planet not detected by traditional techniques.”

Rephrased the sentence to emphasize the detection sensitivity and higher SNR, and the first recovery of 51 Eri b at 4.3 micron.

Line 94/95/96: “After over thirty five years of repeated community efforts, this work is the first time that exoplanets are revealed in coronagraphic imaging data using a physical model instead of empirical reference observations.”. The part of “of repeated” sounds too strong here. I would suggest a more formal phrase, highlighting both the results and the community effort.
Suggestion: “After over thirty-five years of community-wide efforts, this work is the first time that exoplanets are detected in coronagraphic imaging data using a physical model instead of empirical reference observations.”

We have rephrased this to “After over thirty five years of community-wide efforts, this work is the first time that model-based PSF subtraction surpasses standard techniques in coronagraphic exoplanet imaging, with up to an order of magnitude improvement in sensitivity. ”.

Line 102/103/104/105: Since the recent published article (Kuznetsova et al. 2026, accepted 19 February 2026, https://ui.adsabs.harvard.edu/abs/2026arXiv260323339K/abstract), use the same principle based on physics-based modeling and automatic differentiation, I strongly suggest to the authors to re-phrase this paragraph, since the "innovation" here is not longer applicable in the way it is presented. Please re-phrase it being sure the proper references appears clearly here.
I have two suggestions:
1) "… consists of two primary methodological innovations, building on previous work (cite the paper here) and adapted here to space-based coronagraphic instruments: ..."
2) "… consists of two main methodological advances (cite the paper here), here applied to space-based coronagraphic instruments: …"
N.B.: the authors can claim for the novelty in the application to coronographic PSF reconstruction, but giving credits to previous and recent works done in the PSF modeling in general.

Rephrased (similar to the second suggestion) and added the reference.

Line 107: "… real JWST data." Since here the authors present the model for one observing mode, I strongly suggest to be more precise.
Suggestion: "… real JWST/NIRCam coronagraphy data."

Rephrased.

Line 119: Authors could add "… close in time and at a small angular separation …"

We have rephrased this paragraph for brevity.

Line 122: "… by injecting synthetic planets close to the sensitivity limit and tracking the signal-to-noise ratio (SNR) of the fake planet detection.". I have several questions about this sentence: 1) It is not entirely clear how the authors obtain the contrast curve (sensitivity) to be able to inject the point-sources. Is it obtained from state-of-the-art post-processing techniques? Or is it coming from a really rough first iteration of the method? 2) What do the authors mean by "close

to the sensitivity limit”? Is it a 3-sigma, 5-sigma, or 10-sigma? Please be more precise when providing this type of information. 3) how many point-sources are injected per annulus, and how this number impact the performance in terms of sensitivity and computational time? I also recommend that the authors to change “synthetic planet” by “point source”, or use “injected fake planet” instead.

We have added more details on this section in the Methods section at the end of the implementation details of the “Optimization Design” section. We note that, just like with KLIP, there are no general strict best practices on fake planet injections. Just like with KLIP, our PSF subtraction is not highly sensitive to the choices of these injections. We have further emphasized this with a plot showing several planet injections and their associated SNRs and signal losses. Point by point:

1) **Our sensitivity curve here refers to the fundamental 5 sigma sensitivity curve from the noise limit.**
2) **We inject planets initially at 4-20 times the 5 sigma fundamental contrast curve. If the planet is too faint after a first subtraction (e.g., there is significant signal loss), we might inject it a bit brighter. We have emphasized this in the Methods section, to keep brevity in the Main section. The choice of how bright is the right about of bright is a practical question, and it may vary depending on scientific context.**
3) **We inject 1-3 planets per annulus, and in our field of view we use 5-7 annuli. We have also added this in the Methods section. These choices are context dependent (for instance, when using the LWBAR mask a smaller number of planets per annulus is possible, since only the field of view perpendicular to the bar is of scientific interest, and the mode has been used for characterization as opposed to planet discovery). We inject each planet individually per FLIP run, so the total computation time needed to obtain contrast curves scales linearly with the number of planets injected, but does not affect the computation time of any given FLIP run itself. Our plot showing an example of planet injections shows that our subtraction is not highly sensitive to these choices, and given the dependence on scientific context, we make no claims as to the optimal number of injections or general guidelines beyond those already considered for KLIP-like methods by the community.**

Reworded “injecting synthetic planets” for “injecting fake planets” instead.

Lines 130/131/132: When referring to the observations of the 3 stars HIP65426, 51Eir, and TYC5899, please mention the filters explicitly, since this is not something obvious. For example, Carter et al. observed HIP65426 with different filters, modes, and instruments.

Added; we also added the coronagraph modes used.

Line 138: Can the authors provide numbers more than just “robust detection”? Is the detection at 5- or 20-sigma level? What is the FPF or the metric used to define it as “robust detection”?

Rephrased as “SNR=6.3 detection”

Line 141: “…, we inject fake planets on both data sets to illustrate the …”. Can the authors explain what are these two data sets they are referring to? Is it two roll angles or two different epochs? Or two different filters?

Rephrased for clarity: the planet injections were done on the same dataset, which was then processed with both methods: KLIP and FLIP.

Line 146: I suggest that the authors also mention the S/N of the detected planet. This is a major proof of the method presented here, and demonstrates the direct impact on the direct imaging campaigns. The authors should highlight this more throughout the article.

Added.

Line 175/176: “our technique FLIP achieves exoplanet detection sensitivity beyond the stability limit of JWST”. This can be interpreted as FLIP can go deeper than instrumental limits, or that the stability limit is not actually a limit. What the authors probably want to say here is that this technique can mitigate better the limitations set by the PSF variability and wavefront evolution. Please re-phrase this sentence.
Suggestion: “Our technique achieves detection sensitivities beyond those reached by standard methods, effectively mitigating limitations imposed by JWST stability.”

Rephrased as suggested.

Line 180: As I mentioned in previous comments, I encourage the authors to be more precise with the information provided in the text. As the method is demonstrated using JWST/NIRCam observations, it would be more precise to refer specifically to JWST/NIRCam rather than JWST in general.
Suggestion: “… planet search surveys with JWST/NIRCam. …”

Rephrased as suggested.

Line 190: “FLIP could therefore be instrumental to unlocking the true sensitivity of JWST to characterize the atmospheres of the Gaia planets.”. The term “true sensitivity” is too strong, as it implies a unique, physically defined limit (limit equal to the sensitivity reached by FLP), whereas the method improves the achievable sensitivity. This phrase must be re-written.
Suggestion: “… unlocking the best performance in sensitivity of the JWST/NIRCam to …”

Rephrased closely to the suggestion.

Line 198: I suggest mentioning the recent article published in A&A (see my comments above), and highlighting the main different aspect from that study. For instance, the authors suggest the use of optics telemetry, while – as far as understood – the other article did not.

Rephrased to acknowledge the work in that paper and how it relates and differs with future directions of FLIP.

Line 199: “… telemetry is stored and made publicly available.”. I am wondering if the authors mean here that the method is not applicable to archival data but to future observations. If this is not the case, I suggest to rephrase to highlight that other options are possible as well.

Rephrased to emphasize future instruments

Lines 194-202: I suggest to mention the instruments, modes and wavelength that would be benefit the most from applying FLIP. For example, are the authors expect that FLIP generates major impacts at shorter wavelengths (H band) or longer wavelengths (L-band, N-band)? Also, it could be worth mentioning other sources that can greatly affect or challenge the implementation of FLIP in ground-based telescopes. For instance, the wind speed effect (e.g., in the VLT Cantalloube et al. 2020, https://ui.adsabs.harvard.edu/abs/2020A%26A...638A..98C/abstract), or petal-effect (e.g., Wang et al. 2024,

https://ui.adsabs.harvard.edu/abs/2024SPIE13097E..2FW/abstract ; Levraud et al. 2024, https://ui.adsabs.harvard.edu/abs/2024SPIE13097E..2EL/abstract).

Added a note that shorter wavelengths are more likely to benefit from this in a typical observation, since longer wavelengths are usually limited by thermal background rather than starlight subtraction in ground-based observatories. We add mentions of other factors in the Supplemental Discussion section under "Future improvements details".

Line 206: " … shown for JWST, however, …". Following my previous comments, this should be re-written.
Suggestion: "… shown for JWST/NIRCam, however, …"

Changed.

Line 206-208: "In this work we have shown for JWST, however, that model-based PSF subtraction can model these very instabilities and achieve contrasts better than the limit set by the thermal and mechanical drifts of the telescope.". This phrase could suggest that the FLIP method can reach sensitivities better than the physical telescope limits. The authors probably want to highlight that the FLIP method can mitigate the thermal variation and wavefront drift, which greatly affect current state-of-the-art post-processing techniques. I strongly recommend to re-write this sentence.
Suggestion: "In this work we have shown for JWST/NIRCam coronagraph, however, that model-based PSF subtraction can account for these instabilities and achieve contrasts beyond those reached by current methods, which are effectively limited by thermal and mechanical drifts of the telescope."

Rephrased the sentence and the following sentence to be clearer about the role our method may potentially play in the HWO design and performance.

Line 345/246: "…, the OPD measurement closest to the time of observation …". I am more curious to know if the authors test with different OPDs near and far in time. It is known that the OPD changes on time, and that the closest one is not necessarily the one that best represents the observation. This is because it could happens specific events that generates larger differences between two consecutive OPDs (see for example, the WFE evolution: https://jwst-docs.stsci.edu/jwst-observatory-characteristics-and-performance/optics-performance-and-stability#gsc.tab=0). As I understood from this work, the OPD represent a starting point in the modeling, so an OPD that is less

representative of the observations should 1) produce a wrong model and therefore poor performance of FLIP, or 2) the method just need more time to converge to the best solution and performance. Can the authors clarify this? This is my pure curiosity; however, it can have a really high impact on the PSF-modeling for archive, current, and future programs with JWST/NIRCam.

We have not systematically tested using non-contemporaneous OPD measurements. Anecdotally, it may take longer to optimize during the calibration stage on the reference star. Future work could investigate this in detail.

One example that may be relevant to this is the experiment on TYC 5899: here, the reference star observations were carried out ~4 months before the science sequence (and even the two rolls of the science sequence were spaced out by several days; see Bogat et al 2025 for details). Here we initialize our model with the closest-in-time OPD to the reference observation, as opposed to the science observation (this is also clarified in Implementation details under section 2.1.2). As a result, after our stage 1 optimization (calibrating the model on the reference star), our calibrated model is calibrated to a far-in-time OPD measurement relative to the science observation. While we have not investigated this in detail, this may be a reason why the contrast curves (Figure 2) show that the FLIP sensitivity is further away from the fundamental noise limit compared to how close FLIP is to this limit in the HIP 65426 and 51 Eri b datasets. Even in this case, we obtain a sensitivity superior to KLIP by almost an order of magnitude at close separations. Future work could investigate optimal choices for OPD initialization, or modifications to the method where one can reduce even more the impact of this mismatch in OPDs. An example could be where one "star hops" between optimizing the model on the reference star and the science star using the respective closest in time OPD measurements, but this and other strategies can be investigated in the future in detail.

Line 261: "… , but this formulation showed the best results.". Can the authors be more precise? What is considered "best result"? What metric was used to conclude that? And how much better in the frame of that metric? (e.g., 2 times better in the rms, or 5-sgima deeper in sensitivity performance).

We have now removed the claim about "best results" and rephrased it: we choose a "pixel grid" parametrization in order to have a flexible OPD model (change in "Model design implementation details" of Section 4.1). Future work can explore alternative parametrizations in a systematic way.

Line 316: The observations of the reference star are usually done using a dither-patter to increase the diversity on the PSF-library by capturing different PSFs. Are the authors using all the dither observations to generate the best

calibration? In the case the authors are keeping just one, how was this one selected? And what is the impact of choosing one of the other dither observations? For instance, Carter et al. used a 9-dither-patter, which means 9 reference star observations. Are the OPD solutions and stellar flux the same between all the reference star observations? I suggest the authors to show these stellar fluxes as part of the validation of the technique.

At the moment, we are using only one reference observation (one dither) as a calibration stage, since that was found to be sufficient for superior contrast performance relative to KLIP. We have clarified this in the "Choice of reference stars" section of Methods (section 2.2.2) We do not systematically investigate the differences of using different dithers.

We discuss in the Supplementary Discussion section (Section 4.2.1) that future work will be done to constrain our method to physically realistic solutions with associated uncertainties. Right now, the flexibility in our model that allows us to overcome model mismatch also means that our OPD solutions are not guaranteed to be physically sensible.

Given the large set of experiments that can be done on the retrieval of these physical parameters, we decide to explore these in future work. Future work may indeed use all dithers to obtain superior calibrations leveraging the diversity of star-coronagraph misalignment, or use many targets from a self-referencing survey in an analogous way to show similarities in the OPD solutions.

Line 325: How may injected fake planets are used in the optimization? And are these injected in the entire FoV, or just in regions dominated by the stellar chronographic PSF? How much does affects the choice of the flux in the optimization? For instance, a S/N of 5, or 20, or 100 can produce the same final performance, or generate really different results? And in terms of computing time, is it faster to use higher S/N?

We have added some remarks in the Methods section ("Implementation details" of Section 2.1.2) and Figure 8 addressing this. Starting from the 5 sigma fundamental noise contrast curve as a baseline, in Figure 8 we inject planets at several separations and position angles in HIP 65426 b at 20 and 10 times the contrast curve. We see small differences in signal loss across both panels, but generally the peak SNR is achieved at similar iterations, and as a result they produce similar PSF subtraction when we run FLIP without injected planets (e.g., Figure 2). Similarly, given that the iterations with the peak SNR is similar, these choices do not significantly impact the computation time, as it mostly depends on the magnitude of the loss function (dominated by the stellar PSF).

Line 332/333: The authors mean they used the mean-combined frames? If yes, what is the tolerance to do this in terms of performance? For instance, combine frames over 5min or 1h can produce significant differences?

We do not perform a systematic test on the tolerance for mean-combining frames. Intuitively, for very long, very deep observations (e.g.,https://ui.adsabs.harvard.edu/abs/2025ApJ...989L..23S/abstract), the OPD variations (https://ui.adsabs.harvard.edu/abs/2024SPIE13092E..10T/abstract) may become important. We choose the mean-combining approach on the entire sequences in this work for simplicity. We defer this investigation to future work.

Line 336/337: Related to my previous comment: did the authors check the integration time of the observations? The conclusion that time-average the observations could be correlated with the integration time rather than the average itself.

We added the note that the benefit likely comes from higher SNR on the PSF, which likely comes from longer effective integration time from mean-combining ("Optimization design implementation details" section of Section 4.1).

Line 340: Please mention which filter explicitly.

Added; also added in the main text.

Line 341: "We run standard PSF subtraction method KLIP". Related to one of my previous comments: is the pipeline run using RDI or RDI+ADI? Are the authors using both roll angles? I encourage the authors to be more precise with this type of information.

Added the clarification explicitly in the "Experiments" section (Section 2.2) in the Methods. We also emphasize we replicate the KLIP parameters and settings as were optimized by the original authors.

Line 352: Please provide the pipeline version used here, as well as the version used of the different tools associated to the data reduction and post-processing, in order to be able to reproduce the results.

Added.

Line 382: Regarding the S/N (and related to previous comments): how is the S/N of the injected point source impacting this value (11000 iterations)? For example, a source injected with a S/N of 3 will require the same iteration as one with S/N of 30? Also, and as I mentioned before, it is not entirely clear how the authors compute the first contrast/sensitivity to be able to inject sources that can be detected at a certain S/N.

We address this at the end of "Optimization details" under Optimization design (section 2.1.2) in the Methods. We also highlight this in Figure 8. We do not find a strong dependence on the number of iterations required for our stopping condition depending on the location or flux of the injected planet.

In the same paragraph we describe how we decide the initial flux to inject the fake planets at, related to the fundamental noise limit.

Line 392/393: Since the pipelines (JWST, spaceKLIP) have considerable improvements from, for example, Carter et al., it is important that authors explain why they used the same parameters and not an optimization to obtain the best sensitivity performance. Also, I encourage the authors to explicitly mention the pipelines and tools versions used in this study, and the ones used in the respective publications. Regarding one of my comments about TYC5899, the sentence must be re-phrased since the contrast obtained is largely in disagreement with the one reported in the literature.

Added a clarification about this in the "Experiments" section (Section 2.2) in the Methods. We choose to use the parameters used by the original authors to replicate what they considered best performance after extensive testing. Similarly extensive testing for KLIP is outside the scope of our work, and using different parameters from the original authors is unlikely to significantly improve performance, given that it is in the interest of the original authors to produce the best possible sensitivity in their own observations. Furthermore, to our knowledge there has not been systematic testing on the sensitivity improvements from recent JWST or spaceKLIP pipelines relative to earlier versions in published works.

We have added the versions of spaceKLIP and the JWST pipeline used in this work.

Regarding the comment about TYC 5899, see comment above (response to the comment about line 373-375). We have added a note to clarify this in the text both in this section.

Line 405: “… which track the combined total uncertainty in each pixel value from read noise and Poisson noise.”. The authors forgot to include the Flat noise here. Please add it.

Added.

Line 418: “… we inject synthetic planets …”. I suggest the authors to use the nomenclature from the articles and the community. In this case, “inject fake planets”, or “inject point sources” should be more appropriate. The “synthetic planets” sounds more to an injection of planets following a evolutionary track or atmospheric models from which the flux at that bandpass was extracted (which is not the case of the procedure followed in this study).

Changed.

Line 421: “… we inject a planet with flux equal to 80 times the …”. Can the authors justify why the choose of 80 and not 100 or 20? Is this value related to a better performance of the method, or to reach a reasonable S/N in both KLIP and FLIP? Or is it just linked to the best compromise between performance and computational time (iterations)?

Clarified (Section 2.5): The original choice of 80 times was arbitrary as it was found to produce planets relatively close to the detection limit. We have re-made the figures using injected planets 100 times the fundamental noise limit. We have also rephrased it as injecting flux “20 times the 5 sigma fundamental noise limit” for clarity.

Line 432/433: “While our experiments show significant improvements relative to standard techniques on a variety of …”. I strongly recommend the authors to keep the findings and conclusions within the results presented here. There are several “standard techniques” (that should be mentioned as state-of-the-art techniques) – for example ANDROMEDA (Cantalloube et al. 2015), PCA (Soummer et al. 2012), NMF, (Ren et al. 2018), LLSG (Gomez Gonzalez et al. 2016), PACO (Flasseur et al. 2018,2021), TRAP (Samland t al. 2021) – that are not studied non investigated here.
Suggestion: “… relative to the state-of-the-art technique KLIP ...”

Changed.

Line 453: I suggest the authors to refer directly to JWST/NIRCam instead of just JWST.

Changed

Line 470-448: I encourage the authors to cite the already mentioned recent paper (Kuznetsov et al 2026, https://ui.adsabs.harvard.edu/abs/2026arXiv260323339K/abstract) and to compare the ideas, challenges, and solutions between this paper and the authors study here.

Included the discussion comparing to Kuznetsov et al 2026 in the third paragraph of the Discussion in the Main text (section 1.4) and in the Future Improvements details section (Section 4.2.1)

Line 431-488: If the authors are planning to incorporate other observing modes, strategies and/or instruments such as JWST/MIRI, I suggest to include them in this section.

Added.

- Figures and tables:

Fig. 2: Please change the label of the color-bar “Counts” by “MJy/sr” or “Counts [MJy/sr]”

Changed.

Fig. 3: For TYC5899 the contrast is notably worse than the one reported in the respective article. I strongly suggest using the published contrast curve instead of the one computed here.

Addressed; see the comment above on the reason why these curves differed initially (we use an improved contrast to flux calibration now, which scales all contrast curves in absolute contrast units but does not change the relative differences between each one).

Fig. 5: Please provide in the color-axis the respective units. Improve the figure quality as well.

Addressed.

Fig. 6: Can the authors explain each panel for a more comprehensive view? Also, please all the finding and interpretations about the figure, must be move to the main text. In addition, please incorporate the color-bar units (e.g., W/m2/microns, Jy/sr, electron counts, arbitrary units).

We have explained each panel in more detail in the figure caption. We have also added color bar units.

Since the learning rate is only one of the hyperparameters explored in the Methods, and the Main section is focused on the scientific results and implications, we think that this figure and its interpretation is better placed in the Methods section along with the other hyperparameters explored in the Methods (e.g., numbers of iterations in Figure 8, stages of optimization in Figures 5,6 and 7, etc.)

Fig. 7: "The nearby off-axis bright star is also visible.". Can the authors indicate explicitly where this bright star is (even if this is obvious)?

Added in the caption to Figure 11.

Fig. 8: Is the color-scale and limits the same as for Fig. 7? Since the idea is to show if the injected point-sources are visible or not, I suggest that this figure has its own color-scale and limits.

We have changed the color scale in the figure to enhance visibility.

Table 1: caption "… PSF with very high accuracy …". This following one of my previous comments: can the authors provide quantitatively what they mean with "high accuracy"?

Added that what we mean is these parameters are sufficient to provide the sensitivity shown in Figures 2 and 3 in the Main text.

Referee #3 (Remarks to the Author):

A. Summary of key results

The manuscript presents a carefully executed and technically solid study applying a data analysis pipeline called FLIP to the detection and

characterization of planetary signals using an automatic-differentiation-based optimization framework. The work is clearly written, and the methodology is well structured and clearly described, The approach shows a meaningful performance improvement over existing techniques as it can be seen on the detected planet SNR improvement and will likely be of interest to specialists in the field.

The authors have made a solid effort to achieve improved contrast levels through their post-processing pipeline, and the manuscript is clearly written and technically competent. However, I believe there are significant shortcomings in the current form of the work. I outline my most important concerns below.

In its current form, the central contribution is not sufficiently clear or compelling at that level, both in terms of methodological novelty and demonstrated scientific impact. A recurring concern throughout the manuscript is that several key claims appear stronger than what is directly supported by the analysis presented, making it difficult to assess the true level of advance.

B. Originality and significance

From a methodological perspective, the approach is best understood as a combination of established components rather than a fundamentally new framework. It relies on physics-based forward modeling of instrumental effects and gradient-based optimization techniques, including automatic differentiation. Both elements are well established in the literature for inverse problems in computational imaging and astronomical data analysis. In addition, differentiable forward modeling frameworks of this type have been introduced in prior work and form the basis of the present implementation (as referenced in the manuscript).

Thus, while the approach appears to improve performance relative to the current state of the art, it is not yet convincingly demonstrated how this improvement relates to prior implementations of the same underlying methodological framework. In particular, it remains unclear whether the reported gains reflect a genuine step-change in capability or an incremental improvement arising from implementation choices, tuning, or application-specific adaptations. A more explicit, quantitative, and controlled comparison to prior implementations of this

approach would be necessary to establish what is genuinely new in this work and to justify the implied level of methodological advance.
In this context, the manuscript appears to extend and apply an existing differentiable modeling and optimization framework rather than introduce a new methodological paradigm. Automatic differentiation, in particular, is a standard computational tool (implemented in widely used machine learning libraries such as PyTorch) and does not constitute a novel contribution. The main novelty therefore appears to lie in the application and integration of these established components to a specific dataset, rather than in the underlying methodology itself.

This is correct: the purpose of the current work is to show that physics-based differentiable modeling is now possible in coronagraphic exoplanet imaging for real, on-sky JWST NIRCam data, with significantly better sensitivity than standard methods in a diverse set of datasets. We emphasize that our framework has significant differences relative to prior similar approaches (in particular, Feng et al 2025 for the most similar approach: https://ui.adsabs.harvard.edu/abs/2025ITCI...11...36F/abstract) in that we incorporate more complex physical models and a multi-stage optimization. These two changes are critical to make this approach not only be effective on real data, but surpass standard approaches like KLIP on real data.

More concretely, the goals of this work are:

1) **To present, for the first time, the successful application of differentiable model-based PSF subtraction on coronagraphic on-sky exoplanet imaging data and demonstrate significantly superior performance to standard techniques on a diverse range of real, on-sky datasets. We have made this clearer on the Introduction of the Main text.**
2) **To share the method details and modifications made to make the method perform adequately on real data, contrary to the simulated data presented in previous work (e.g., in Feng et al 2025). In the manuscript, we have significantly expanded our discussion of the novelty of our work in relation to previous model-based PSF subtraction and autodiff-based PSF subtraction for coronagraphy. This is discussed in more detail now in the Introduction of the Main text, the Discussion of the Main text, the Future Improvement details section (section 4.2.1) in the Supplementary Discussion. We have added explicit comparisons in the Optimization design section (Section 2.1.2) in the Methods, where we carry "leave one out" experiments to show where the improvement in sensitivity of FLIP comes from, in relation to both KLIP and previous approaches like that shown in Feng et al 2025 (see our Figures 6 and 7).**
3) **To motivate further investigation of differentiable model-based methods in PSF subtraction, given the significant improvements in sensitivity presented in this**

work, and the great interest in the community in advances in PSF subtraction given the current limitations of JWST (e.g.,https://ui.adsabs.harvard.edu/abs/2018SPIE10698E..09P/abstract) and the active development of future instruments such as HWO where PSF subtraction is a critical component of the observatory performance (e.g., https://ui.adsabs.harvard.edu/abs/2024JATIS..10c4006S/abstract).

4) To share with the exoplanet imaging community a tool for improving the sensitivity of their own JWST observations, and thus better characterizing the performance of FLIP.

Specifically, for "[a] more explicit, quantitative, and controlled comparison to prior implementations of this approach", we have added a test comparing our framework to a similar implementation by Feng et al. 2025 in our "leave one out" ablation tests in the Optimization design section (Section 2.1.2) in the Methods. This is shown in newly-added Figures 6 and 7. We show how our new model with extra parameters, our calibration stage and our tuning steps are crucial to achieve the present performance.

We have made these points clearer throughout the manuscript.

C. Data & methodology

Beyond questions of novelty, a more fundamental limitation of the manuscript is the limited interpretability of the reported performance improvements. While the method demonstrates improved detection capability, the manuscript provides limited insight into the mechanisms responsible for these gains. It remains unclear which components of the pipeline are primarily driving the improvement, or under what conditions the method may succeed or fail. As a result, the approach appears effectively "black-box-like" in its current form, where performance gains are observed but not sufficiently explained in terms of underlying model behavior or physical intuition. This lack of mechanistic understanding is closely linked to the broader concern that the inverse problem is not rigorously validated.

We have further elaborated on the sources of sensitivity improvement provided by FLIP, both throughout the text and with dedicated testing. In particular, we illustrate the mechanistic source of improvement with "leave one out" experiments: see our "leave one out" ablation tests in the Optimization design section (Section 2.1.2) in the Methods, displayed in Figures 6 and 7. These showcase the effect that each choice of model parameters and of optimization stages produces in the achieved sensitivity improvement. In short, the flexibility of our model (contrary to Feng et al 2025) is necessary to model the PSF to high fidelity and overcome model-mismatch on real data, and the ability to fit

for arbitrary drifts between science and reference observations (contrary to KLIP-like methods) is what enables superior performance to KLIP-like methods.

D. Appropriate use of statistics and treatment of uncertainties

A further concern relates to the treatment of the underlying inverse problem. The manuscript indicates that the method estimates instrumental parameters, as well as source position and flux; however, no quantitative validation of these inferred parameters is presented. Given the strong degeneracies inherent in this class of problems, it is not demonstrated that the method is actually recovering physically meaningful parameter estimates, rather than primarily producing image-domain reconstructions that match the adopted metrics. In particular, good detection or reconstruction performance alone is not sufficient to establish that the inverse problem has been properly solved. This concern is further reinforced by the manuscript's own acknowledgment that the problem is ill-posed and degenerate, with multiple solutions potentially producing similar observational outcomes, which is deferred to future work rather than addressed in the present analysis. In this context, the interpretation of inferred parameters appears insufficiently supported.

We have further clarified these points throughout the text. See responses to specific comments below.

This is correct: the manuscript does not claim to be able to retrieve physically reliable physical parameters for the instrument with appropriate uncertainties. This is stated in the manuscript under the Future Improvements section in the Main text, and the Future Improvements Details section of the Supplementary Discussion. This is intentionally deferred to future work, since physically reliable solutions are not a requirement for improved planet detection sensitivity. In particular, in the Supplementary Discussion section we present several possible avenues to perform rigorous model calibration in future work.

This is exactly analogous to the KLIP algorithm: no physical intuition is ever provided behind, for instance, the number of principal components chosen for the subtraction, or the number of annuli and subsections used for the reconstruction, among other details. Similarly, no uncertainties are ever reported related to, for instance, the optimal choice of principal components ("optimal KL modes = 50 +- 10") in the high-contrast imaging community.

We present our method as a PSF subtraction algorithm: leveraging physical models and automatic differentiation to achieve higher planet detection sensitivity. Given the

physical nature of the model, it is a promising avenue for future work to perform calibration work and retrieve physically accurate model parameters. This is beyond the scope of the present work, which is presented as a tool for the high-contrast imaging community to improve the sensitivity of planet detection on various datasets.

Furthermore, the manuscript does make explicit several limitations and scenarios in which FLIP, in its present form, is sub-optimal (see Future Improvements in the Main text and Future Improvement Details in Supplementary Discussion). We discuss complex detector effects as a limitation, the presence of significant contaminants such as bright background galaxies or debris disks as a limitation, for example.

E. Conclusions: robustness, validity, reliability

E.1 Scientific overstatement (results interpretation)

The manuscript emphasizes potential scientific insights that could be derived from future analysis of these detections, rather than demonstrating such advances within the present work. As a result, the scientific impact remains largely prospective rather than realized. In particular, the claim of recovering "previously undetected planets" is not supported by the analysis presented, which is limited to re-detection of previously known objects in a different observational band.

We have removed the language about "previously undetected planets" for clarity, and rephrased it as the first detection of 51 Eri b at 4.3 micron.

To the broader point about prospective impact, we emphasize that we are showcasing a proof of concept in this work. To deliver scientific insights given the contribution of our work, we would need to: 1) use our method to re-analyze the physical properties of all detected companions from the four datasets presented and improve on their characterization, or 2) use our method to re-analyze all NIRCam coronagraphy data to search for new companions, and establish deeper non-detections. Both are beyond the scope of this work. But our ability to demonstrate improved sensitivity on a diverse set of NIRCam data, which is relevantly similar to a wide range of current and future JWST NIRCam datasets, shows that this technique indeed holds promise for NIRCam. Our work opens the door to these future analyses.

E.2 Unsupported methodological claims

A similar issue arises in several methodological statements that are insufficiently supported by quantitative evidence. For example, the claim that the technique

achieves exoplanet detection sensitivity “beyond the stability limit” is not convincingly demonstrated, as the stability limit is not clearly defined or quantitatively established, and no direct comparison is provided. Likewise, the statement that “four groups of free parameters are generally sufficient for PSF fidelity in most cases” is not supported by a systematic evaluation or sensitivity analysis and requires either quantitative justification or more careful qualification.

We have expanded our tests on both of these points (the stability limit and the free parameters). We have added explicit calculations of the stability-limited KLIP performance, which we now include in our contrast curves in Figure 3, and which we describe briefly in the Results section of the Main text and in detail in the “Estimation of the stability-limited sensitivity” section (Section 2.4) in Methods. These new calculations, based on realistic in-flight characterization of JWST stability and performance, show that FLIP achieves sensitivity beyond the stability-limited sensitivity that limits KLIP performance.

For each of the four datasets, we simulate synthetic observations that include only 1) the instrumental factors we refer to as “instability”, i.e., OPD drift and star-coronagraph misalignment differences between reference and science observations, and 2) the effects of algorithm over-subtraction intrinsic to KLIP. In all cases, our stability-limited KLIP performance on our simulated data is consistent with the KLIP performance on real data to better than a factor of 2. This supports our claim that KLIP is indeed limited by the factors we describe as instabilities, and thus shows that FLIP can perform beyond this limit.

In addition, we have qualified our claim about the number of “parameters being sufficient in most cases”, and provided further insight into this in our “leave one out” tests (“Optimization design” section in Methods, see Figures 6 and 7). This statement is intended as a remark on the work that each model parameter does in our method.

E.3 Robustness and completeness of experimental validation
The robustness of the reported results remains unclear. The manuscript presents a limited number of experiments without a systematic exploration of parameter space or observational conditions. As a result, it is difficult to assess whether the reported performance is representative and general, or whether it may depend on favorable or selectively presented cases. A more comprehensive and statistically grounded evaluation would be important to establish the reliability and generality of the method.

We have added explicit details about our choice of datasets (Results section of the Main text and the Experiments section of Methods). In summary, we carefully choose a representative set of datasets in a diverse variety of conditions and setups precisely to

show that the effectiveness of FLIP is not reduced to cherry-picked examples. We present four datasets that comprise:

1) **Three different physical systems (HIP 65426, 51 Eridani, TYC 5899),**
2) **Three different observational filters (F444W, F410M, F430M),**
3) **Two different coronagraph instruments (NIRCam 335R round coronagraph, and NIRCam LWBAR bar coronagraph)**
4) **Both real planets (HIP 65416 b and 51 Eridani b) and simulated planets (TYC 5899)**
5) **Observations with a dedicated reference star with a 9-POINT-CIRCLE dither pattern (HIP 65426), with a dedicated reference star with 5-POINT-BAR dither pattern (51 Eridani), and without dedicated reference stars but with a PSF library composed from a self-referenced survey (TYC 5899).**
6) **Observations with reference stars close in time to the science target (HIP 65426 and 51 Eridani) and far in time from the science target (TYC 5899).**
7) **Observations of host stars of various spectral types: A2V (HIP 65426), F0IV (51 Eridani) and M3 (TYC 5899).**

The set of observations we selected are representative of a wide variety of existing and future observations with JWST NIRCam coronagraphy. This has been elaborated on in the Experiments section (section 2.2) of Methods.

F. Suggested improvements / Specific major comments

-The authors should more clearly quantify the performance gains of the method relative to prior work, and clarify whether the advance represents a substantive step-change or an incremental improvement.

We have significantly expanded the discussion of prior work and the relation of our present work with them, primarily in the introduction, discussion and methods sections. In particular regarding previous uses of model-based PSF subtraction, and early experiments with autodiff-based PSF subtraction on simulated data for coronagraphy. We have more clearly stated that the particular implementation choices we make (a multi-stage optimization and use of automatic differentiation) are what enable this technique to work successfully on coronagraphic on-sky data for the first time. Furthermore, we do not just see incremental improvements relative to KLIP of 10% or 20%, but factors of several and up to an order of magnitude.

In that sense, it is a substantial step-change, since these implementation choices are the difference between a method working on on-sky data or not working on on-sky data, and between a method surpassing current techniques or not surpassing current techniques. In particular, the most similar approach presented thus far in the literature (Feng et al 2025 https://ui.adsabs.harvard.edu/abs/2025ITCI...11...36F/abstract), does not incorporate our multi-stage optimization, does not incorporate a broad-band optical model (only monochromatic simulated data) and does not incorporate a flexible enough physical

model. Our Figures 6 and 7 show that such a simplified approach is fundamentally insufficient to be effective on real on-sky data, either by being unable to subtract enough starlight, or by severely over-subtracting the planet signal. We have expanded on this too in the "Optimization design" section in Methods.

-"We recover previously undetected planets"
=> This claim should be revised for accuracy and consistency with the results presented.

We have changed this for clarity.

-"By explicitly modeling the physics of the instrument and leveraging optimization tools from the machine learning community, our technique FLIP achieves exoplanet detection sensitivity beyond the stability limit of JWST."
=> The statement regarding performance "beyond the stability limit" should be either quantitatively substantiated with a clear definition and direct comparison, or appropriately tempered.

We have added substantial discussion and quantitative calculations of the stability limit in our text and figures. See contrast curves in Figure 2, our brief description of these tests in the Results section of the Main text, and the detailed description of these tests in the "Estimation of the stability-limited sensitivity" section (Section 2.4) of the Methods section.

Additionally, we have emphasized throughout the text (Abstract, Future Improvements, Results and Discussion in the Main text) what the stability limit refers to in our text: primarily, phase drifts and telescope pointing errors (i.e., target acquisition errors, or star-coronagraph misalignment errors).

-"Given these models, we find that four groups of free parameters are generally sufficient for PSF fidelity in most cases"
=> The claim that four groups of free parameters are "generally sufficient" for PSF fidelity should be supported with quantitative evidence or more carefully qualified.

We have rephrased it to refer to our experiments.

-"Inferred instrument parameters: Coronagraphy is an ill-posed and degenerate problem, and as such there is a wide range of solutions that may produce effectively the same PSF estimate. Furthermore, the nominal STPSF models for

JWST NIRCam, which we use to initialize our differentiable model, are known to be imperfect. To use FLIP to retrieve realistic instrument parameters future calibration work is required.”
=> I couldn’t agree more and this is why I think this shouldn’t be bypassed but rather be part of this study. The manuscript should include a rigorous validation of inferred parameters including uncertainty estimates and comparison to simulations or ground truth, to demonstrate that the inverse problem is meaningfully solved. In addition, a more systematic and statistically robust exploration of performance across observational conditions and parameter space would be valuable to establish the generality and reliability of the method.

We have clarified the point (Future Improvements section of the Main text, Future Improvements details in the Supplementary Discussion, and editing our Figure 1 and its caption) about the role of FLIP as a way to effectively compensate for instrument instabilities in PSF subtraction, and highlighted that the method is currently not intended to be used as a tool to estimate systematically robust instrumental parameters yet (e.g., non-common path aberrations in JWST/NIRCam). Future calibration work will be needed for this application.

On the point about “a more systematic and statistically robust exploration of performance across observational conditions and parameter space”, we have described in detail the diversity of the datasets tested on this work (in the first paragraph of Results in the Main section and the Experiment section of the Methods), supporting how they are representative of a wide variety of datasets with JWST/NIRCam.

- “future work will use this detection to measure the 4.3 μm photometry of the planet and carry out a new atmospheric characterization” “These results are illustrative of the potential of FLIP to aid exoplanet discovery with JWST”
=> The manuscript would benefit from a clearer distinction between demonstrated results and future potential, with appropriate adjustment of the overall framing.

We have clarified this distinction throughout the manuscript, by more explicitly highlighting challenges of future work and current limitations (on the Future Improvements, Discussion and Future Improvements Details sections). We have been careful to explicitly state “future work will…” or “this will enable…” on points regarding future potential.

- Reproducibility is currently limited by execution and documentation issues, including reliance on a CUDA-enabled GPU environment without a CPU fallback

or sufficiently clear instructions. Providing a minimal runnable example and clearer computational guidance would improve accessibility and independent verification.

We have added a CPU fallback to the code. It is important to note that an essential part of the code is the ability to run it on GPU, since that is what significantly reduces computation time to a reasonable time. Just like one can technically train a neural network on CPU but it is not the optimal choice, the same applies here.

We have also clarified the README file with the right location of the script (not involving a "scripts" folder but having run_FLIP.py at the same level as the other script dependencies).

G. References

The manuscript appears to appropriately cite relevant prior work. However, the novelty relative to prior work is not always clearly articulated, particularly regarding differentiable modeling frameworks that appear to be the baseline for the present approach.

We have expanded on this in the Main section (Introduction and Discussion), and in the Methods (discussing future improvement details). We have paid especial attention to comparisons with Feng et al 2025 (https://ui.adsabs.harvard.edu/abs/2025ITCI...11...36F/abstract) which is the work we refer to when discussing differentiable modeling frameworks applied to coronagraphic data in simulations. Both in the Main and Methods sections, we have elaborated on the differences of our approach and how they are critical to making a differentiable modeling framework effective on real on-sky data. This is supported by our figures showing "leave one out" tests (Figures 6 and 7; see discussion in "Optimization design" in Methods) to illustrate the mechanisms behind our contrast improvement, as these also illustrate the critical role that our specific implementation choices make.

H. Clarity and context

The manuscript is clearly written and generally well structured. However, several claims in the abstract and conclusions overstate the demonstrated results. More broadly, there is a mismatch between the framing of the work and the results actually demonstrated, with emphasis placed on potential future scientific applications rather than on fully established findings.

We have reworded the manuscript at several points, restricting our conclusions to the scope of our experiments (e.g., focused on experiments with JWST/NIRCam, comparison with KLIP as one benchmark method, more accurate description on the 51 Eri b detection at 4.3 micron). We have added substantial references to prior work and context about the use of model-based PSF subtraction and autodiff-enabled techniques to better frame the contribution of this work (see Introduction and Discussion in the Main text, also Optimization Design in Methods, and Future Improvement Details in the Supplementary Discussion), and consequently discuss its impact on current and future high-contrast imaging observations.

Referee #3 (Remarks on code availability):

Supplemental materials review:

“”1. Instructions to run on data
- Run the following bash command inside a virtual environment with all the dependencies outlined above and with access to an NVIDIA A30 GPU (or equivalent):

```
./scripts/run_JWST_test_HIP65426.sh
```

=> While code is provided, the current instructions do not allow straightforward reproduction of the results. In particular, the main execution script does not run as described (there’s no “scripts” folder in the provided package). In addition, reproducing the results is not straightforward. The current implementation assumes a CUDA-enabled GPU environment and does not offer clear guidance on configuration for a CPU fallback. This limits the ability to independently validate the method and assess its robustness, particularly given the broader concerns regarding parameter inference and validation.

We have edited the README file for clarity in the execution instructions and we have added a CPU fallback to the code. The code now automatically checks if the machine has a CUDA GPU available, and if not, it defaults to a CPU. It is important to note that running the code on a CPU will be significantly slower, and that a GPU environment is a requisite for optimal performance.

We thank the reviewers for their constructive comments. Below we include a point-by-point response to the new suggestions.

Referees' comments:

Referee #2 (Remarks to the Author):

First, I would like to thank the authors for their excellent work in addressing all of my (our) comments. The manuscript has been substantially improved, both in terms of clarity and presentation. Overall, I am very happy with the revised version and believe that the authors have satisfactorily addressed my main comments. I also appreciate the authors' detailed responses to my questions and the additional clarifications provided both in their responses and in the revised manuscript.

I have a few minor corrections and comments that I believe require the authors' attention (mostly minor editorial and English-writing suggestions):

1) Lines 110–112:
“After over thirty-five years of community-wide efforts, this work is the first time that model-based PSF subtraction surpasses standard techniques in coronagraphic exoplanet imaging, with up to an order of magnitude improvement in sensitivity.”
I am wondering about the rephrased version. From my point of view, the sentence could be read as meaning that there have been more than 30 years of efforts and that this is the first occasion on which model-based PSF subtraction has surpassed standard techniques. However, I do not think this is exactly the intended meaning.
As I understand it, the intention is to highlight that, after more than 30 years of community-wide development of post-processing techniques for coronagraphic direct imaging of exoplanets, this work demonstrates for the first time that a model-based PSF subtraction approach can outperform the standard techniques in terms of sensitivity. I want to emphasize that the difference is that the phrase above sounds more like "there were multiple times in which the community tried to implement this, reaching worst sensitivities, and this is the first time the sensitivity is better than the ones from traditional techniques".
I have two suggestions, that keep the 30-year effort, the novelty, and the improvements, although the authors are of course free to rephrase the sentence as they prefer, as long as the core idea is conveyed more clearly:

Option 1: “After more than 30 years of development in coronagraphic exoplanet imaging, we demonstrate for the first time that a previously untested model-based PSF subtraction approach can outperform standard techniques, improving sensitivity by up to an order of magnitude.”
Option 2: “After more than 30 years of relying on standard techniques, we demonstrate that a previously untested model-based PSF subtraction approach can outperform them in coronagraphic exoplanet imaging, improving sensitivity by up to an order of magnitude.”

We rephrased this sentence, similar to Option 1. We also moved this sentence to the beginning of the Discussion section.

2) Line 176
The sentence “... is shown in the figure” would be clearer if the figure number were explicitly provided (Figure 2), for more comprehensive reading.

Changed.

3) Line 365
Figure 10 appears to be mentioned before Figures 8 and 9.

We have reordered Figure 10 (now figure 8) to appear before Figures 8 and 9 (now figures 9 and 10).

4) References
Some references appear to need updating, in particular references [26], [30], and [31].

We have updated references 25, 26, 30 and 31.

5) Figure colorbars
Figures 5, 6, 7, and 10 show “MJy/sr” or “[MJy/sr]” as the colorbar label. These are units and should be accompanied by the corresponding physical quantity. This is equivalent to putting “[arcsec]” alone in Figure 8 instead of “Separation [arcsec]”.
The label could be, for example, “Flux density [MJy/sr]” or “Surface brightness [MJy/sr]”, or “Counts [MJy/sr]”, as used in Figure 2. However, since the images are flux-calibrated, I would recommend the first option.
Figures 11 and 12 do not have colorbars. Although a colorbar is not strictly necessary here, including one would help the reader interpret the values shown in the images.

We have modified the color bar label to Figures 5,6,7 and 8 (formerly figure 10), and a color bar to Figures 11 and 12.

6) Figure captions
In general, the authors may wish to check the figure captions with the editor, as some of them appear to include information that would be more appropriate in the main text.
In my understanding, the caption should explain what is shown in the figure and provide the additional information needed to interpret the figure, plots, images, etc. The first sentence should briefly state what the figure shows, while the following text should describe the different elements of the figure and provide information that helps the reader understand it. Results, interpretations, and conclusions should generally be presented in the main text, in the paragraph where the figure is discussed, rather than in the caption. There may of course be exceptions, but I would recommend following this general structure and checking with the editor.

I will point out the ones I consider need further attention:

Figure 1: The first sentence is fine. However, the authors do not explicitly explain what panels (a) and (b) represent or what they show. The rest of the caption is understandable, but it should be more directly connected to the different elements of the figure. Alternatively, some of this information could be moved to the main text.

We have modified the caption of Figure 1 to more explicitly link the description of the methods to the elements of the figure.

Figure 2: This is, in my opinion, a good example of what the caption should contain.

Figure 3: The caption should be modified. For example, “Our stability estimate matches on-sky KLIP performance within a factor of 2” is more of a result than information needed to interpret the figure. Similarly, “At large separations, the stability limit can be below the noise limited depending on the exposure time of the observations” appears to be an interpretation of the result rather than a description of the figure. This can be inferred by the reader from the figure, and if it is important to highlight this point, I think it would be more appropriate to discuss it in the main text rather than in the caption.

We removed the statement about a factor of 2, and moved it to the corresponding paragraph in the Results section of the Main text. We have also removed the sentence about large separations, since it is discussed in the Methods section.

Figure 5: I suggest changing “(stage 1)” to “(stage 1, top images)” and “(stage 2)” to “(stage 2, bottom images)” so that the correspondence with the figure is immediately clear.

We edited the caption as suggested.

Figure 6: The sentence “We see that OPDs are crucial components in our model, both for general model flexibility and to account for differences between reference and science target. Star-coronagraph misalignment, while physically important, is likely absorbed by OPD terms in the form of tip-tilt aberrations, so they do not show important effects in the subtraction quality” is more of an interpretation/result than information needed to understand the figure. The interpretation should be presented in the main text (by “main text”, I mean the paragraph where the figure is cited). If the authors would like to retain some of this information in the caption, it could instead be rephrased to describe what is shown in the figure. For example:
“The images show a noticeable improvement when the OPD is included, as shown in panel X.”

We have removed the interpretation portion of the caption, since it is described in detail in the corresponding paragraph of the Methods section.

Figure 7: Similarly, the statement “We see that both OPDs are necessary for model flexibility, and that even with model flexibility, model calibration is essential to avoid overfitting” is an interpretation of the results rather than a description intended to help the reader interpret the figure. I suggest moving this statement to the main text or rephrasing it so that it describes the content of the figure.

We have removed the interpretation portion of the caption, since it is described in detail in the corresponding paragraph of the Methods section.

Figure 8: The statements “Therefore our performance is not strongly sensitive to the specific planet(s) we choose to track SNR with” and “We see that running the optimization for too long can cause significant degradation in SNR and increase in signal loss. Tracking injected planets successfully identifies this turning point, and ensures that our stopping condition is located within the SNR maximum or plateau for best sensitivity” are also interpretations of the results. I suggest moving these statements to the main text and keeping the caption focused on describing the figure and the quantities shown.

We have removed the interpretation portion of the caption, since it is described in detail in the corresponding paragraph of the Methods section, as suggested.

Figure 10: Similarly, the statement “We find that as long as our learning rates are within the correct order of magnitude, the PSF subtraction result is not strongly dependent on the specific choice of the learning rate (middle). The appropriate order of magnitude for the learning rates is obvious after a coarse search” is a result/interpretation rather than a description of the figure. I suggest moving this discussion to the main text and keeping the caption focused on explaining what is shown in the different panels.

We have removed the interpretation portion of the caption, since it is described in detail in the corresponding paragraph of the Methods section, as suggested.
